# A draft of the genome and four transcriptomes of a medicinal and pesticidal angiosperm *Azadirachta indica*

**DOI:** 10.1186/1471-2164-13-464

**Published:** 2012-09-09

**Authors:** Neeraja M Krishnan, Swetansu Pattnaik, Prachi Jain, Prakhar Gaur, Rakshit Choudhary, Srividya Vaidyanathan, Sa Deepak, Arun K Hariharan, PG Bharath Krishna, Jayalakshmi Nair, Linu Varghese, Naveen K Valivarthi, Kunal Dhas, Krishna Ramaswamy, Binay Panda

**Affiliations:** 1 Ganit Labs, Bio-IT Centre, Institute of Bioinformatics and Applied Biotechnology, Biotech Park, Electronic City Phase I, Bangalore, 560100, India; 2 Strand Life Sciences, Bellary Road, Hebbal, Bangalore, 560024, India

**Keywords:** *A. Indica*, Neem, Meliaceae, Genome, Transcriptome, Repeats, Phylogeny, Terpenoid biosynthesis, Pesticide and transcript expression

## Abstract

**Background:**

The *Azadirachta indica* (neem) tree is a source of a wide number of natural products, including the potent biopesticide azadirachtin. In spite of its widespread applications in agriculture and medicine, the molecular aspects of the biosynthesis of neem terpenoids remain largely unexplored. The current report describes the draft genome and four transcriptomes of *A. indica* and attempts to contextualise the sequence information in terms of its molecular phylogeny, transcript expression and terpenoid biosynthesis pathways. *A. indica* is the first member of the family *Meliaceae* to be sequenced using next generation sequencing approach.

**Results:**

The genome and transcriptomes of *A. indica* were sequenced using multiple sequencing platforms and libraries. The *A. indica* genome is AT-rich, bears few repetitive DNA elements and comprises about 20,000 genes. The molecular phylogenetic analyses grouped *A. indica* together with *Citrus sinensis* from the *Rutaceae* family validating its conventional taxonomic classification. Comparative transcript expression analysis showed either exclusive or enhanced expression of known genes involved in neem terpenoid biosynthesis pathways compared to other sequenced angiosperms. Genome and transcriptome analyses in *A. indica* led to the identification of repeat elements, nucleotide composition and expression profiles of genes in various organs.

**Conclusions:**

This study on *A. indica* genome and transcriptomes will provide a model for characterization of metabolic pathways involved in synthesis of bioactive compounds, comparative evolutionary studies among various *Meliaceae* family members and help annotate their genomes. A better understanding of molecular pathways involved in the azadirachtin synthesis in *A. indica* will pave ways for bulk production of environment friendly biopesticides.

## Background

*Azadirachta indica* A. Juss (neem) is an evergreen tree native to the Indian subcontinent [1] belonging to the family *Meliaceae* and order *Rutales*. The neem tree is extensively studied for its natural products. Neem oil and its limonoids such as azadirachtin, nimbin, salannin among others are of substantial economic value due to their wide array of applications in agriculture [2], healthcare [3] and soil conservation [4]. Azadirachtin, isolated half a century ago, has been intensively studied and its commercial formulations have been found to be toxic against a large range of insect species, whilst retaining very low mammalian toxicity [5]. Recent success in the total synthesis of azadirachtin [6] has renewed interest towards its commercial exploitation. Neem-derived azadirachtin and other limonoids are also used as anti-proliferative [7], cytotoxic [8-10], larvicidal [11,12], and anti-inflammatory [7,13] agents, suggesting the need for better understanding of molecular pathways involved in their synthesis.

In spite of the varied uses of azadirachtin and other neem-derived limonoids, a modern agro-chemical and/or pharmaceutical program focusing on understanding their molecular mechanism(s) of action is yet to be established. A better understanding of the biology of differentiation of secretory cells known to harbor azadirachtin and other triterpenoids [14] may permit development of varieties with higher percentage of these cells in the cotyledons. This information may also be exploited in stalling or delaying further differentiation of these cells to permit greater accumulation of the terpenoids of interest. The elucidation of complete pathways leading to terpenoid biosynthesis and expression of genes involved in such pathways in *A. indica* will pave ways towards development of newer terpenoid-based biotechnological applications.

*De novo* sequencing and assembly of the transcriptome from *A. indica* fruit was reported previously [15]. This report extends the earlier study to cover the whole genome and transcriptomes from root, leaf, stem and flower of neem. Comparative genomics among the *Meliaceae* family members will be enabled and, cheaper and environment friendly biopesticides may result using results presented in this study.

## Results

### Genome assembly and similarity with other plant genomes

**Figure 1:**
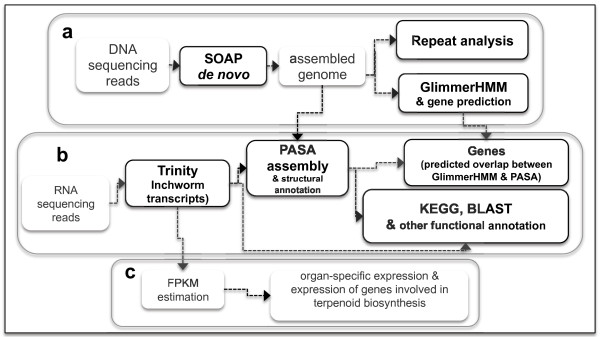
**Schematic representation of various analytical tools used in *A. indica *genome and transcriptome assembly, annotation and expression analysis.**

**Figure 2:**
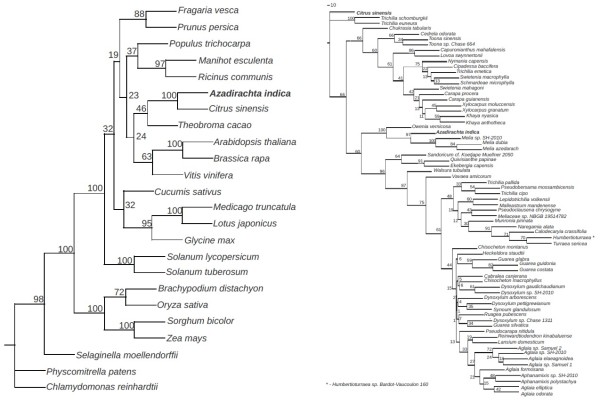
**Molecular phylogeny of *A. indica *with plants of other family (a) and within the *Meliaceae *family (b).** Individual rbcL and rbcS alignments (*.phy) were concatenated and bootstrapped using 100 replicates (**a**) through 'seqboot' in PHYLIP version 3.69 [22]. For intra-family phylogeny, rbcL sequences were bootstrapped (**b**). Phylogenies for each replicate was reconstructed using the 'dnaml' option in Phylip, after choosing either *Chlamydomonas reinhardtii* (**a**) or *Citrus sinensis* (**b**) as outgroups. The consensus phylogeny with bootstrap values highlighting the branch support was plotted using 'consense' and 'drawgram' options in Phylip.

Sequencing libraries generated using multiple platforms and chemistries were tested to assemble the neem genome and transcriptomes (Table 1, Table S1 in Additional file 1). Various combinations of sequencing libraries were used to obtain the best scaffold N50s and N90s using SOAPdenovo [16]. The scheme for *A. indica* genome assembly is given in Figure 1a. The *de novo* assembly of *A. indica* genome produced a scaffold N50 length of 452,028 bp with corresponding scaffold N90 length of 56,222 bp, contig N50 length of 740 bp and contig N90 length of 172 bp. The genome size of neem was determined to be 364 Mbp using the kmer frequency plot (Figure S1 in Additional file 2) representing 95% of the total published genome size [17]. Chargaff's symmetry rule was applied for assessing the quality of the genome assembly [18-21] by comparing the symmetry of 4-mers between the *A. indica* scaffolds and the *Arabidopsis thaliana (A. thaliana)* genome. There was a tighter distribution of symmetry in *A. indica* at around 0.5 (Figure S2 in Additional file 2) compared to the *A. thaliana* suggesting good assembly results. Genome scaffolds (>N50) mapping with *Citrus sinensis* (*C. sinensis)* and *Citrus clementina* (*C. clementina)* showed conserved macrosynteny between neem and citrus (Figure S3 in Additional file 2) but not with chromosomes from *A thaliana*, *Oryza sativa* (*O sativa*), *Theobroma cacao* (*T cacao)*, *Vitis vinifera* (*V vinifera), Ricinus communis* (*R communis*) and *Sorghum bicolor* (*S. bicolor*) (Figure S3 in Additional file 2). In *A. indica*, 385 and 435 scaffolds (>N50) out of a total of 534 scaffolds (>N50) mapped to *C. sinensis* and *C. clementina* genome scaffolds (>N50), respectively, with an Expect value of zero. Phylogenetic bootstrap analysis [22] corroborated the taxonomic closeness of *A. indica* to *C. sinensis*, as indicated by 100% bootstrap value using *rbcL* and *rbcS* (Figure 2a). Phylogenetic analysis within the *Meliaceae* family grouped *A. indica* with *Owenia* and *Melia* sp. with a 100% bootstrap value (Figure 2b). Like *A. indica*, *Melia azadirach* is known to bear bioactive limonoids [23] suggesting a common evolutionary trajectory with regard to synthesis of these compounds.

**Table 1:** ***de novo *assembly statistics**

				
a.				
Longest Contig	10111			
Contig N50	740			
Contig N90	172			
Longest Scaffold	3641215			
Scaffold N50	452028			
Scaffold N90	56222			
Number of Scaffolds	9714			
b.				
	Root	Leaf	Stem	Flower
Reads	4566554	4372192	5429138	5058312
kmers	57670643	55530204	67596347	60219435
Inchworm contigs	148819	148700	172507	150126
Total transcripts	27916	27369	34518	31223
c.				
Features		Average	S.D.	
Number of genes		20169	-	
Gene length		1695.95	1526.01	
Exon length		208.28	210.62	
Intron length		388.62	393.41	
Number of exons		3.5	2.96	
Number of introns		2.5	2.96	
Exonic GC%		42.74	5	
Intronic GC%		30.04	7.09	

The respective assembly statistics are summarized from various sequencing libraries using (a) SOAPdenovo [16] for the genome and (b) Trinity [35] for the transcriptomes. Various annotation statistics for genes, exons and introns are also provided (c).

### Repeat analyses

Repeat Modeler (*RepeatModeler Open-1.0*, 2008–2010) [24] and Repeat Masker (*RepeatMasker Open-3.0*, 1996–2010) [25] were used to determine the *de novo* repeats and to perform homology-based repeat analysis respectively among *A. indica* genome repeats. For repeat analysis, we used consensus repeat libraries (of *R.communis *[26], *Glycine max (G.max)*[27] and *S. bicolor *[28]), in-built plant specific libraries, Repbase [29] and the *de novo* neem repeat library constructed using Repeat Modeler. In addition, LTR-finder [30], TransposonPSI [31] and MITE-hunter [32] were used to study repeats in the *A. indica* genome. The neem genome was found to harbour fewer repeat elements compared to other sequenced eudicots with a total non-redundant repeat content of 13.03%. We further classified the repeats into two categories, the interspersed elements (11.21%) comprising of retrotransposons (3.72%) and DNA transposons (0.75%), and the tandem repeats (3.58%) comprising of the low complexity repeats (3.17%), simple repeats (0.05%), and the satellite repeats (0.38%) (Table S2 in Additional file 1). The detected interspersed repeat sequences identified using different tools (with similarity of at least 80% and more than 200 nucleotides long) were clustered using Vmatch [33], giving rise to a consensus neem repeat element library of length 23,920,944 bp (~6.59% of the genome).

**Figure 3:**
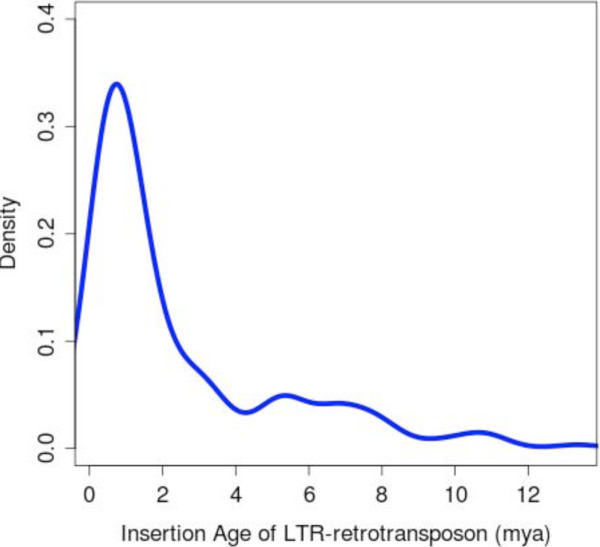
**The frequency distribution of LTR-retrotransposon insertion ages in *A. indica*.**

We studied the insertion age distributions among the LTR-retrotransposons in *A. indica* made by the Kimura 2-parameter distance matrix [34] and inferred that the oldest insertion event in neem dated back to 13.35 million years (Figure 3). The frequency of insertion events peaks around 1 million years ago with smaller secondary peaks observed at ~6 million and ~11 million years ago respectively.

### *De novo* transcriptome assembly

The scheme for transcriptome assembly and annotation is provided in Figure 1b. The transcriptomes of different organs were independently assembled using Inchworm (a part of the *de novo* transcriptome assembler Trinity [35]). Trinity performs better in comparison to other *de novo* transcriptome assembly programs for a wide range of parameters like genome complexity, read coverage and spliced isoforms [35,36] and hence was a natural choice for *A. indica* transcriptome assembly. Chimeric transcripts observed during Inchworm assembly [37] were filtered using PASA (Program to Assemble Spliced Alignments) [38]. The PASA-filtered transcripts were then anchored to the genome scaffolds by assembling overlapped transcript alignments into maximal alignment assemblies in order to remove multiple spliced isoforms.

### Transcript annotation and gene prediction analysis

**Figure 4:**
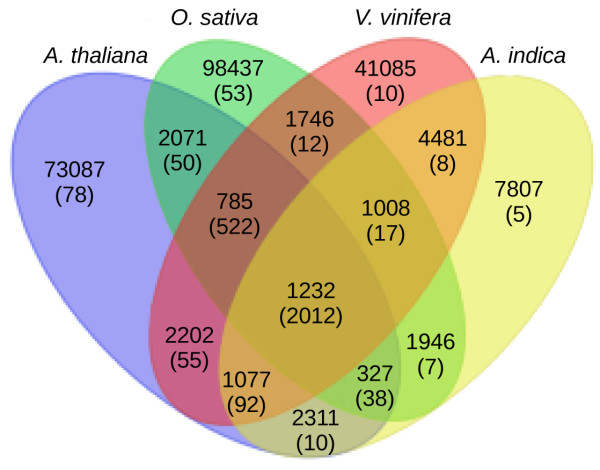
**Gene annotations and comparison across species.** Predicted genes in *A. indica* were used for homology-based search against the nr database restricting the hits to the green plant gene identifiers. Similar analyses were performed using the annotations in *A. thaliana*, *V. vinifera* and *O. sativa.* The overlapping gi list were used to draw the Venn diagram.

The transcriptome assemblies were annotated using the KEGG Automatic Annotation Server (KAAS) [39]. In addition to the KAAS, the assemblies were serially annotated using; MegaBLAST [40] against the non-redundant nucleotide database, BlastX against the non-redundant protein database, MegaBlast against the RefSeqRNA, Expressed Sequence Tag (EST), Transcriptome Shotgun Assembly (TSA) databases [40] and AutoFACT [41] using uniref90 and uniref100 [42], kegg [43], cog [44] and nr [40] databases (Table S3 in Additional file 1). In total, 380,629 common and 1,374 unique KEGG annotations (KO terms) across all organs (Figure S4 in Additional file 2) were observed. Annotated/predicted genes in *A. indica* were compared with a non-redundant database containing green plants gene identifier (gi) lists using homology-based searches. As shown in Figure 4, a higher percentage of overlap was found between *A. indica* and *V. vinifera*, a plant known to express a host of terpernoids [45]. As expected, genes common between *A. indica* and other species compared (*A. thaliana*, *O. sativa*, *V. vinifera* and in four sequenced organs of *A. indica*) had the highest number of KEGG annotation associated with them (Figure 4). The transcripts from all organs were further assigned Gene Ontology (GO) terms using Blast2GO [46] (Figure S5 in Additional file 2 and Table S4 in Additional file 1).

The *A. indica* RNA-Seq reads were aligned to the genomes or genome scaffolds of multiple plant species. The alignments of *A. indica* RNA-Seq reads to *C. sinensis* and *C. clementina* genome scaffolds by TopHat [47] and CuffLinks [48] suggest sequence similarity between neem and citrus (Table S5 in Additional file 1). This is in agreement with the results obtained from our phylogenetic analysis (Figure 2a). We built training sets using *C. sinensis* and *C. clementina* gene models and used GlimmerHMM [49] to subsequently predict genes in *A. indica*. Additionally, we used the *A. thaliana* training sets to represent a distantly related species for gene prediction analysis*.*

GlimmerHMM [49] predicted greater number of gene models in *A. indica* using *C. sinensis* (34,624) and *C. clementina* (34,737) training sets compared to *A.thaliana* (23,397). The predicted genes were then serially annotated using Megablast [40] and TblastX [40] (with Expect value of 10^-10^) resulting in 22,760 and 22,840 annotations with *C. sinensis* and *C. clementina* respectively. Statistics for PASA [38] predicted mapped transcripts, gene structures and assembly is provided in Table S6 in Additional file 1. The overlap between the GlimmerHMM and PASA predicted gene models was marginally higher using the *C. clementina* training set compared to the *C. sinensis* training set (Figure S6 in Additional file 2). The genes predicted by GlimmerHMM using *C. sinensis* training set and training set candidates predicted by PASA identified 10,667, 10,965, 12,209 and 11,181 genes expressed in the root, leaf, stem and flower, respectively (Figure S6c in Additional file 2).

### Nucleotide composition, transcript expression and gene structure analysis

The nucleotide composition analyses in the transcriptome from all organs suggested different Adenine (A), Thymine (T), and Guanine (G) Cytosine (C) content distribution. The A + T content of most transcripts ranged between 20-40% while for G + C there were two distinct populations of transcripts, one with 0-20% and the other with 20-40% (Figure S7 in Additional file 2).

**Figure 5:**
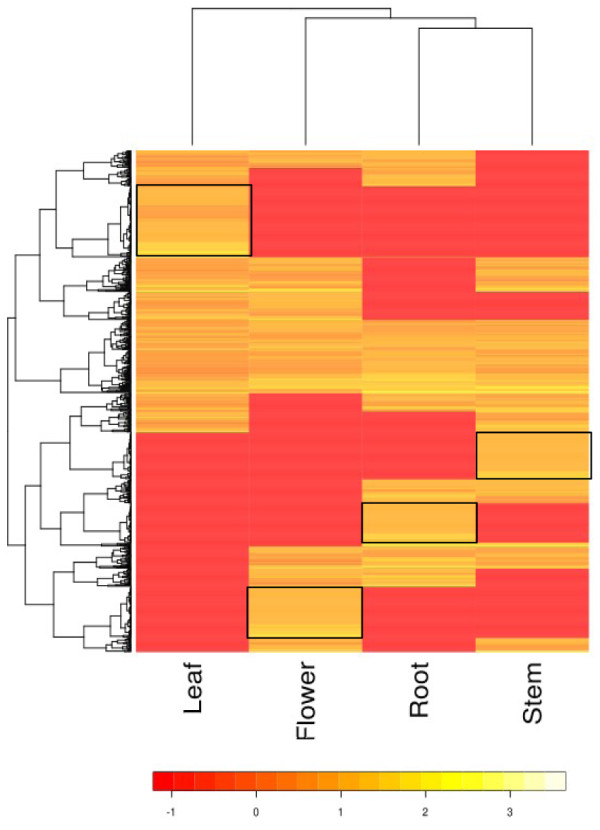
**Organ-specific expression for un-annotated transcripts.** KEGG annotations obtained for transcripts from all organs were parsed to retain only the un-annotated ones. PASA assemblies, establishing correspondence between transcripts and common genomic loci were obtained for the same. The top 300 highly expressed transcripts for each organ were chosen, filtered for redundancies, to yield a total of 888 unique assemblies and plotted.

Fragments per transcript kilobase per million fragment (FPKM) values derived from Trinity transcriptome assembly pipeline were used for expression analysis in different organs of neem (Figure 1c). The FPKM values for transcripts with overlapping peaks at the same level of expression in all organs were plotted (Figure S8 in Additional file 2). The frequency histograms plotted after binning the transcripts suggested maximum density around ~40% GC with 5.0-5.5 log_2_ (FPKM) values (Figure S9 in Additional file 2). In order to avoid erroneous expression analysis resulting from genes with multiple isoforms, the PASA-assembly pipeline were used to remove them and infer relative abundance of transcripts in different organs (Figure 5).

**Figure 6:**
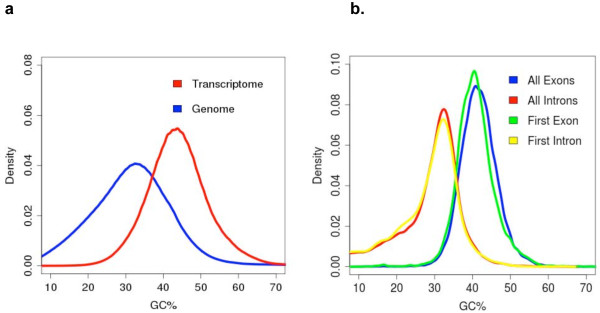
**GC density profiles.** The %GC compositions are plotted as density curves, (**a**). for the raw sequencing reads from the neem genome and pooled transcriptome from all organs and (**b**) for all exons, all introns, first exon and first intron from the gene structures assembled using PASA, corresponding to the pooled organ transcriptome.

**Figure 7:**
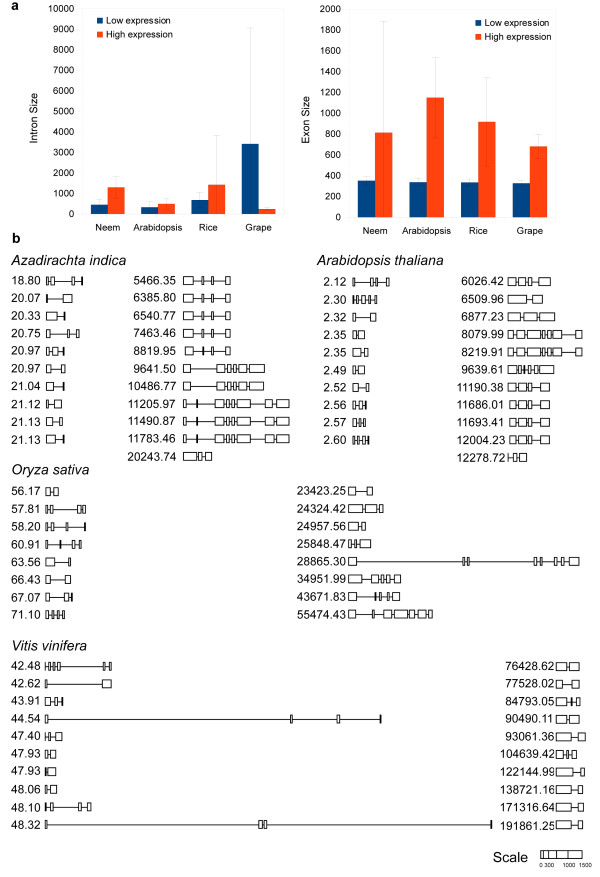
**Correlation between expression and gene structure.** (**a**) The average intron and exon sizes of the ten most and least expressed genes bearing KEGG Orthology assignments, from *A. indica*, *A.thaliana, O.sativa* and *V.vinifera* are plotted separately for the low and high expression level categories. The error bars indicate standard deviation in the intron and exon sizes respectively. These sizes were obtained from gene structures provided by PASA assemblies mapping these genes to corresponding genome scaffolds. (**b**) The gene structures depicting the organization and sizes of exons (boxes) and introns (lines) are represented using the web tool StrDraw, for the above mentioned low (left panel) and high (right panel) transcript expression categories in all species.

Post-annotation, GC content comparisons were made between the genome versus transcriptome, introns versus exons and first introns versus first exons (Figure 6). The raw genomic reads were relatively GC-poor and the exons were relatively GC-rich (Figure 6). The genes those were inferred to accumulate to higher abundances in neem harbored longer introns when compared to the ones in *A. thaliana*, *O. sativa* and *V. vinifera* (Figure 7). Although, there was variation in the exon size, similar correlation between the cumulative exon length and expression was observed.

### Comparative analysis of transcript expression

In neem, transcript abundances (TAs) were compared with their orthologs from other species after inter-species normalization of the expression values with elongation factor 1-alpha (EF1A; NCBI gene ID: 836161). Among the 88 genes that were differentially abundant in neem, transcripts from three genes (GGPS, malZ and polygalacturonase) were expressed at a higher level in *A. indica* compared to the other three species studied (Table S7 in Additional file 1). GGPS (geranylgeranyl diphosphate synthase type II, NCBI gene ID: 816377) is involved in terpenoid backbone synthesis, malZ (alpha-glucosidase, NCBI gene ID: 823737) in galactose metabolism and polygalacturonase (NCBI gene ID: 820815) in starch and sucrose metabolism. Unlike GGPS, no other consensus gene was over-expressed to the same magnitude (<10-fold) in neem when compared to the other three species studied. A complete list of the genes and their relative TAs in species is provided in Table S7 in Additional file 1.

### Terpenoid biosynthesis pathways

**Figure 8:**
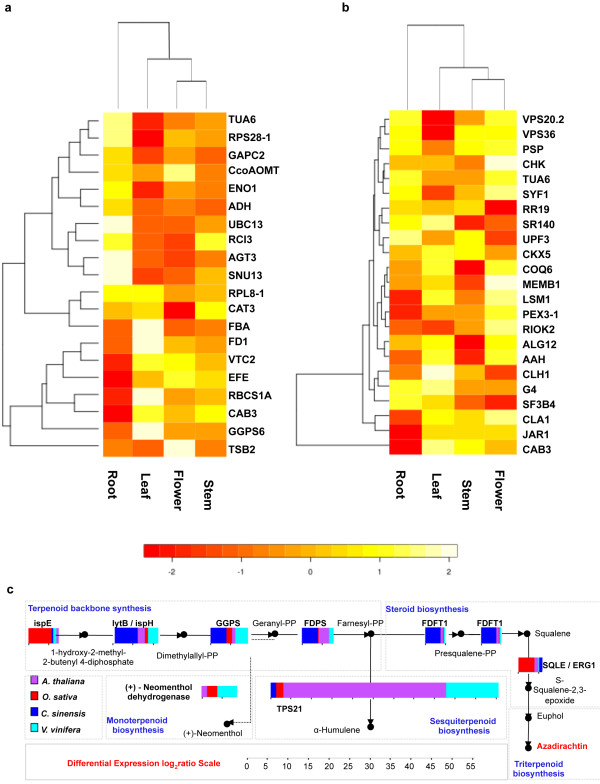
**Genes involved in the terpenoid biosynthesis pathways.** Organ-wide transcript abundance heat-maps were drawn for top (**a**) and bottom (**b**) five transcripts in each organ. The clustering and heat-map analyses were done using R. Differential transcript abundance were calculated between terpenoid pathway related genes expressed in *A. indica* leaf to those in leaves of *A. thaliana*, *C. sinensis* and *O. sativa*, and *V. vinifera*, after normalizing the transcript abundance of all genes to that of elongation factor 1-alpha (EF1A). The differential indices were further log_2_-transformed and represented as cumulative bar histograms for all species (**c**).

The genes related to quinone, terpenoid and terpenoid-backbone synthesis pathways were identified by KEGG’s KAAS automatic pathway annotation pipeline [39]. *GGPS* (NCBI gene ID: 816377), *COQ6* (NCBI gene ID: 822006) and *CLA1* (NCBI gene ID: 827230) were among the top 5 differentially expressed genes in neem leaf compared to the other organs (Figure 8). Eight genes (*TPS21*, NCBI gene ID: 832461; *lytB/ispH*, NCBI gene ID: 829585; *ispE*, NCBI gene ID: 817234; *GGPS*, NCBI gene ID: 816377; +neomenthol dehydrogenase, NCBI gene ID: 825294; *FDPS*, NCBI gene ID: 827430; *FDFT1*, NCBI gene ID: 829616 and *SQLE*, NCBI gene ID: 816814) involved in the synthesis of sesquiterpenes and triterpenes leading to azadirachtin-A were over-expressed in neem compared to *A. thaliana*, *O. sativa, C. sinensis* and *V. vinifera*, (Figure 8c). The length of introns in the terpenoid and steroid biosynthesis gene families in *A. indica* were greater compared to the ones in *A. thaliana*, *O. sativa, C. sinensis* and *V. vinifera* (Figure S10 in Additional file 2).

## Discussion

The current study describes the draft genome and transcriptomes from root, stem, leaf and flower*.* Both neem and citrus belong to the Order *Rutales* and our phylogenetic studies reaffirmed their taxonomic closeness. Additionally, phylogenetic studies grouped *A. indica* with the *Melia* species, one that is also known to harbour bioactive compounds (Figure 2b) suggesting a common evolutionary process with regard to synthesis of these compounds in *Meliaceae.* Repeat analysis showed low repeat content in *A. indica* genome compared with other sequenced angiosperms. This could have been due to the presence of xenobiotic terpenoids specific to the plant, which might have been a major impediment for horizontal gene transfer [50]. This impediment in horizontal gene transfer could have skewed the conventional host-pathogen interactions countering the accumulation of repeats in *A. indica* compared to other contemporary angiosperms. Insertion times of LTR elements in neem suggest a major wave of retro-transposition about 1million years ago (Figure 3), similar to the observation made in *S. bicolor*[28]. However, in *A. indica* the oldest insertion event date back much further than *S. bicolor* (13.35 million years in neem against 3 million years in *Sorghum*, Figure 3). Monocot genomes have higher GC content than dicots, which is reflected in an average difference in codon usage between them [51,52]. Interestingly, like *O. sativa *[53] but unlike *A. thaliana* and others [54], the *A. indica* genome shows a bimodal distribution of GC content (Figure S11 in Additional file 2). Domesticated crop genomes with higher GC content are likely to have evolved by a relaxation of a natural selection process against higher nitrogen use in DNA due to the use of nitrogen-rich fertilizers [55]. *A. indica*, a hardy plant, is not domesticated and can grow in poor and degraded soil including in semi-arid conditions with tolerance for high temperature [56]. Additionally, there is no history of any usage of nitrogen rich fertilizers to grow *A. indica*. This might have played a role in shaping the AT-rich nucleotide composition of *A. indica* genome.

Transcript abundances of genes in *A. indica* correlated well with their functions in respective organs; for example, root, leaf, stem and flower showed the presence of highly expressed ones involved in ion homeostasis, photosynthesis, ion transport and ATPase activity, respectively. Unlike the genome, *A. indica* transcriptomes exhibited unimodal GC distribution roughly overlapping with the higher GC peak of the genome (Figure S11 in Additional file 2). The distribution of A + T in *A. indica* transcriptomes mapped closer to the *A. thaliana* transcriptome than to the ones in *O. sativa* or *V. vinifera* (Figure S11 in Additional file 2). The lengths and the GC content of the first exons and first introns are reported to be associated with functional elements in other large genomes [57]. The GC content of the first exons and first introns in *A. indica* were similar to that of other exons and introns in the genome (Figure 7). Highly expressed primary transcripts of *A. indica* bear longer intron structures suggesting their possible functional role in expression and conforming with the results shown in other plants [58] and yeast [59].

Sequencing and analysis of the genome and transcriptomes of neem holds significance due to the plant’s utility in agriculture and medicine. The use of botanical products as pest control and deterrence has acquired greater significance with the shifting trends in mainstream agriculture towards sustainable and organic farming. Neem provides a suitable option for developing such eco-friendly and sustainable pesticides [56]. Currently a very small percentage of farmers use neem-based products as a substitute to synthetic pesticides as the general awareness of such practices remains limited. Azadirachtin, derived from neem seed kernels, has been proved to be a potent and effective pesticide. Total chemical synthesis of azadirachtin is challenging owing to the low yield of synthesis and remains a bottleneck for commercial bulk manufacturing. Secondary metabolites synthesized by terpene synthases are the major components of essential oils and are of great economic value. In plants, synthesis of terpenes is compartmentalized into monoterpenes and diterpenes (as well as carotenoids and chlorophylls) that are produced via the 1-deoxy-d-xylulose-5-phosphate (DXP) pathway in the plastids while the sesquiterpenes and triterpenes are made in the cytosol via the mevalonate pathway [60]. The enzymes involved in the synthesis of azadirachtin have not been studied in detail. The pathway hierarchy in neem-derived tetranortriterpenoid azadirachtin-A primarily requires the synthesis of a terpenoid backbone followed by steroid and triterpenoid biosynthesis. We found that the genes in the terpenoids and steroid biosynthesis family (TPS21, lytB, ispH, ispE, GGPS, +neomenthol dehydrogenase, FDPS, FDFT1 and SQLE) were more abundant in neem compared to *A. thaliana*, *O. sativa, C. sinensis* and *V. vinifera*.

This study may help close the gap between traditional knowledge and current practices in the agrochemical industry. The progressive increase in the cost of petroleum-based starting materials has led to a surge in the price of synthetic pesticides scourging the meager profit margins presented to the farmers in developing countries. This study may provide a cost-effective alternative by aiding biotechnological research efforts in enhancing disease resistance in plants.

## Conclusions

The *de novo* sequencing and analyses of the draft genome and organ-specific transcriptomes of neem plant, *Azadirachta indica* is reported*. A. indica* is the first *Meliaceae* family member to be sequenced. The neem genome bears fewer repetitive elements compared to other sequenced higher plants. It has about 20,000 genes with an average transcript length of 1.69 kbp. *A. indica*’s evolutionary closeness to *Citrus* species was verified by both molecular phylogenetic analyses and sequence similarity. Transcript expression and the exon-intron junction architecture of underlying genes involved in the terpenoid biosynthesis pathways suggested relative abundance of enzymes involved in the azadirachtin synthesis in neem. Genes involved in the terpenoid biosynthesis pathways in neem bear longer introns compared to the same genes in *A. thaliana, O. sativa, V. vinifera* and *C. sinensis*.

## Methods

### Sample collection, identification, nucleic acids extraction and quality control

Different parts of *Azadirachta indica* A. Juss were collected from a locally grown tree. The genus and species was confirmed at the South Regional Centre, Botanical Survey of India, Tamil Nadu Agricultural University Campus, Coimbatore, Tamil Nadu, India using the herbarium of the twigs bearing flowers and fruits. All parts of the plant (root, leaf, stem, and flower) were collected and immediately flash frozen in liquid nitrogen and stored at -80°C until further use. Total genomic DNA was extracted using Qiagen plant genomic DNA extraction kit. Total RNA was extracted using Plant Total RNA extraction kit (Bioteke, China). The quality, quantity of genomic DNA and integrity of total RNA were accessed using Nanodrop, Qubit methods and Agilent Bioanalyzer RNA 6000 Nano chip respectively (Text S1 in Additional file 3) before using the DNA and RNA in making sequencing libraries.

### Sequencing library preparation

#### Solexa sequencing-by-synthesis

Short-insert paired-end sequencing libraries were prepared using Illumina (San Diego, California, USA) TruSeq library preparation kit with one modification. The library was not amplified post-adapter ligation to minimize amplification-related bias and the un-amplified, adapter-ligated library was directly used to generate clusters on cBOT instrument following the manufacturer’s recommendation. Long-insert mate pair sequencing libraries were prepared using multiple inserts (10 kbp, 3 kbp and 1.5 kbp) using Illumina mate pair library preparation kit. RNA-seq sequencing libraries from all the four organs were prepared using Illumina TruSeq-RNA library prep kit following the manufacturers instructions. Details of the methods including the QC of samples and sequencing libraries are provided in the Text S1 in Additional file 3.

### Pyro-sequencing using IonTorrent Personal Genome Machine (PGM)

Genomic DNA of neem was sheared to 200 bp using Covaris (Woburn, Massachusetts, USA) to make libraries using IonTorrent PGM protocol. Sequencing was performed following the manufacturer’s instructions. Details of the methods including the QC of libraries are provided in the Text S1 in Additional file 3.

### Cloning of neem genomic DNA

Genomic DNA (2-11 kbp) was cloned into pJAZZ-OC linear cloning vector using BigEasy v2.0 kit from Lucigen (Middleton, Wisconsin, USA) following manufacturer’s specifications. Following cloning, positive colonies were picked and sub-cultured. Plasmid DNA was isolated and checked for inserts by restriction digestion (Text S1 in Additional file 3). Positive clones were selected and used for capillary sequencing.

### Capillary sequencing

Neem genomic DNA clones were used for capillary sequencing using BigDye Terminator kit using the manufacturer’s instructions. Positive clones were selected and used for sequencing in either forward or reverse direction using M13 primers using the Applied Biosystems 3500 instrument (Text S1 in Additional file 3).

### *De novo* genome and transcriptome assembly

Two sets of Illumina short insert (150 bp and 350 bp) paired-end libraries and three long insert (1.5 kbp, 3 kbp and 10 kbp) mate-pair libraries, capillary Sanger sequencing reads generated from cloned neem genomic DNA and pyro-sequencing reads generated with IonTorrent Personal Genome Machine (Table S1 in Additional file 1) were used in assembling the neem genome with SOAPdenovo [16]. We assembled the neem transcriptomes from all organs using the raw RNA-seq paired reads from four organs: root, leaf, stem and flower using the genome-independent transcriptome assembler Trinity [35].

### Phylogenetic analyses

The concatenated alignment of either the *rbcL* and *rbcS* genes of 24 plant species including neem or rbcL gene alone for *Meliaceae* family members were used to generate a bootstrapped phylogeny using Phylip v3.69 [22]. For reporting the phylogenetic analysis results, we followed the “Minimal Information About a Phylogenetic Analysis” (MIAPA) standard [61]. Detail attributes and the actual files are provided in Additional files 4, 5 and 6.

### Repeat identification and analyses

Repeat Modeler (*RepeatModeler Open-1.0*, 2008–2010) [24] Repeat Masker (*RepeatMasker Open-3.0*, 1996–2010) [25], LTR_Finder [30], TransposonPSI [31] and MITE-Hunter [32] were used to detect, identify and characterize repeats in the neem genome. Vmatch [33] was used to cluster repeat sequences and build a consensus (>80% similar) neem repeat library.

### Calculation of LTR insertion age

5' and 3' LTR sequences of each LTR-retrotransposon, identified by LTR finder [30], were aligned using ClustalW MPI [62]. The distances between 5' and 3' LTR sequences for each alignment were calculated using the Kimura 2-parameter distmat tool from EMBOSS package [63]. The insertion ages were further calculated from these distance values according to the formula [28] T = K / (2r), where T is the insertion age in years, K = Kimura distance value and r is the substitution rate per site per year (taken to be 1.3 × 10^-8^ as found in *O. sativa *[64].

### Nucleotide composition and expression analyses

The nucleotide composition of the transcripts from the neem organs and genome scaffolds/chromosomes of the neem, *A. thaliana*, *O. sativa* and *V. vinifera* were plotted as frequency histograms and density curves. The fragments per transcript kilobase per million fragments (FPKM) provided by Trinity were log_2_ transformed and plotted as frequency histograms for each organ, before and after categorizing the transcripts based on their G + C composition.

### Transcript annotation, gene prediction and inter-species comparison analyses

The transcripts were serially annotated for each organ using similarity-based BLAST analyses (Text S1 in Additional file 3) and functionally classified using BLAST2GO analyses. Gene predictions were performed using GlimmerHMM [49] and PASA [38]. Inter-species gene comparison was performed as described in Text S1 in Additional file 3.

### Organ-specific transcript expression pathway analyses

The transcripts for individual organs in neem were mapped to KEGG [43,65-67] pathways using the KAAS server. The KO-assigned transcripts were divided into low and high expression categories. A heat-map of these across the four organs was computed using R. Analyses using KEGG were also carried out for other species, namely *A. thaliana*, *O. sativa*, *V. vinifera* and *C. sinensis*, focusing on the enzymes mapping to the azadirachtin-A biosynthesis pathway. The differential transcript expression level indices of these enzymes in neem relative to other species were compared. The gene structures for these enzymes, and the structures of the top and bottom ten transcripts ranked according to their expression levels were compared across all species.

KEGG annotations obtained for transcripts from all organs were parsed to retain only the un-annotated ones. PASA assemblies, establishing correspondence between each organ's transcripts and common genomic loci were obtained for the same. The top 300 highly expressed un-annotated transcripts for each organ were chosen, filtered for redundancies, to yield a total of 888 unique assemblies. A heat-map was plotted using the log_2_ (FPKM) values of these transcripts using R.

### Accession number

Genome and transcriptome sequence data described in this report are submitted to the NCBI Short Read Archive database [SRA 053330].

## Competing interests

Authors declare no competing interests.

## Authors contribution

BP conceived, planned, designed and directed the project; NMK and SP designed the analytical workflow and guided the process; NMK, SP, PJ, PG, RC, and SV analyzed the data, DSA, AKH and BK produced the Solexa Illumina sequencing data. LV, JN produced the capillary sequencing data; NV, KD and KR produced and helped in the generation of pyro-sequencing data, BK and JN performed the neem genomic DNA cloning experiment; NMK, SP and BP wrote the manuscript. All authors read and approved the final manuscript.

## Supplementary Material

Additional file 1 **Table S1.** Description of sequencing read libraries used for genome and transcriptome assembly. **Table S2.** Classification of repeats in the neem genome. **Table S3.** Transcript annotations from similarity-based analyses. **Table S4.** Mapping neem RNA-Seq reads to other plant genomes. **Table S5.** Transcript mapping statistics using PASA. **Table S6.** Gene ontology based functional categorization of annotated transcripts. **Table S7.** Expression levels of genes associated with metabolism in neem compared to other plant species. Click here for file

Additional file 2 **Figure S1.** kMer frequency curve. **Figure S2.** Frequency histograms for symmetric 4-mers. **Figure S3.** Genome scaffold mapping between neem and other species. **Figure S4.**Gene ontology based functional categorization of annotated transcripts. **Figure S5.** Expression density profiles of neem organ transcriptomes. **Figure S6.** Gene prediction statistics. **Figure S7.** GC-correlated expression density profiles of neem organ transcriptomes. **Figure S8.**AT and GC density profiles in genomes and transcriptomes of neem and other plant species. Click here for file

Additional file 3 **Text S1.** Supplementary methods. Click here for file

Additional file 4 Gene sequences from different species used to perform phylogenetic analysis. Click here for file

Additional file 5 Phylogenetic file generated using rbcL and rbcS genes of 24-plant species. Click here for file

Additional file 6 **Phylogenetic file generated using rbcL gene of *Meliaceae *family members.** Click here for file
