# Supplementary material for: A draft of the genome and four transcriptomes of a medicinal and pesticidal angiosperm Azadirachta indica
Source: BMC Genomics. 2012 Sep 9;13:464. doi: 10.1186/1471-2164-13-464 (PMC3507787; doi:10.1186/1471-2164-13-464)
Supplement: Additional file 1 — Table S1. Description of sequencing read libraries used for genome and transcriptome assembly. Table S2. Classification of repeats in the neem genome. Table S3. Transcript annotations from similarity-based analyses. Table S4. Mapping neem RNA-Seq reads to other plant genomes. Table S5. Transcript mapping statistics using PASA. Table S6. Gene ontology based functional categorization of annotated transcripts. Table S7. Expression levels of genes associated with metabolism in neem compared to other plant species. [file 1471-2164-13-464-S1.pdf]

Table S1

Description of sequencing read libraries used for genome and transcriptome assembly

Genome/ Transcriptome	Chemistry and Platform	Library	Genome coverage
A Genome	Sequencing by synthesis (Solexa/Illumina GAIIx)	76bp, 150 & 350 bp short insert paired end	50X
		36bp, 1.5 kb long insert mate pair	13X
		36bp, 3 kb long insert mate pair	14X
		36bp, 10 kb long insert mate pair	37X
	Capillary Sanger sequencing (Applied Biosystem's 3500)	400-750 bp with either forward or reverse primer	0.01X
	PyroSequencing (IonTorrent Personal-Genome Machine)	100 bp single end	0.5X
B Transcriptomes	Sequencing by synthesis (Solexa/Illumina GAIIx)	72bp short insert paired end, 150bp final libray size	

Table S2

Classification of repeats in the neem genome.

The repeats found using RepeatMasker, LTR Finder, Transposon PSI and MITE-hunter, were classified with respect to type of repeat, number and span of each repeat and summarized as percent occupied in the sequenced genome assembly

**Total number of repeat elements**	**Repeat categories**	**Repeat sub-catagory**	**no. of elements**	**content (bp)**	**% sequenced genome**
	**Retrotransposons**		**142666**	**13543371**	**3.72**
		LTR retrotransposons	***136837***	***12670662***	***3.48***
		Gypsy	64537	6614251	1.82
		Copia	59698	6063363	1.67
		Caulimovirus	2154	373115	0.1
		Unclassified	10447	1596072	0.44
		Non-LTR retrotransposons	***5829***	***907094***	***0.25***
		LINEs	5757	903022	0.25
		SINEs	39	2866	0
		Unclassified	33	6037	0
	**DNA Transposons**		**15661**	**2721422**	**0.75**
		Terminal Inverted Repeats (TIRs)	***13027***	***2424775***	***0.67***
		CACTA	743	188074	0.05
		Mutator	7218	1310373	0.36
		hAT	4501	836514	0.23
		PIF/Harbinger	564	89844	0.02
		Chapaev	299	29588	0.01
		En-Spm	2106	347739	0.1
		P	229	49661	0.01
	MITEs		5608	597728	0.16

	Helitrons		1515	326650	0.09
	RNA Repeats		17591	671627	0.18
	Unclassified Sequences		187867	29024805	7.97
**Total interspersed elements**			**348332**	**40790504**	**11.21**
**Total tandem repeat elements**			**249047**	**13045902**	**3.58**
		Low complexity	212508	11544515	3.17
		Simple repeats	1072	171283	0.05
		Satellites	35407	1380226	0.38
**Percent of unique repeat elements**			**557716**	**47427034**	**13.03**

Table S3

Transcript annotations from similarity-based analyses.

The serial annotations of transcripts from all organs using MegaBlast against the non-redundant nucleotide database, BlastX against the non-redundant protein database, and MegaBlast against the RefSeqRNA, EST (Expressed Sequence Tag) databases and finally processing them through the AutoFACT pipeline which performed Blast against uniref90, uniref100, KEGG and cog databases are summarized at each step.

**Annotations for each organ**	**Root**	**Leaf**	**Stem**	**Flower**
MegaBlast+	16177	15410	19350	17044
MegaBlast-	11739	11959	15168	14179
MegaBlast- BlastX+	11155	9848	12638	13309
MegaBlast- BlastX-	584	2111	2530	870
MegaBlast- BlastX- RefSeqRNA+	0	0	0	0
MegaBlast- BlastX- RefSeqRNA-	584	2111	2530	870
MegaBlast- BlastX- RefSeqRNA- EST+	55	325	396	91
MegaBlast- BlastX- RefSeqRNA- EST-	529	1786	2134	779
MegaBlast- BlastX- RefSeqRNA- EST- TSA+	0	0	0	0
MegaBlast- BlastX- RefSeqRNA- EST- TSA-	529	1786	2134	779
MegaBlast- BlastX- RefSeqRNA- EST- Autofact+	0	0	288	154
MegaBlast- BlastX- RefSeqRNA- EST- Autofact-	529	1786	1846	625

Table S4
Mapping *A. indica* RNA-Seq reads to other plant genomes
The Neem RNA-Seq reads were aligned with assembled genomes from various other plant species, using TopHat. The resulting alignment (bam file) was assembled into a parsimonius set of transcripts using CuffLinks, and the numbers of transcripts thus obtained are summarized.

Plant species	Number of non-overlapping genes
Citrus clementina	22043
Citrus seninsis	21501
Glycine max	2244
Sorghum bicolor	275
Ricinus cummunis	992
Zea mays	544
Carica papaya	1558
Theobroma cacao	4778
Manihot esculenta	4955
Cucumis sativus	1033
Prunus persica	2250
Solanum lycopersicum	1177

Table S5

Transcript mapping statistics using PASA.

The gene structures predicted using PASA based on GMAP mappability in each organ (root, leaf, stem and flower) are summarized here.

	PASA Assembly Statistics			
	**Root**	**Leaf**	**Stem**	**Flower**
**Total Transcripts**	27916	27369	34518	31223
**Total Scaffolds**	9714			
**Mapped Transcripts**	27890	27324	34493	30565
**Mapped Scaffolds**	1610	1647	1760	1685

Table S6

Gene ontology based functional categorization of annotated transcripts.

Blastx (Expect value cut off of 0.001) hits for transcripts of each organ (root, leaf, stem and flower) were formatted as xml, and subject to Blast2GO mappings. The GO annotated transcripts are summarized here. The neem-specific transcripts are identified by comparisons to GO-annotations of other plant species.

**Organ**	**GO Annotated**	**Neem specific**
Root	18056	679
Flower	23983	197
Stem	26786	21
Leaf	21666	482

Table S7 **Expression levels of genes associated with metabolism in neem compared to other plant species.**

The transcriptome assemblies of neem, *Arabidopsis thaliana*, *Oryza sativa* and *Vitis vinifera* were subjected to pathway analyses using KEGG's KAAS pipeline. Expression level ratios were calculated between metabolism pathway related genes expressed in neem leaf to those expressed in leaves of *A.thaliana*, *O.sativa*, and berries of *V.vinifera*, after normalizing the expression levels of all genes to that of a housekeeping control gene, elongation factor 1-alpha (EF1A).

**Over- and Under-expressed Genes in Neem w.r.t Other Plants**	**Ratio of gene expression levels in Neem to that in:**		
	Vitis vinifera	***Oryza sativa***	***Arabidopsis thaliana***
E1.14.17.4; aminocyclopropanecarboxylate oxidase [EC:1.14.17.4]	4.09	11.67	2.08
E3.2.1.20, malZ; alpha-glucosidase [EC:3.2.1.20]	3	52.47	57.79
chlP, bchP; geranylgeranyl reductase [EC:1.3.1.83]	40.44	0.31	0.2
LHCA1; light-harvesting complex I chlorophyll a/b binding protein 1	27	0.26	0.21
LHCB6; light-harvesting complex II chlorophyll a/b binding protein 6	23.82	0.15	0.24
LHCA4; light-harvesting complex I chlorophyll a/b binding protein 4	22.55	0.11	0.24
hemA; glutamyl-tRNA reductase [EC:1.2.1.70]	29.78	0.21	0.26
psaK; photosystem I subunit X	15.59	0.1	0.27
LHCB5; light-harvesting complex II chlorophyll a/b binding protein 5	21.11	0.24	0.32
hemL; glutamate-1-semialdehyde 2,1-aminomutase [EC:5.4.3.8]	11.24	0.24	0.33
psbW; photosystem II PsbW protein	14.62	0.13	0.35
psaF; photosystem I subunit III	24.38	0.28	0.4
LHCA2; light-harvesting complex I chlorophyll a/b binding protein 2	21.01	0.26	0.41
psaN; photosystem I subunit PsaN	22.76	0.24	0.42
E1.14.13.81, acsF, chlE; magnesium-protoporphyrin IX monomethyl ester (oxidative) cyclase [EC:1.14.13.81]	35.69	0.3	0.45
petE; plastocyanin	34.32	0.29	0.48

LHCA3; light-harvesting complex I chlorophyll a/b binding protein 3	22.46	0.35	0.54
petC; cytochrome b6-f complex iron-sulfur subunit [EC:1.10.9.1]	24.71	0.41	0.55
PAPSS; 3'-phosphoadenosine 5'-phosphosulfate synthase [EC:2.7.7.4 2.7.1.25]	11.06	0.48	0.61
psaL; photosystem I subunit XI	42.26	0.47	0.62
rpe, RPE; ribulose-phosphate 3-epimerase [EC:5.1.3.1]	33.06	0.18	0.68
psbO; photosystem II oxygen-evolving enhancer protein 1	25.92	0.25	0.7
rpiA; ribose 5-phosphate isomerase A [EC:5.3.1.6]	13.23	0.3	0.72
chlH, bchH; magnesium chelatase subunit H [EC:6.6.1.1]	68.71	0.67	0.73
ATPF1D, atpH; F-type H+-transporting ATPase subunit delta [EC:3.6.3.14]	48.34	0.46	0.74
psaG; photosystem I subunit V	18.69	0.18	0.76
FBP, fbp; fructose-1,6-bisphosphatase I [EC:3.1.3.11]	41.94	0.53	0.76
ATPeF1O, ATP5O; F-type H+-transporting ATPase oligomycin sensitivity conferral protein [EC:3.6.3.14]	11.91	1.01	0.77
dxr; 1-deoxy-D-xylulose-5-phosphate reductoisomerase [EC:1.1.1.267]	10.62	0.49	0.8
psbY; photosystem II PsbY protein	17.31	0.28	0.83
FAD8, desB; omega-3 fatty acid desaturase (delta-15 desaturase) [EC:1.14.19.-]	15.37	0.41	0.85
psbR; photosystem II 10kDa protein	25.91	0.16	0.93
gcvT, AMT; aminomethyltransferase [EC:2.1.2.10]	64.35	0.51	0.97
E1.17.7.1, gcpE, ispG; (E)-4-hydroxy-3-methylbut-2-enyl-diphosphate synthase [EC:1.17.7.1]	17.51	0.66	0.98
cysK; cysteine synthase A [EC:2.5.1.47]	11.19	0.31	1.04
E2.2.1.6L, ilvB, ilvG, ilvI; acetolactate synthase I/II/III large subunit [EC:2.2.1.6]	21.94	1.7	1.06
NOL, NYC1; chlorophyll(ide) b reductase [EC:1.1.1.294]	11.87	1.69	1.09
MDH2; malate dehydrogenase [EC:1.1.1.37]	16.54	0.42	1.13
psaH; photosystem I subunit VI	19.88	0.2	1.15
E2.7.1.19, prkB; phosphoribulokinase [EC:2.7.1.19]	62.62	0.27	1.19

ATPF0B, atpF; F-type H+-transporting ATPase subunit b [EC:3.6.3.14]	23.94	0.42	1.23
psaE; photosystem I subunit IV	22.55	0.24	1.26
fabF; 3-oxoacyl-[acyl-carrier-protein] synthase II [EC:2.3.1.179]	11.59	1.89	1.26
glyA, SHMT; glycine hydroxymethyltransferase [EC:2.1.2.1]	21.52	0.63	1.27
E3.1.3.37; sedoheptulose-bisphosphatase [EC:3.1.3.37]	80.76	0.43	1.31
glgC; glucose-1-phosphate adenylyltransferase [EC:2.7.7.27]	13.87	2.32	1.33
E4.4.1.5, GLO1, gloA; lactoylglutathione lyase [EC:4.4.1.5]	16.15	1.89	1.45
E1.1.1.82; malate dehydrogenase (NADP+) [EC:1.1.1.82]	51.94	1.65	1.5
gltS; glutamate synthase (ferredoxin) [EC:1.4.7.1]	31.31	0.59	1.52
E3.13.1.1, sqd1, sqdb; UDP-sulfoquinovose synthase [EC:3.13.1.1]	16.22	1.61	1.6
aroDE, DHQ-SDH; 3-dehydroquinate dehydratase / shikimate dehydrogenase [EC:4.2.1.10 1.1.1.25]	16.82	1.35	1.61
E2.4.1.18, glgB; 1,4-alpha-glucan branching enzyme [EC:2.4.1.18]	10.89	5.07	1.61
petH; ferredoxin--NADP+ reductase [EC:1.18.1.2]	73.57	1.57	1.64
GPI, pgi; glucose-6-phosphate isomerase [EC:5.3.1.9]	10.95	0.82	1.83
GAPA; glyceraldehyde-3-phosphate dehydrogenase (NADP+) (phosphorylating) [EC:1.2.1.13]	109.1	0.91	1.86
ispE; 4-diphosphocytidyl-2-C-methyl-D-erythritol kinase [EC:2.7.1.148]	10.3	2.14	1.88
GPT, ALT; alanine transaminase [EC:2.6.1.2]	20.3	0.87	1.88
cynT, can; carbonic anhydrase [EC:4.2.1.1]	173.09	0.31	1.99
hemB, ALAD; porphobilinogen synthase [EC:4.2.1.24]	16.91	0.55	2.09
petF; ferredoxin	36.14	0.44	2.44
AGXT; alanine-glyoxylate transaminase / serine-glyoxylate transaminase / serine-pyruvate transaminase [EC:2.6.1.44 2.6.1.45 2.6.1.51]	186.79	1.57	2.84
psbS; photosystem II 22kDa protein	72.97	0.77	2.89
NIT2; omega-amidase [EC:3.5.1.3]	13.92	1.62	2.9
pdxS, pdx1; pyridoxine biosynthesis protein [EC:4.-.-.-]	10.56	2.98	2.96
ispF; 2-C-methyl-D-erythritol 2,4-cyclodiphosphate synthase [EC:4.6.1.12]	33.91	3.32	3.01

rbcS; ribulose-bisphosphate carboxylase small chain [EC:4.1.1.39]	104.68	0.26	3.14
E2.1.1.104; caffeoyl-CoA O-methyltransferase [EC:2.1.1.104]	14.35	5.19	3.32
hemH, FECH; ferrochelatase [EC:4.99.1.1]	38.22	2.29	3.4
ACAC; acetyl-CoA carboxylase / biotin carboxylase [EC:6.4.1.2 6.3.4.14]	18.18	3.56	3.43
SPS, sds; all-trans-nonaprenyl-diphosphate synthase [EC:2.5.1.84 2.5.1.85]	48.44	1.1	3.66
ALDO, fbaB; fructose-bisphosphate aldolase, class I [EC:4.1.2.13]	25.55	1.02	3.87
SQLE, ERG1; squalene monooxygenase [EC:1.14.13.132]	27.77	2.04	4.07
E2.3.1.30, cysE; serine O-acetyltransferase [EC:2.3.1.30]	12.6	6.09	4.51
E1.2.1.3; aldehyde dehydrogenase (NAD+) [EC:1.2.1.3]	15.71	9.01	6.03
AGXT2; alanine-glyoxylate transaminase / (R)-3-amino-2-methylpropionate-pyruvate transaminase [EC:2.6.1.44 2.6.1.40]	14.91	1.3	6.47
E1.2.1.9, gapN; glyceraldehyde-3-phosphate dehydrogenase (NADP) [EC:1.2.1.9]	60.15	1.17	6.67
GLDC, gcvP; glycine dehydrogenase [EC:1.4.4.2]	190.33	2.47	7.52
E6.2.1.12; 4-coumarate--CoA ligase [EC:6.2.1.12]	22.86	2.42	8.24
E2.4.1.241; digalactosyldiacylglycerol synthase [EC:2.4.1.241]	17.77	2.54	10.12
E5.5.1.6; chalcone isomerase [EC:5.5.1.6]	19.4	2.04	10.22
CYP73A; trans-cinnamate 4-monooxygenase [EC:1.14.13.11]	20.26	8.09	10.55
NIT4; beta-cyano-L-alanine hydratase/nitrilase [EC:3.5.5.1 3.5.5.4]	11.25	1.18	11.11
E2.3.1.74, bcsA; chalcone synthase [EC:2.3.1.74]	11.5	5.19	16.33
GGPS; geranylgeranyl diphosphate synthase, type II [EC:2.5.1.1 2.5.1.10 2.5.1.29]	156.57	31.94	18.67
HAO; (S)-2-hydroxy-acid oxidase [EC:1.1.3.15]	97.46	1.46	89.43
psaO; photosystem I subunit PsaO	7.75	0.05	0.09
psbQ; photosystem II oxygen-evolving enhancer protein 3	9.89	0.09	0.38
rbcL; ribulose-bisphosphate carboxylase large chain [EC:4.1.1.39]	4.56	0.1	0.08
