# Supplementary material for: A draft of the genome and four transcriptomes of a medicinal and pesticidal angiosperm Azadirachta indica
Source: BMC Genomics. 2012 Sep 9;13:464. doi: 10.1186/1471-2164-13-464 (PMC3507787; doi:10.1186/1471-2164-13-464)

Figure S1
kMer frequency curve.
The numbers of kMers are plotted against the frequency at which the kMer occurs. The size of each kMer is 31. The statistics were obtained during the course of genome assembly using SOAP *de novo* while using the 76 bp short insert read library with the kMer frequency cutoff option (-d) turned off. kMers with a frequency of 1 were omitted in this graph.

Figure S1

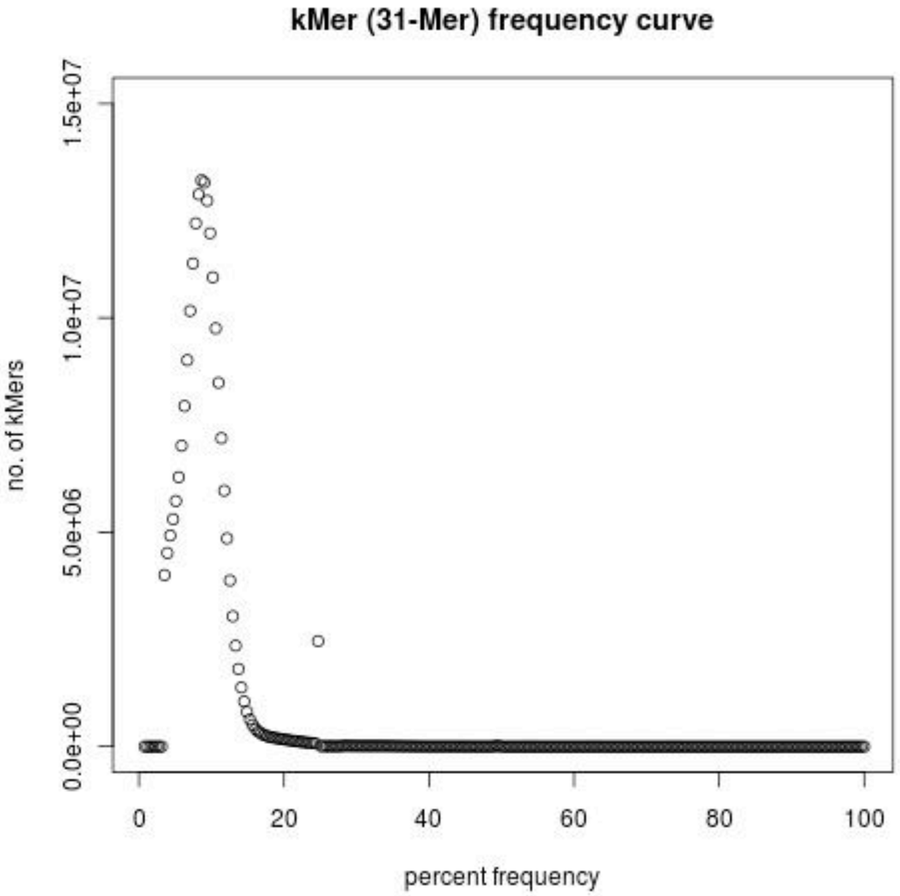
kMer (31-Mer) frequency curve
no. of kMers
0.0e+00
5.0e+06
1.0e+07
1.5e+07
0
20
40
60
80
100
percent frequency

Figure S2
Frequency histograms for symmetric 4-mers.
The neem scaffolds and chromosomes in *Arabidopsis thaliana* were divided into overlapping 4-mers. The frequency ratio of each 4-mer with its reverse complementary counterpart was estimated across the genome and plotted as histograms for the two species.

Figure S2

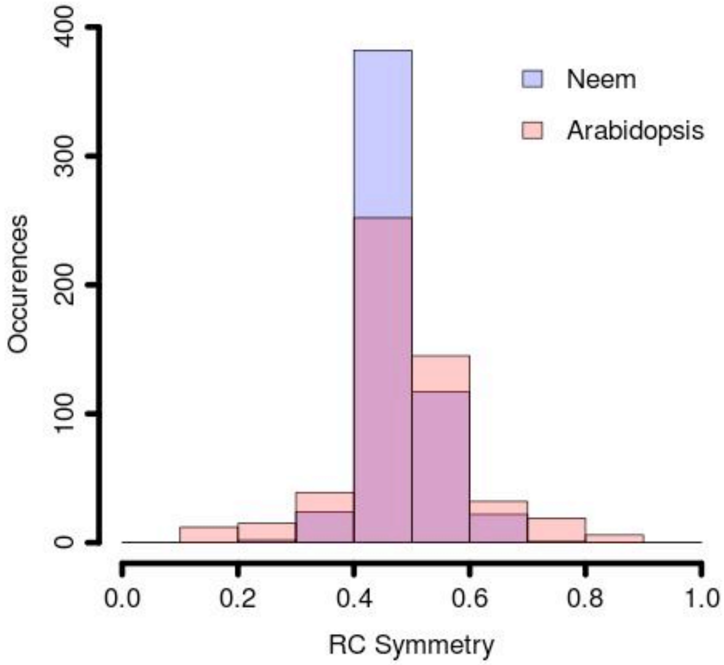
Neem
Arabidopsis
Occurences
0
100
200
300
400
0.0
0.2
0.4
0.6
0.8
1.0
RC Symmetry

Figure S3

Genome scaffold mapping between neem and other species.

Genome scaffolds of neem were compared against those of *Citrus sinensis (a), Citrus clementine (b), Arabidopsis thaliana (c)*, *Oryza sativa (d)*, *Theobroma cacao (e)*, *Vitis vinifera (f), Ricinus communis (g)* and *Sorghum bicolor (h)*, using TblastX, with an Expect value cutoff of 0. The mapped scaffold pairs were short-listed to contain only those scaffolds with lengths greater than the scaffold N50 of the respective assembly. These scaffolds and the mappings between them are represented here using Circos (yellow indicating the neem scaffolds and blue indicating chromosomes/scaffolds from other species).

Figure S3
a

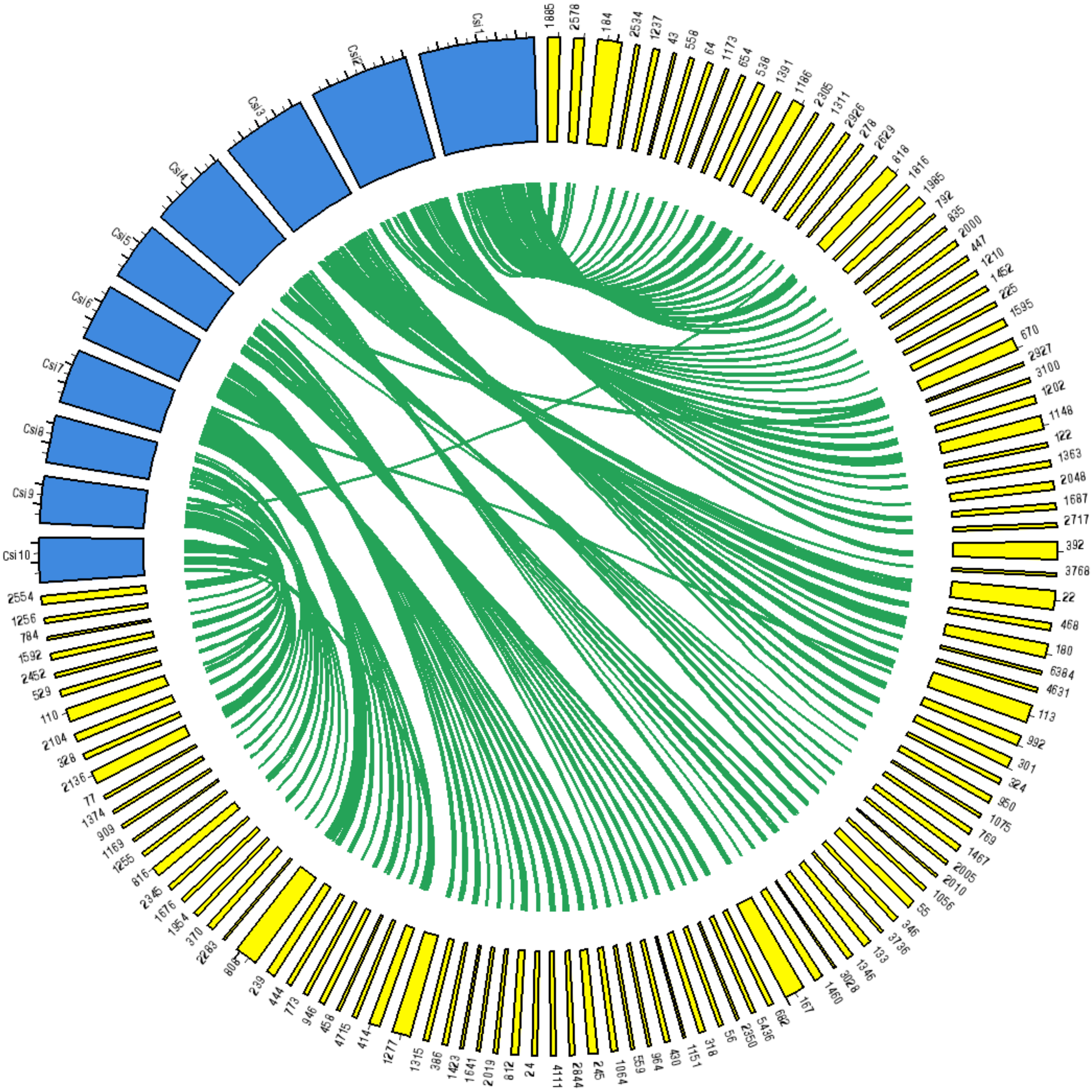
Csi1
Csi2
Csi3
Csi4
Csi5
Csi6
Csi7
Csi8
Csi9
Csi10
1885
2578
184
2534
1237
43
558
64
1173
654
538
1391
1186
2305
1311
2926
278
2629
818
1816
1985
792
835
2000
447
1210
1452
225
1595
670
2927
3100
1202
1148
122
1363
2048
1687
2717
392
3768
22
468
180
6384
4631
113
992
301
324
950
1075
769
1467
2005
2010
1056
55
346
3736
133
1346
3028
1460
167
682
5436
2350
56
318
1151
430
964
559
1064
245
2844
4111
24
812
2019
1641
1423
386
1315
1277
414
4715
458
946
773
444
239
808
2283
370
1954
1676
2345
816
1255
1169
909
1374
77
2136
328
2104
110
529
2452
1592
784
1256
2554

b

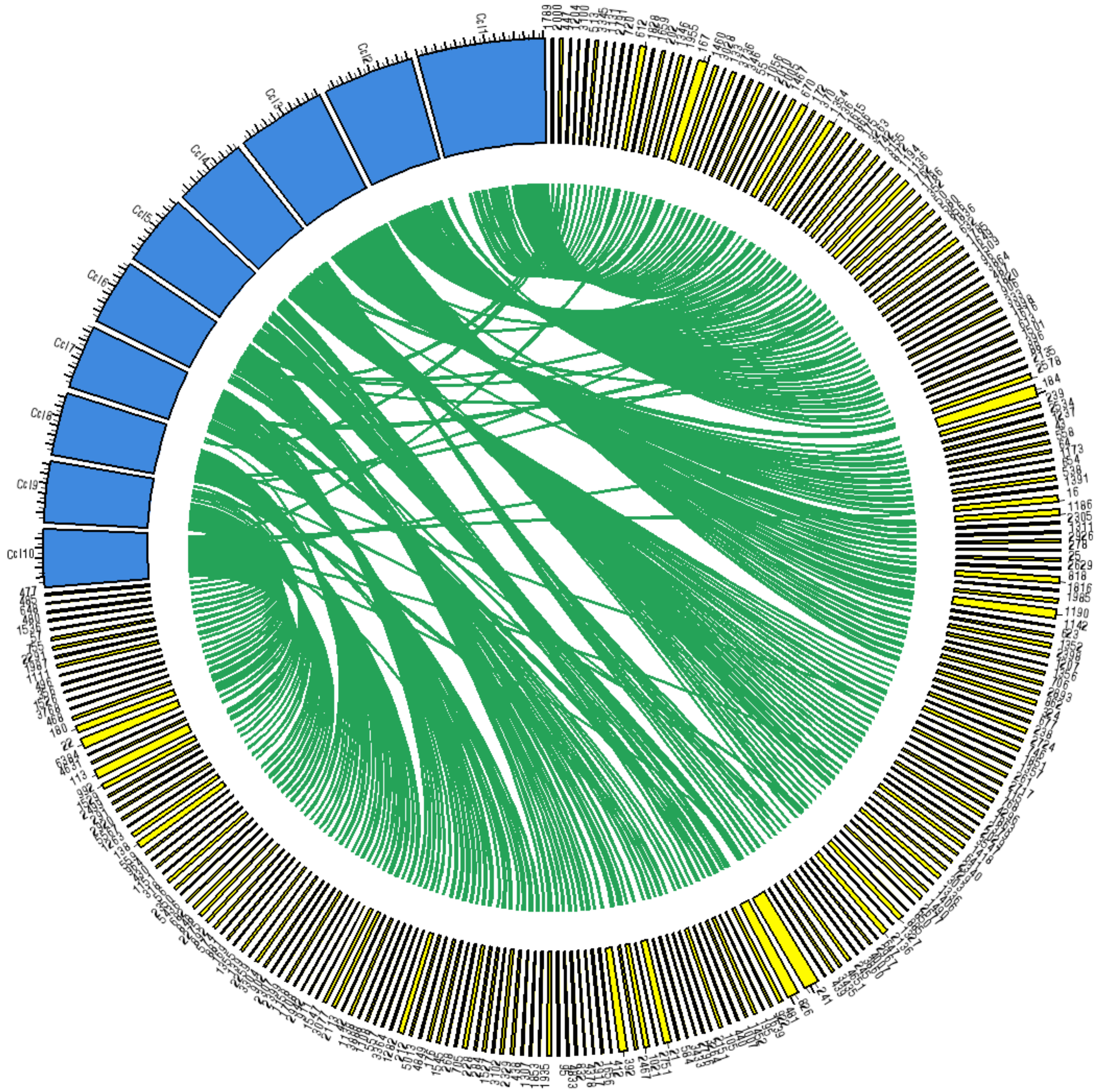

c

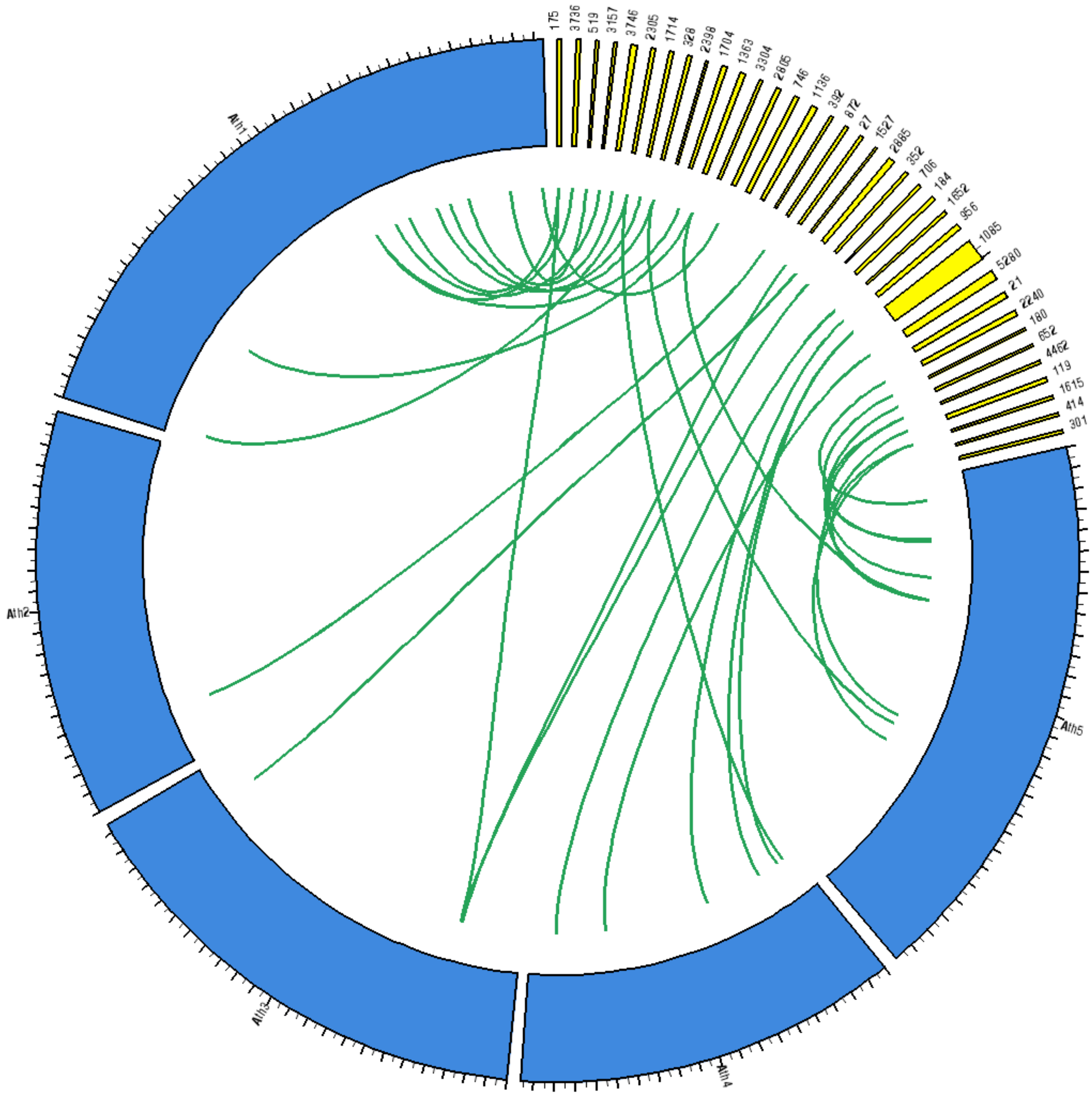

Ath1
Ath2
Ath3
Ath4
Ath5
175
3736
519
3157
3746
2305
1714
328
2398
1704
1363
3304
2805
746
1136
392
872
27
1527
2885
352
706
184
1652
956
1085
5280
21
2240
180
652
4462
119
1615
414
301

d

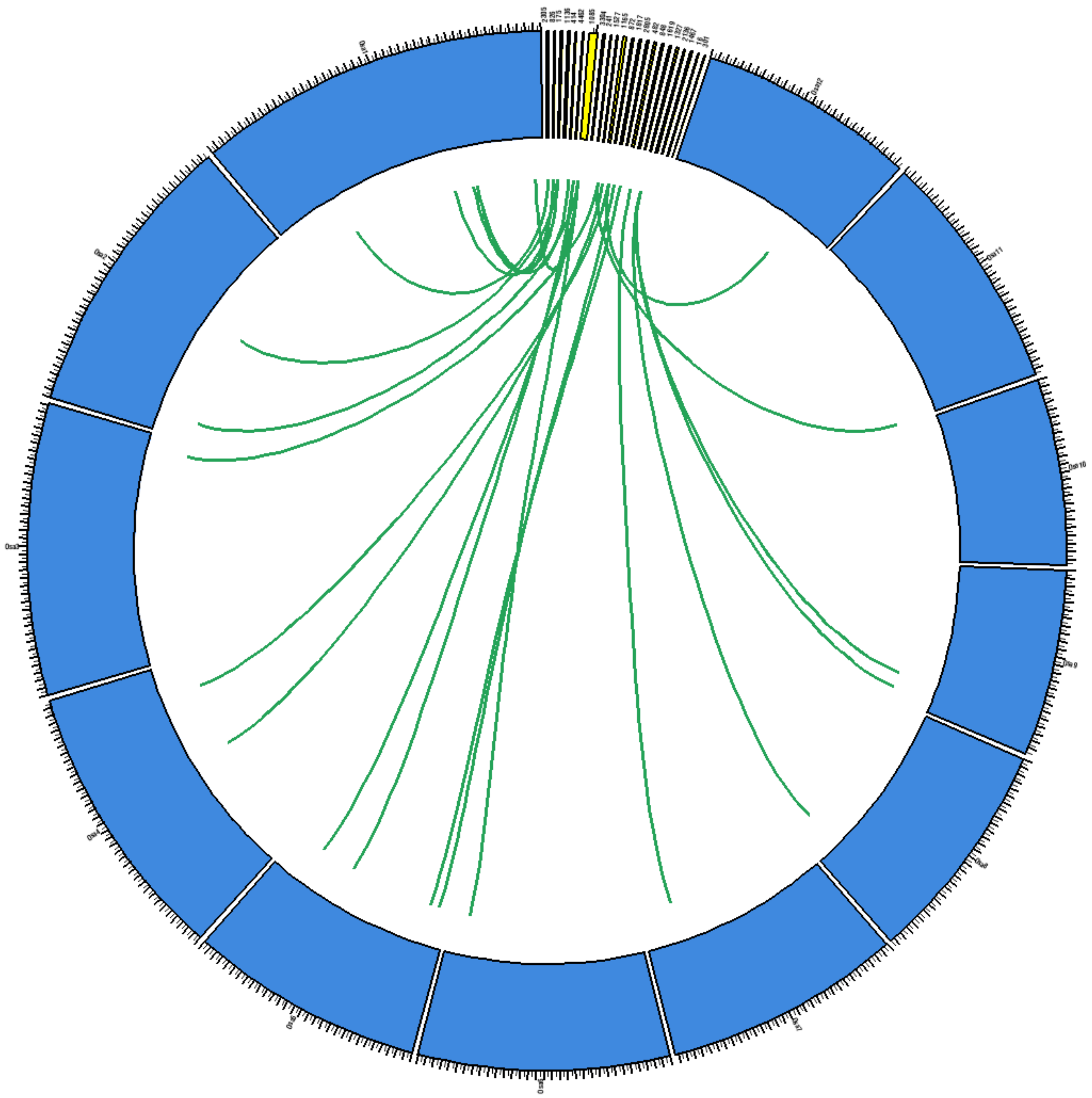

e

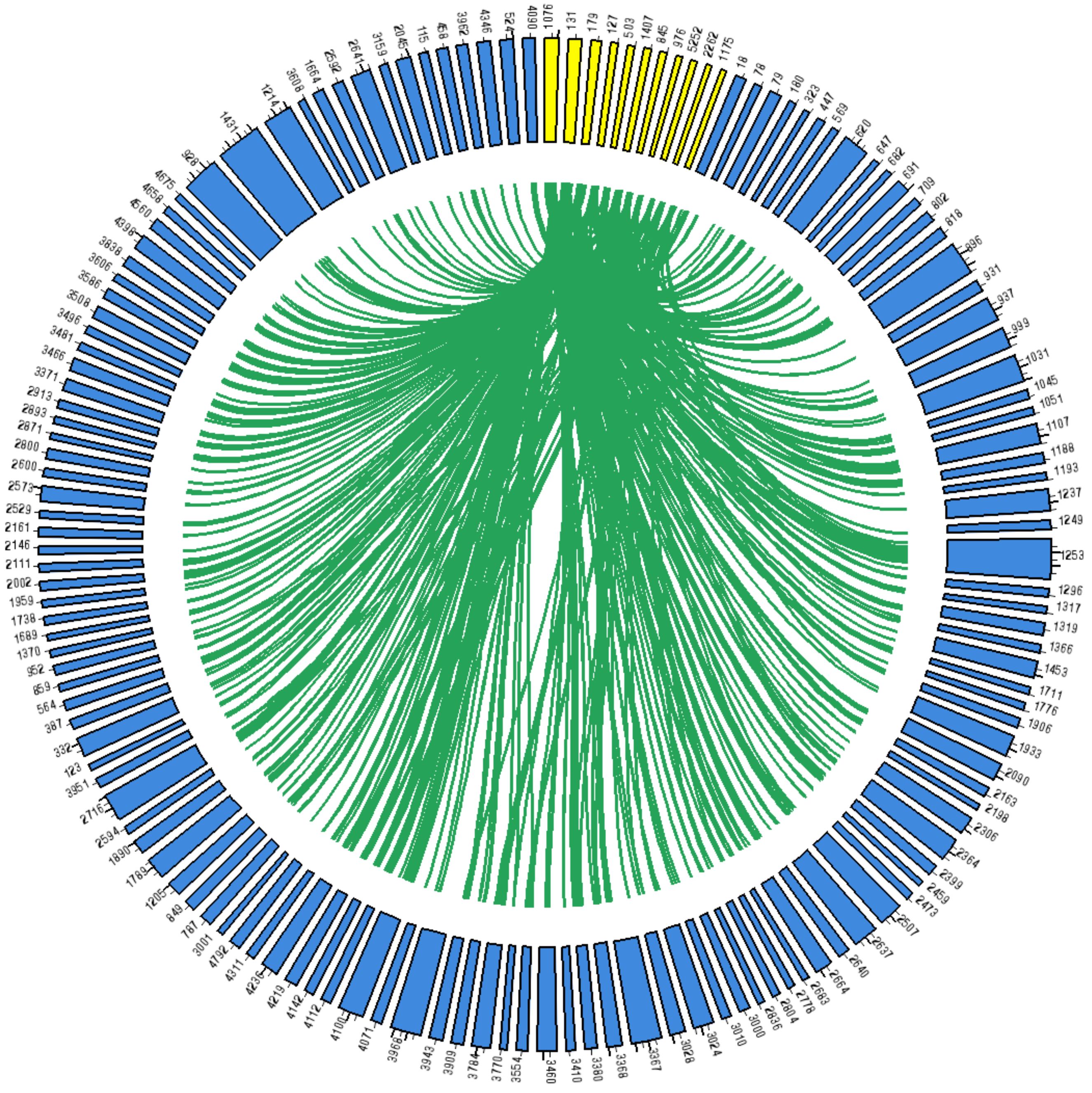
1076
131
179
127
503
1407
845
976
5252
2262
1175
18
78
79
180
323
447
569
620
647
682
691
709
802
818
896
931
937
999
1031
1045
1051
1107
1188
1193
1237
1249
1253
1296
1317
1319
1366
1453
1711
1776
1906
1933
2090
2163
2198
2306
2364
2399
2459
2473
2507
2637
2640
2664
2683
2778
2804
2836
3000
3010
3024
3028
3367
3368
3380
3410
3460
3554
3770
3784
3909
3943
3966
4071
4100
4112
4142
4219
4236
4311
4792
3001
787
849
1205
1789
1890
2594
2716
3951
123
332
387
564
859
952
1370
1689
1738
1959
2002
2111
2146
2161
2529
2573
2600
2800
2871
2893
2913
3371
3466
3481
3496
3508
3586
3606
3838
4398
4560
4658
4675
928
1431
1214
3608
1664
2592
2641
3159
2045
115
458
3962
4346
524
4090

f

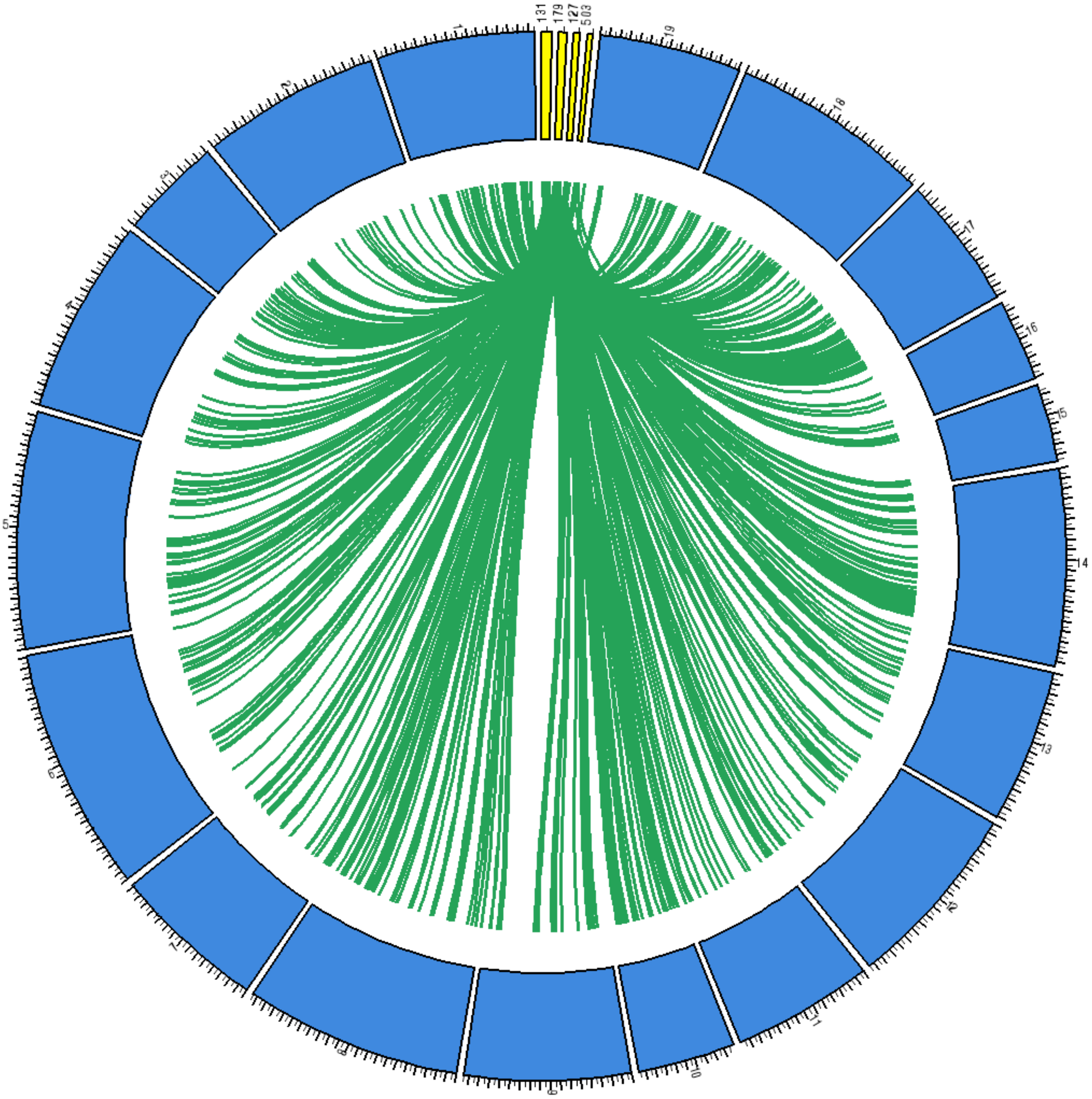

g

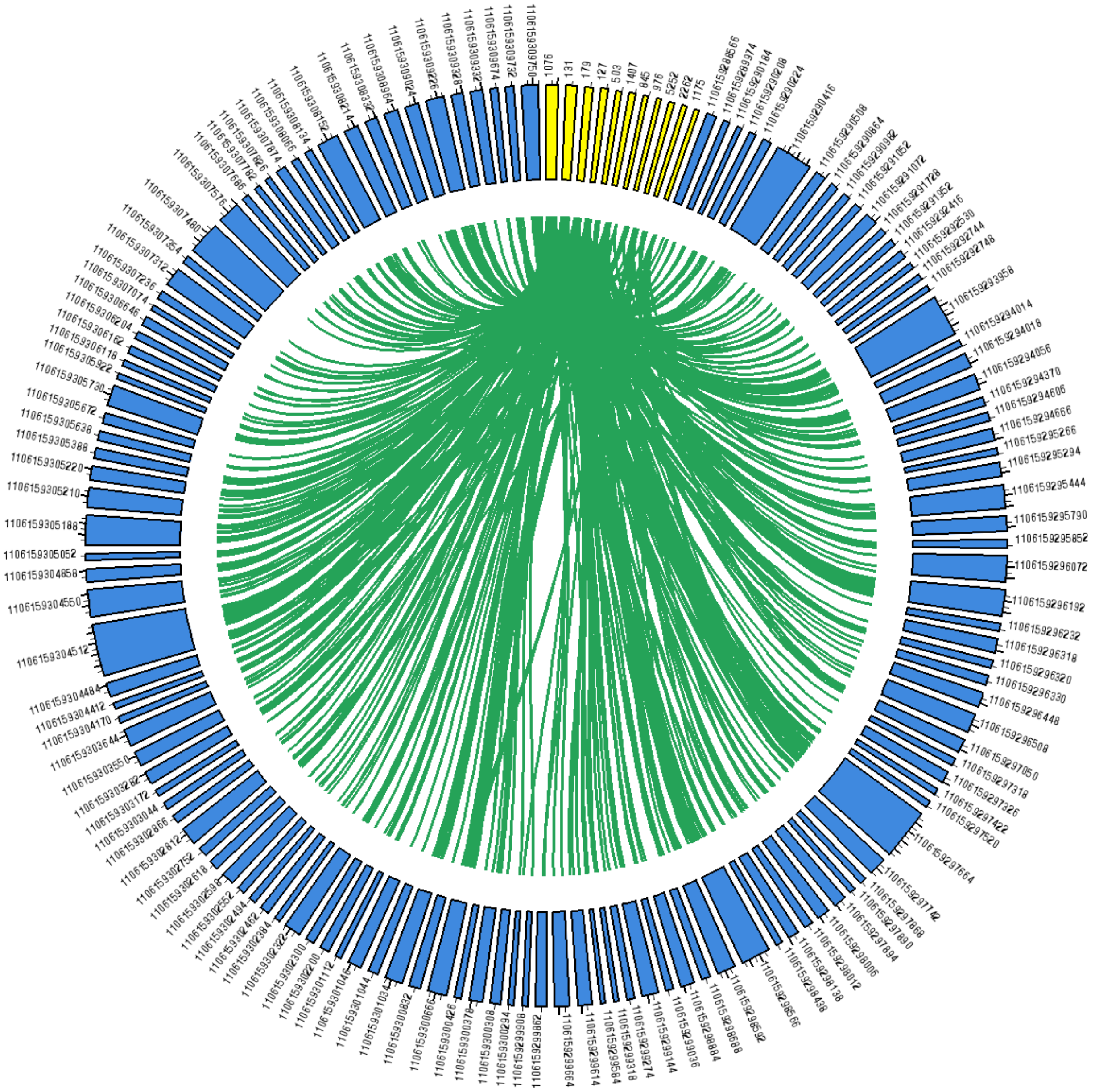

h

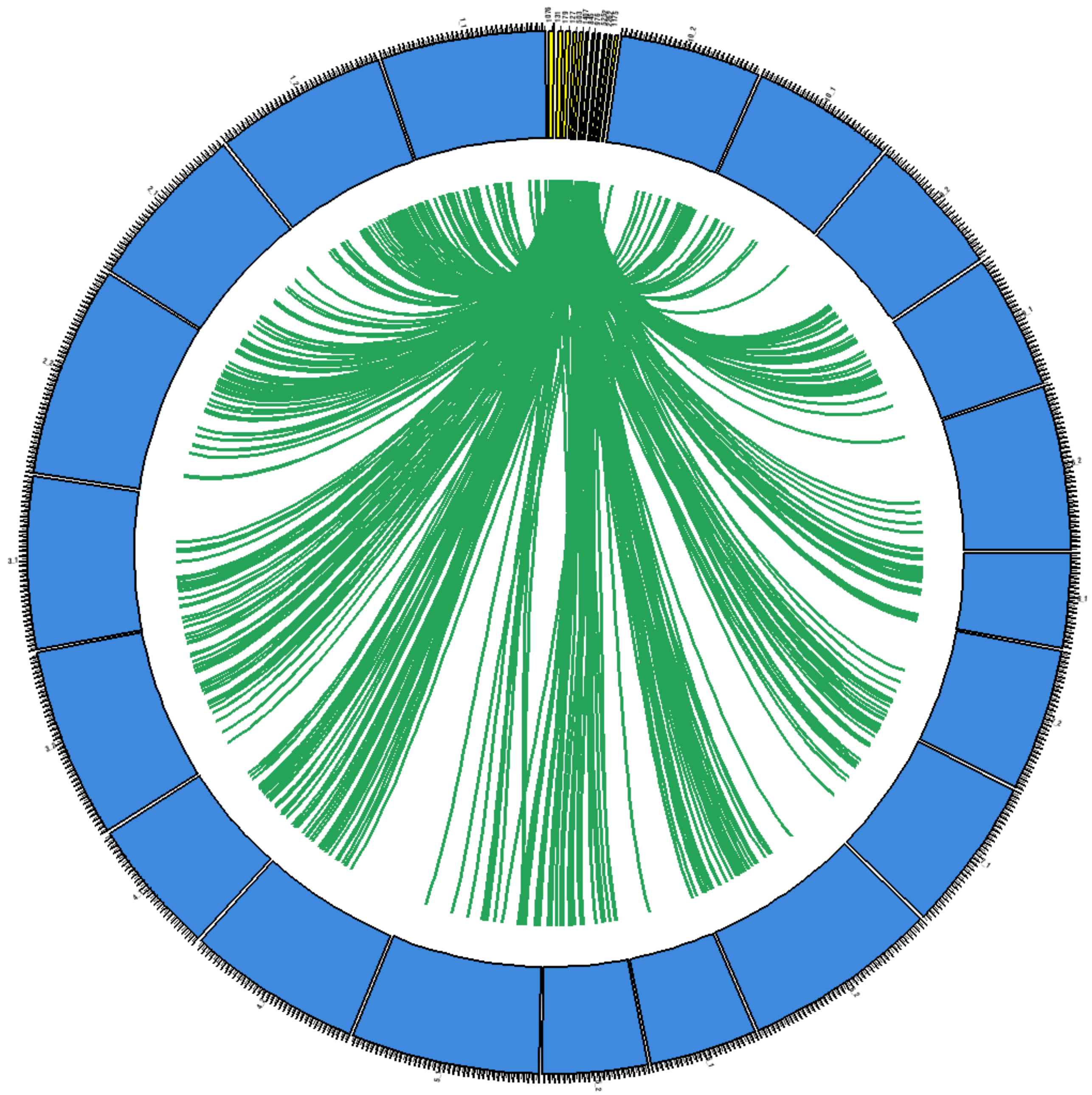

Figure S4

Gene ontology based functional categorization of annotated transcripts. Blastx (Expect value cut off of 0.001) hits for transcripts of each organ (root, leaf, stem and flower) were formatted as xml, and subject to Blast2GO mappings. The GO terms and scores are categorized based on biological processes, molecular function and cellular components. The pie charts depict the categorization at the top and bottom two levels for each organ.

Figure S4

a

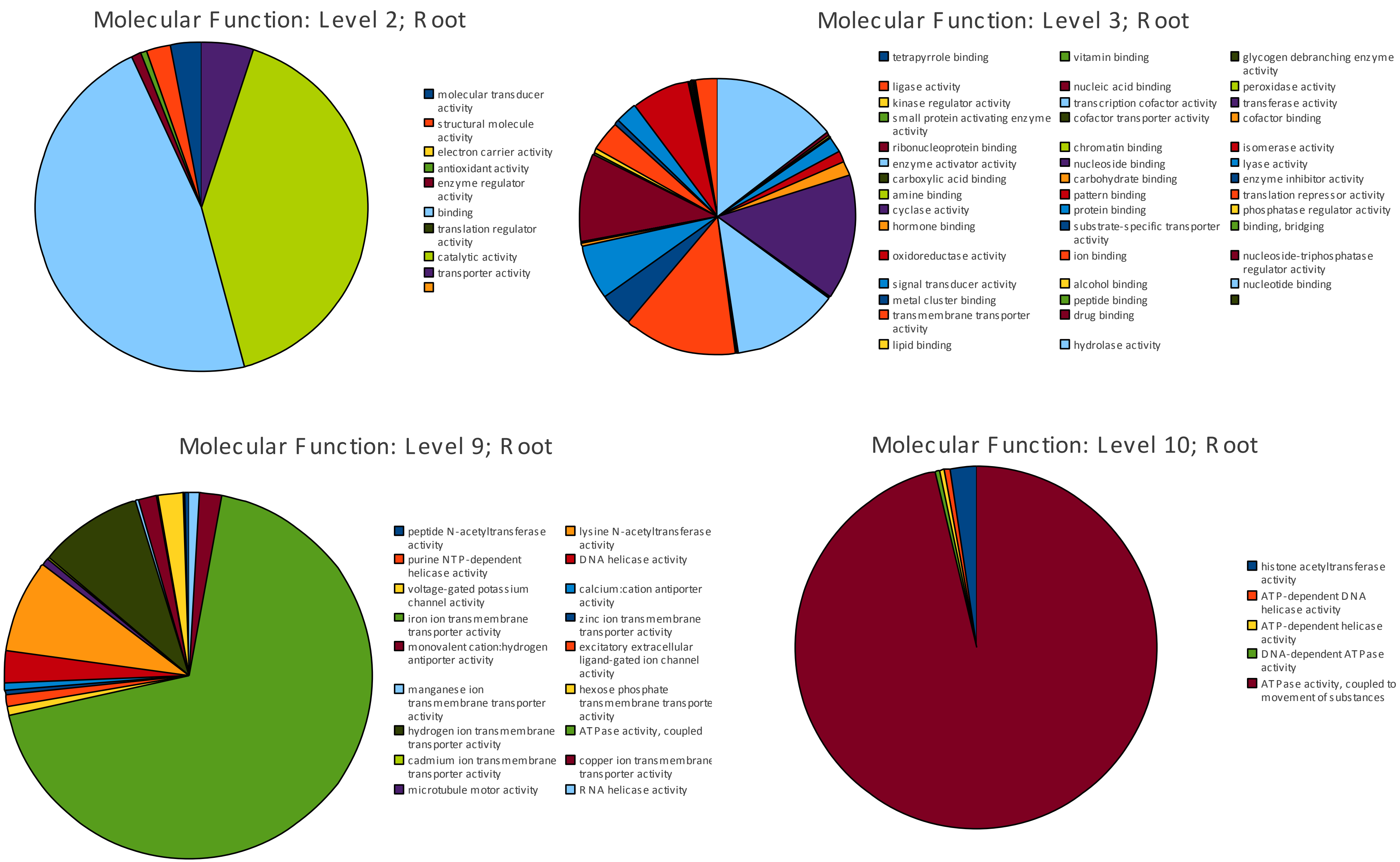
Molecular Function: Level 2; Root
molecular transducer activity
structural molecule activity
electron carrier activity
antioxidant activity
enzyme regulator activity
binding
translation regulator activity
catalytic activity
transporter activity
Molecular Function: Level 3; Root
tetrapyrrole binding
vitamin binding
glycogen debranching enzyme activity
ligase activity
nucleic acid binding
peroxidase activity
kinase regulator activity
transcription cofactor activity
transferase activity
small protein activating enzyme activity
cofactor transporter activity
cofactor binding
ribonucleoprotein binding
chromatin binding
isomerase activity
enzyme activator activity
nucleoside binding
lyase activity
carboxylic acid binding
carbohydrate binding
enzyme inhibitor activity
amine binding
pattern binding
translation repressor activity
cyclase activity
protein binding
phosphatase regulator activity
hormone binding
substrate-specific transporter activity
binding, bridging
oxidoreductase activity
ion binding
nucleoside-triphosphatase regulator activity
signal transducer activity
alcohol binding
nucleotide binding
metal cluster binding
peptide binding
transmembrane transporter activity
drug binding
lipid binding
hydrolase activity
Molecular Function: Level 9; Root
peptide N-acetyltransferase activity
lysine N-acetyltransferase activity
purine NTP-dependent helicase activity
DNA helicase activity
voltage-gated potassium channel activity
calcium:cation antiporter activity
iron ion transmembrane transporter activity
zinc ion transmembrane transporter activity
monovalent cation:hydrogen antiporter activity
excitatory extracellular ligand-gated ion channel activity
manganese ion transmembrane transporter activity
hexose phosphate transmembrane transporte activity
hydrogen ion transmembrane transporter activity
ATPase activity, coupled
cadmium ion transmembrane transporter activity
copper ion transmembrane transporter activity
microtubule motor activity
RNA helicase activity
Molecular Function: Level 10; Root
histone acetyltransferase activity
ATP-dependent DNA helicase activity
ATP-dependent helicase activity
DNA-dependent ATPase activity
ATPase activity, coupled to movement of substances

Biological Process: Level 2; Root

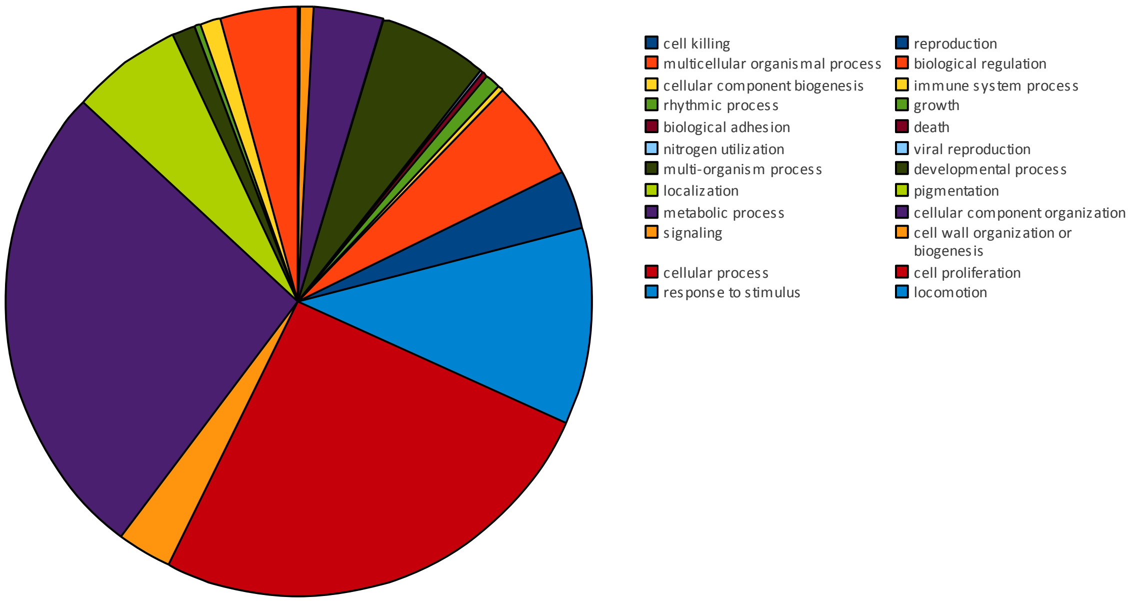

Biological Process: Level 3; Root

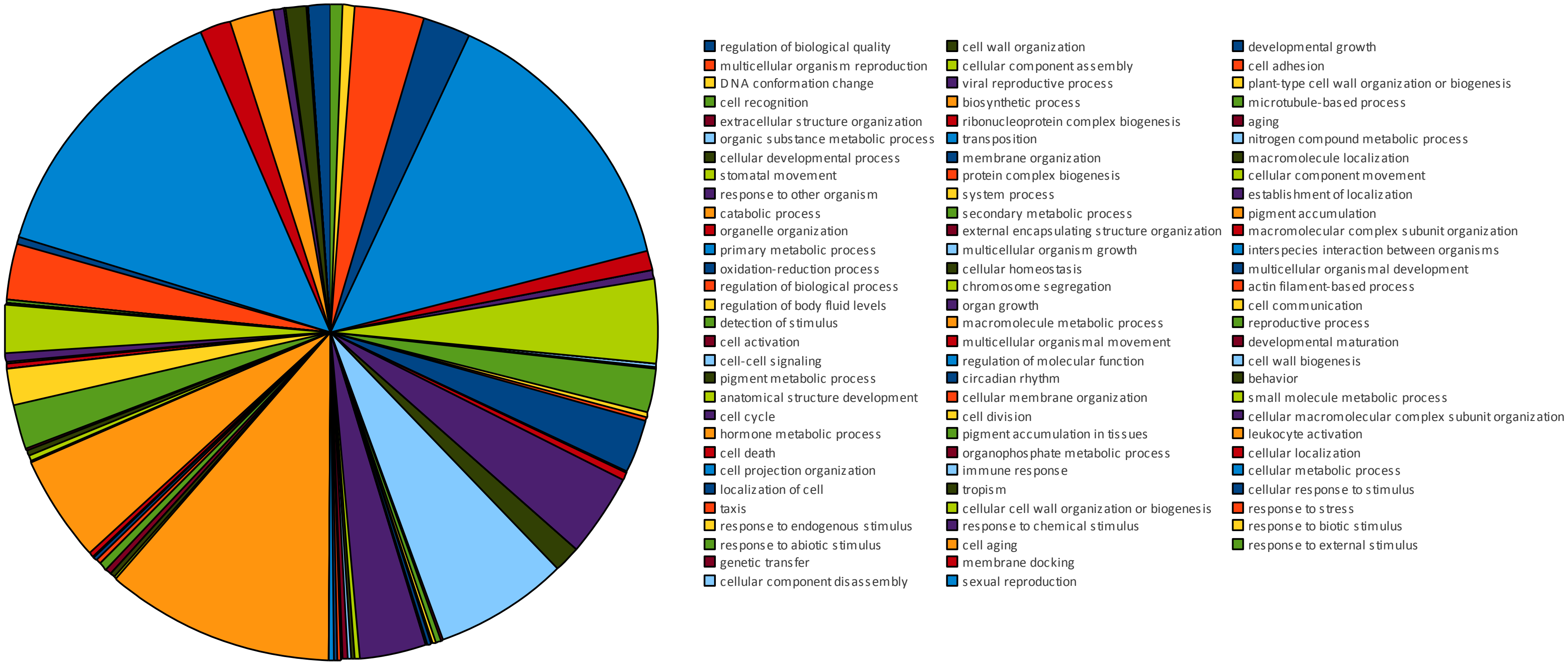

Biological Process: Level 9; Root

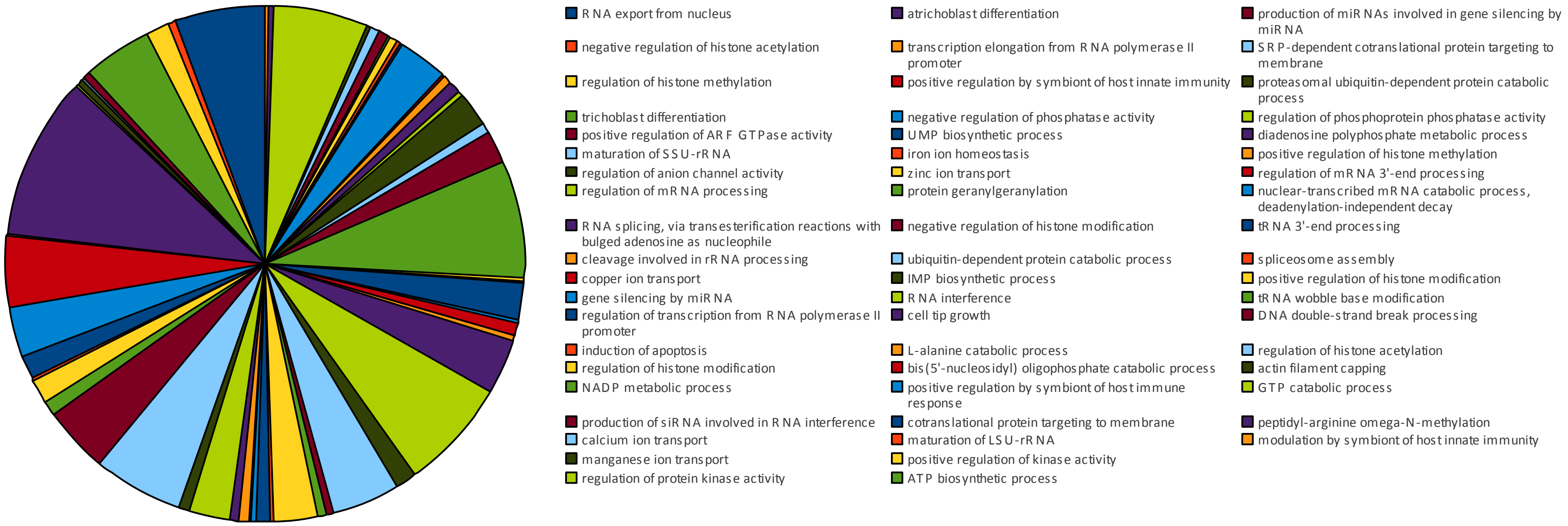

Biological Process: Level 10; Root

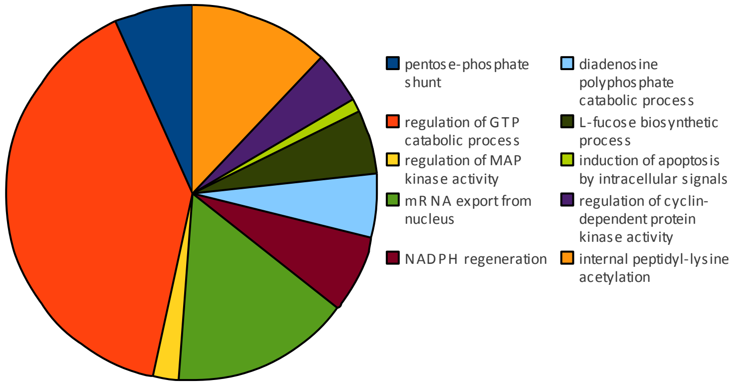

Cellular Components: Level 2; Root

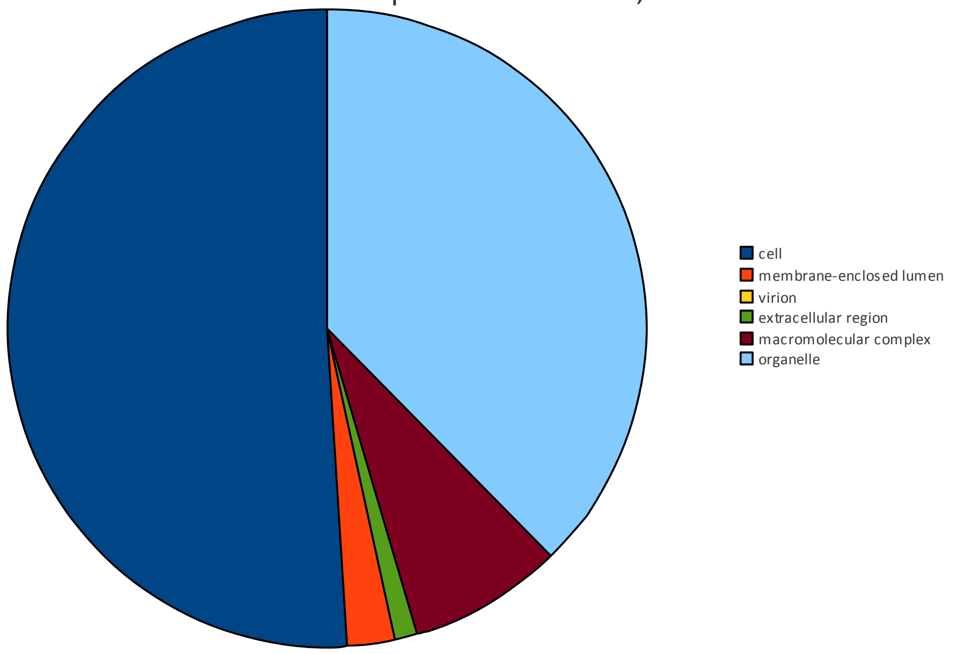

Cellular Components: Level 3; Root

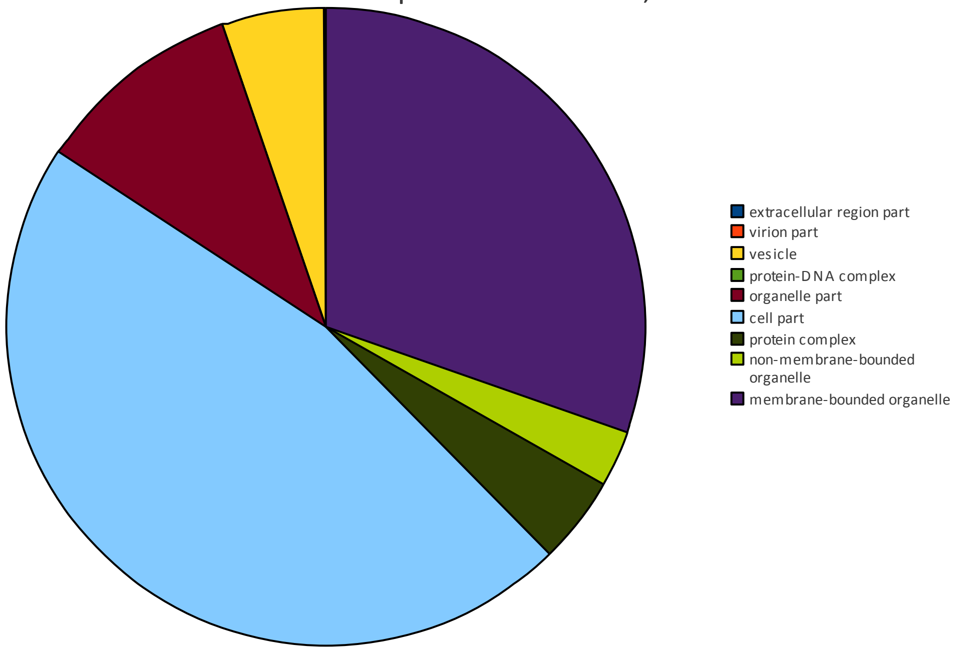

Cellular Components: Level 8; Root

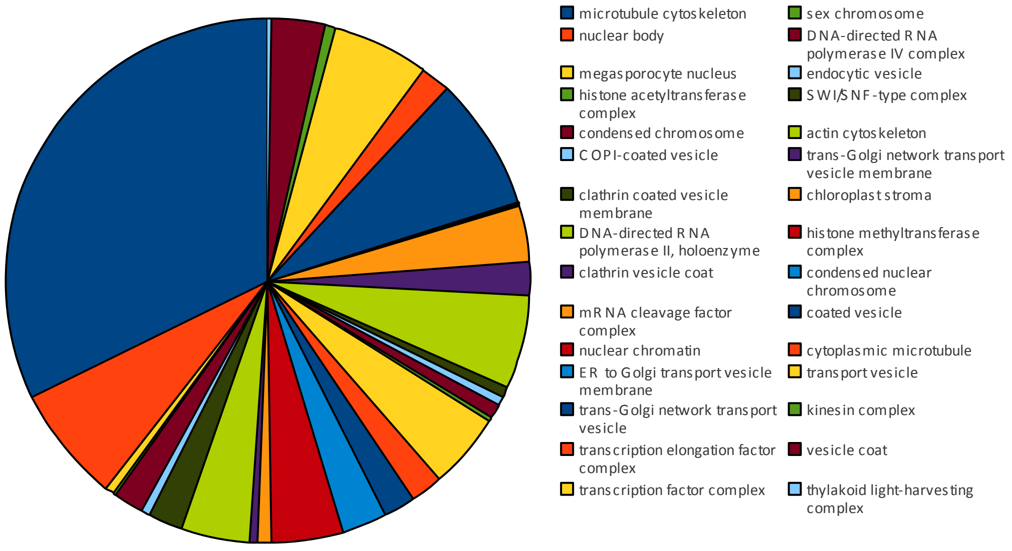

Cellular Components: Level 9; Root

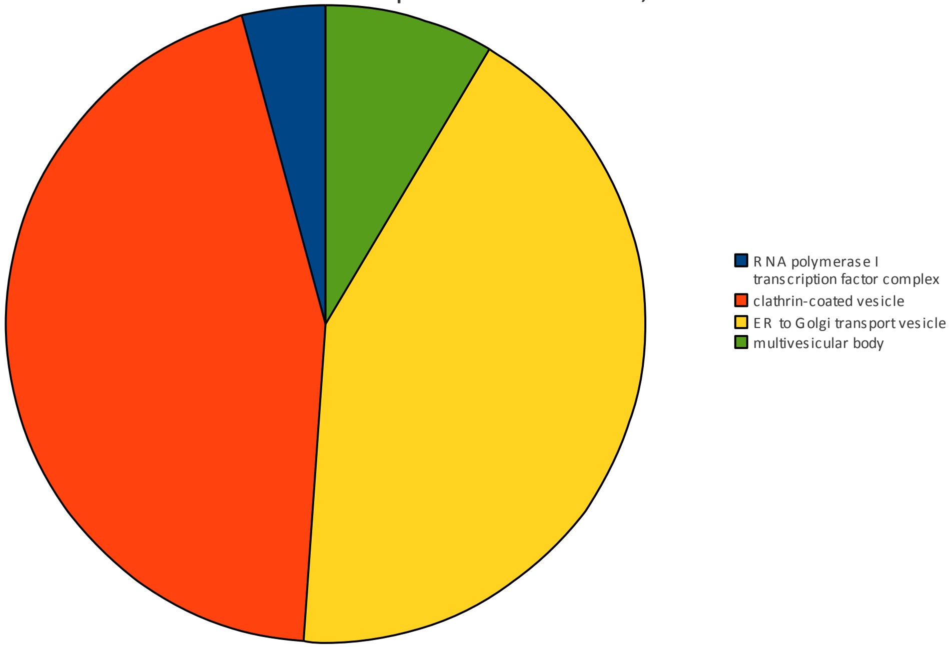

b

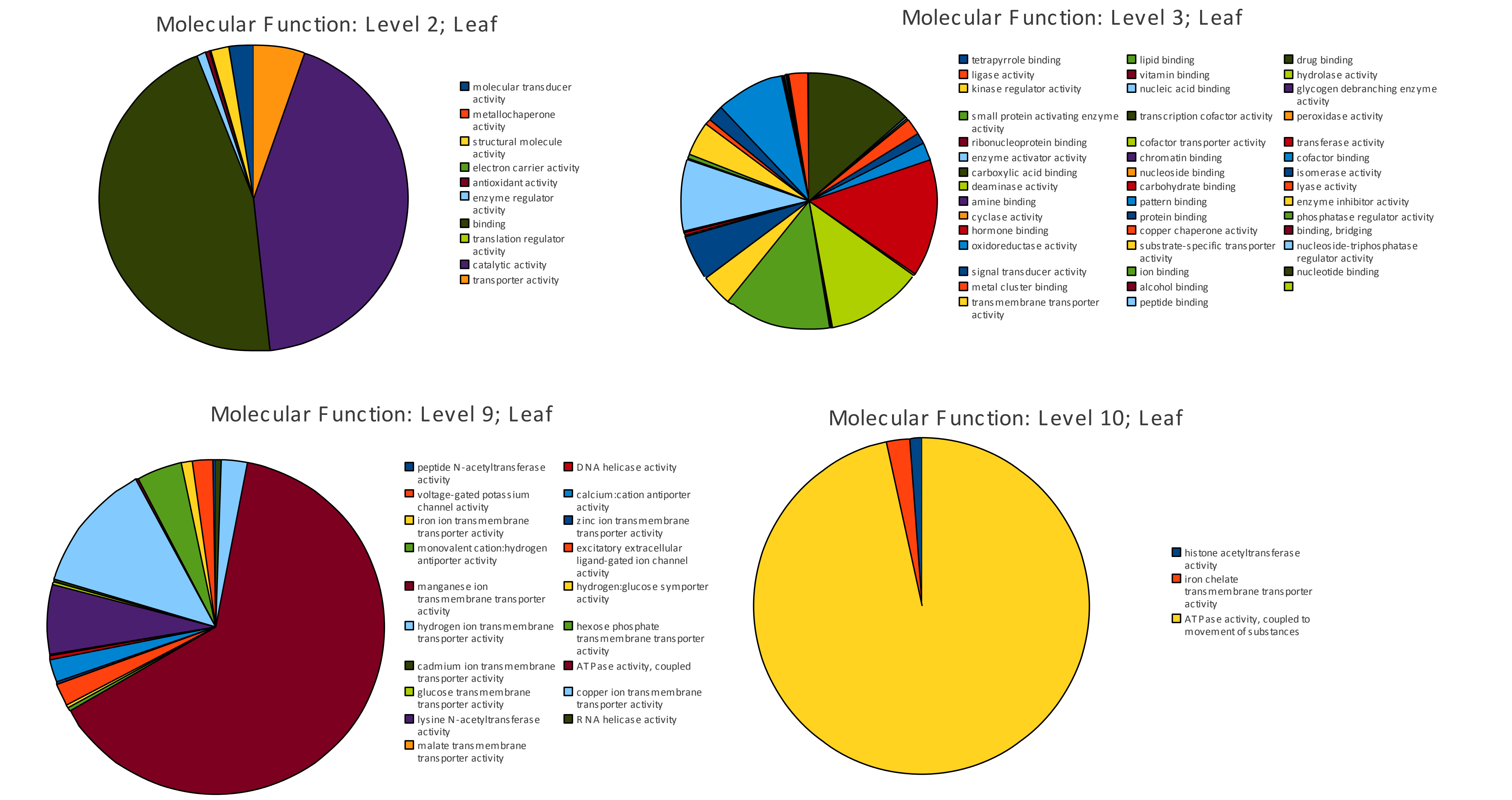
Molecular Function: Level 2; Leaf
molecular transducer activity
metallochaperone activity
structural molecule activity
electron carrier activity
antioxidant activity
enzyme regulator activity
binding
translation regulator activity
catalytic activity
transporter activity
Molecular Function: Level 3; Leaf
tetrapyrrole binding
ligase activity
kinase regulator activity
small protein activating enzyme activity
ribonucleoprotein binding
enzyme activator activity
carboxylic acid binding
deaminase activity
amine binding
cyclase activity
hormone binding
oxidoreductase activity
signal transducer activity
metal cluster binding
transmembrane transporter activity
lipid binding
vitamin binding
nucleic acid binding
transcription cofactor activity
cofactor transporter activity
chromatin binding
nucleoside binding
carbohydrate binding
pattern binding
protein binding
copper chaperone activity
substrate-specific transporter activity
ion binding
alcohol binding
peptide binding
drug binding
hydrolase activity
glycogen debranching enzyme activity
peroxidase activity
transferase activity
cofactor binding
isomerase activity
lyase activity
enzyme inhibitor activity
phosphatase regulator activity
binding, bridging
nucleoside-triphosphatase regulator activity
nucleotide binding
Molecular Function: Level 9; Leaf
peptide N-acetyltransferase activity
voltage-gated potassium channel activity
iron ion transmembrane transporter activity
monovalent cation:hydrogen antiporter activity
manganese ion transmembrane transporter activity
hydrogen ion transmembrane transporter activity
cadmium ion transmembrane transporter activity
glucose transmembrane transporter activity
lysine N-acetyltransferase activity
malate transmembrane transporter activity
DNA helicase activity
calcium:cation antiporter activity
zinc ion transmembrane transporter activity
excitatory extracellular ligand-gated ion channel activity
hydrogen:glucose symporter activity
hexose phosphate transmembrane transporter activity
ATPase activity, coupled
copper ion transmembrane transporter activity
RNA helicase activity
Molecular Function: Level 10; Leaf
histone acetyltransferase activity
iron chelate transmembrane transporter activity
ATPase activity, coupled to movement of substances

Biological Process: Level 2; Leaf

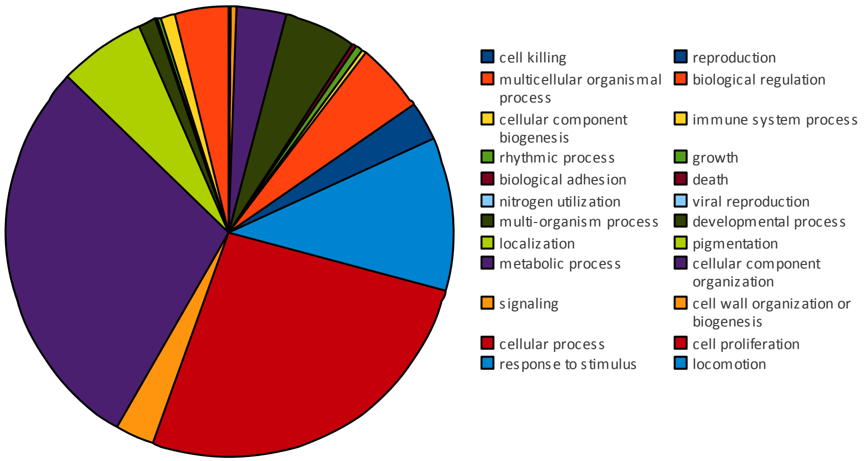

Biological Process: Level 3; Root

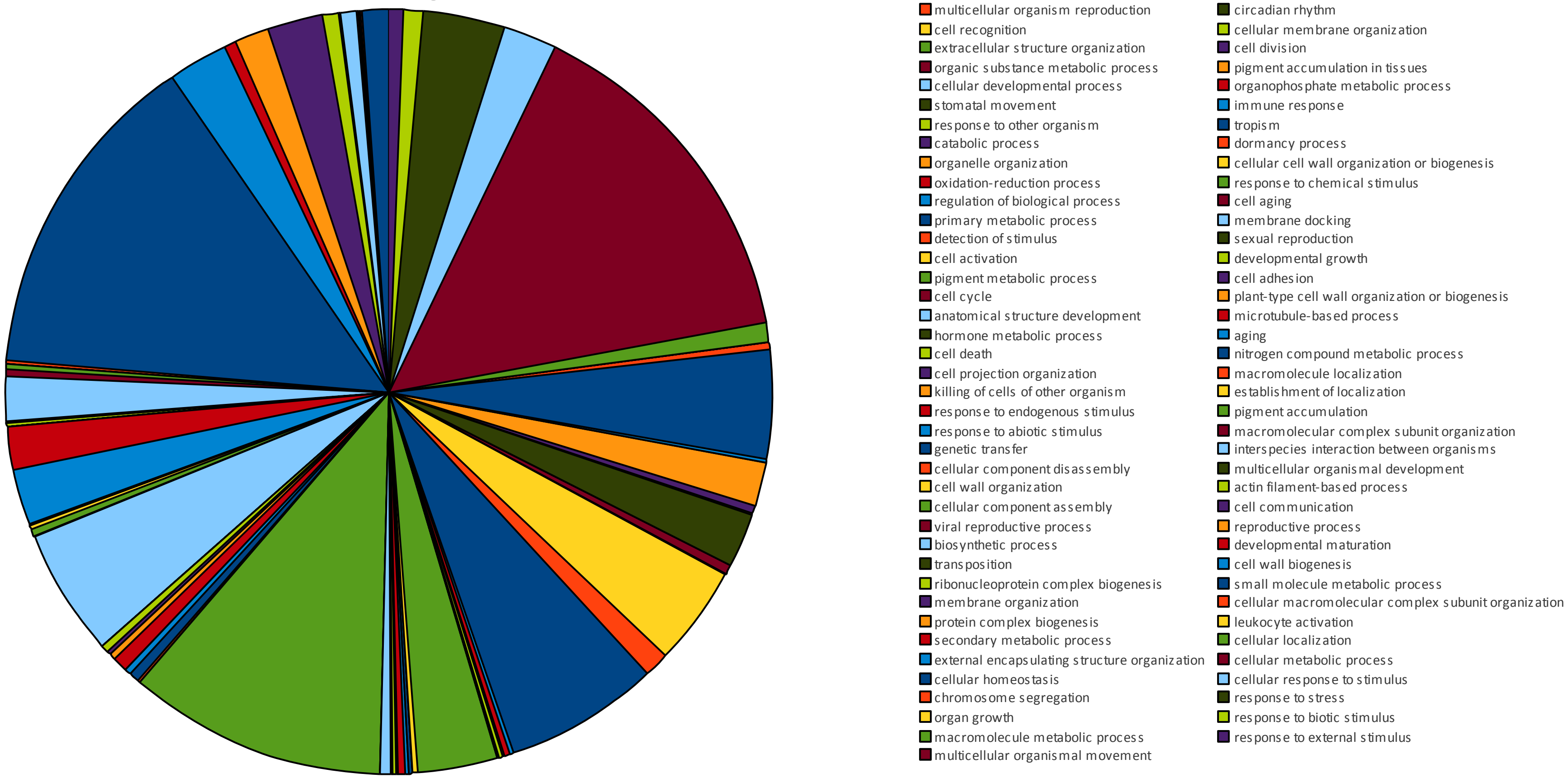

Biological Process: Level 9; Leaf

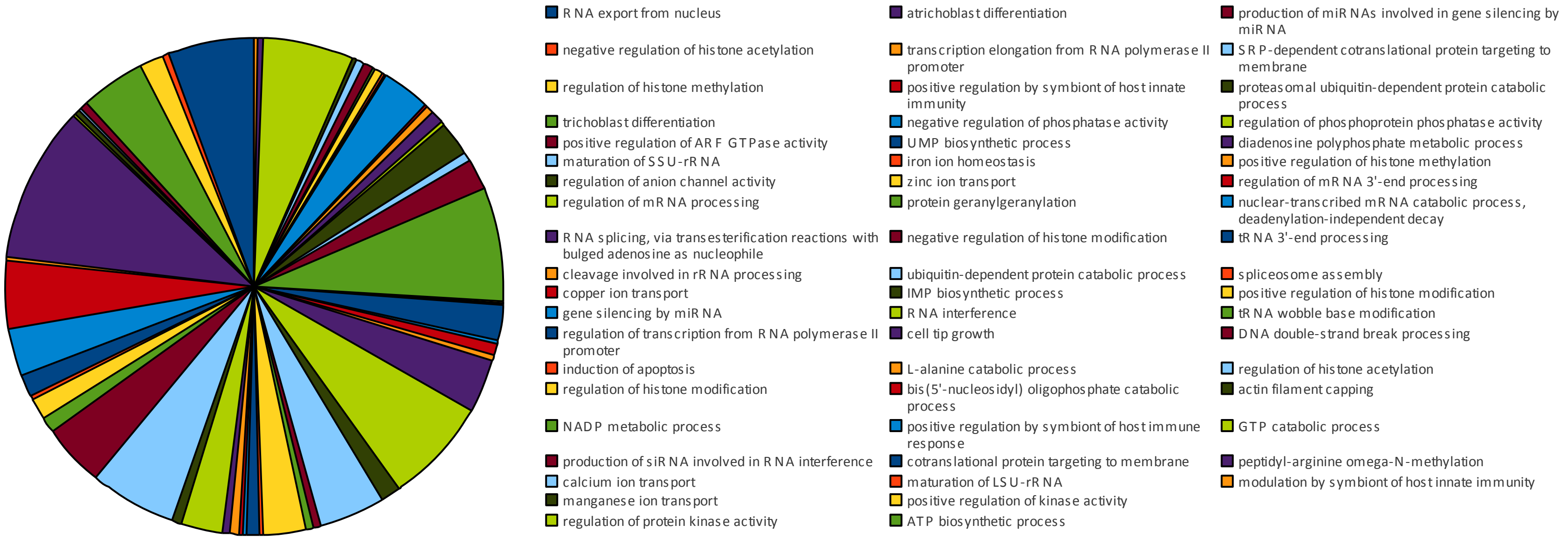

Biological Process: Level 10; Leaf

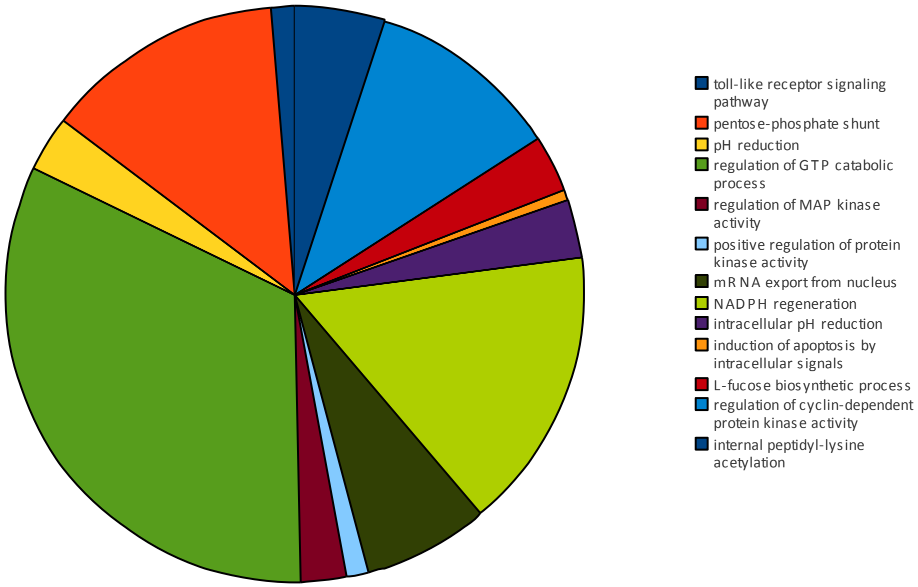

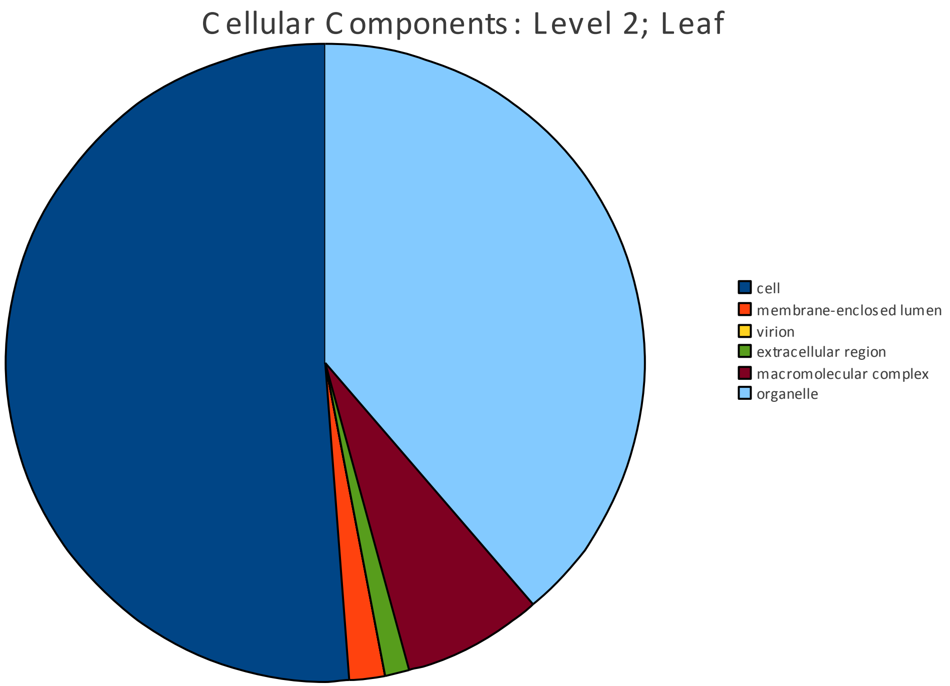
Cellular Components: Level 2; Leaf
cell
membrane-enclosed lumen
virion
extracellular region
macromolecular complex
organelle

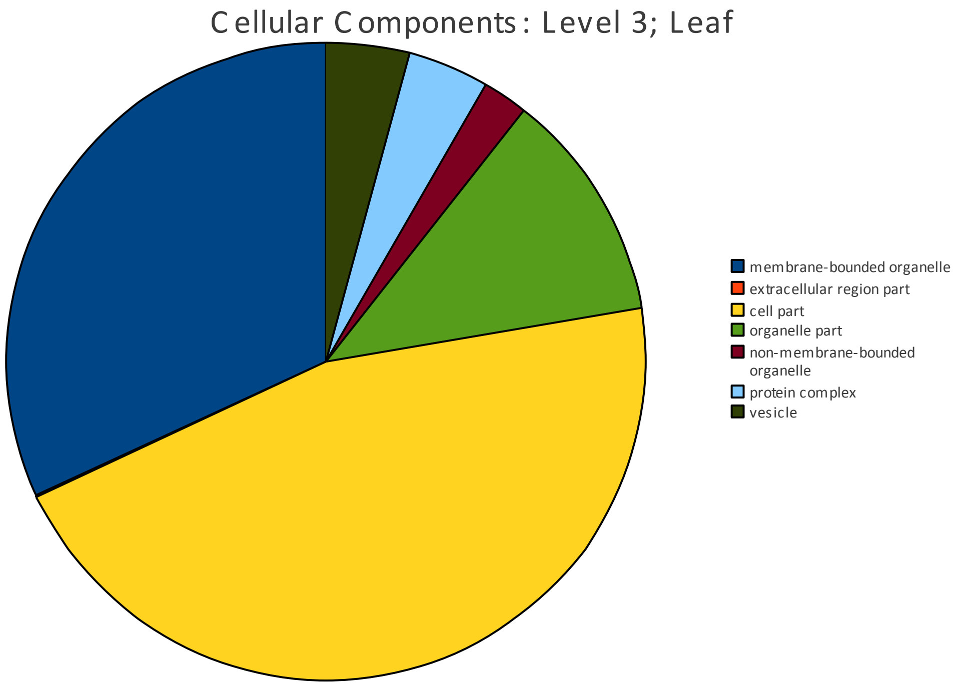
Cellular Components: Level 3; Leaf
membrane-bounded organelle
extracellular region part
cell part
organelle part
non-membrane-bounded organelle
protein complex
vesicle

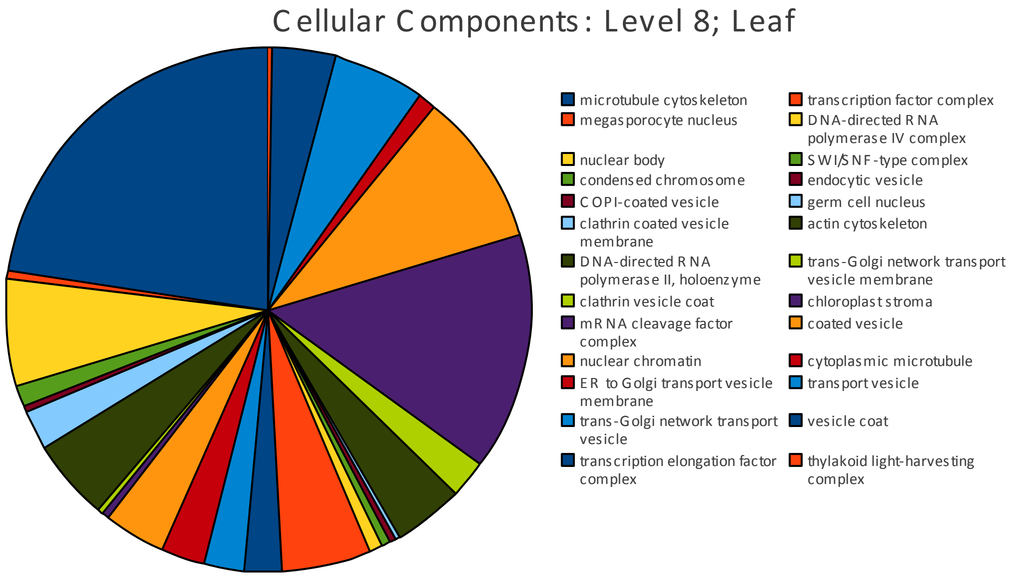
Cellular Components: Level 8; Leaf
microtubule cytoskeleton
transcription factor complex
megasporocyte nucleus
DNA-directed RNA polymerase IV complex
nuclear body
SWI/SNF-type complex
condensed chromosome
endocytic vesicle
COPI-coated vesicle
germ cell nucleus
clathrin coated vesicle membrane
actin cytoskeleton
DNA-directed RNA polymerase II, holoenzyme
trans-Golgi network transport vesicle membrane
clathrin vesicle coat
chloroplast stroma
mRNA cleavage factor complex
coated vesicle
nuclear chromatin
cytoplasmic microtubule
ER to Golgi transport vesicle membrane
transport vesicle
trans-Golgi network transport vesicle
vesicle coat
transcription elongation factor complex
thylakoid light-harvesting complex

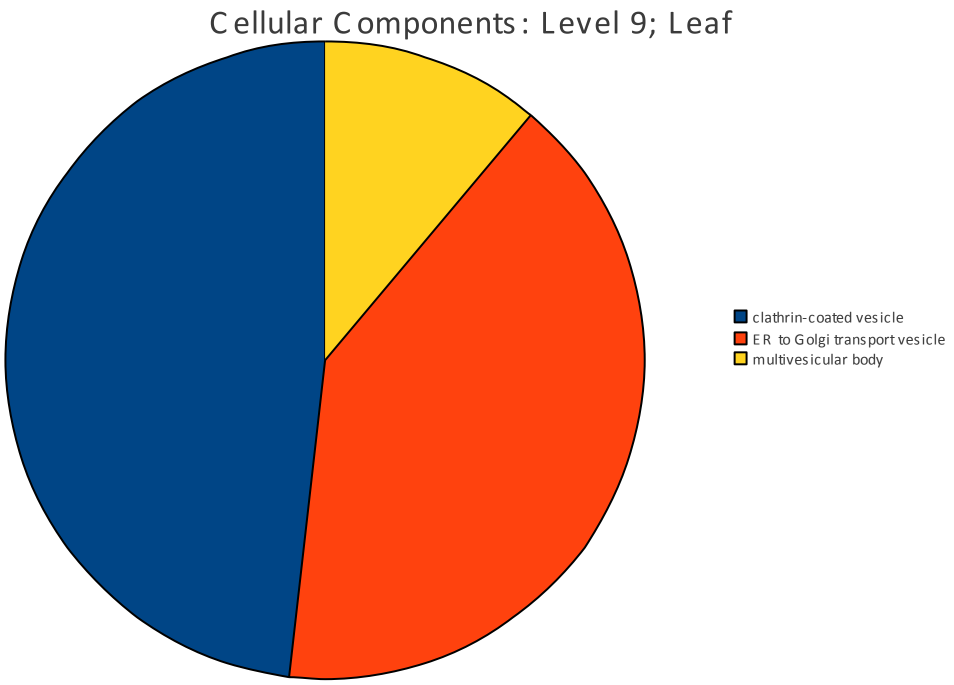
Cellular Components: Level 9; Leaf
clathrin-coated vesicle
ER to Golgi transport vesicle
multivesicular body

c

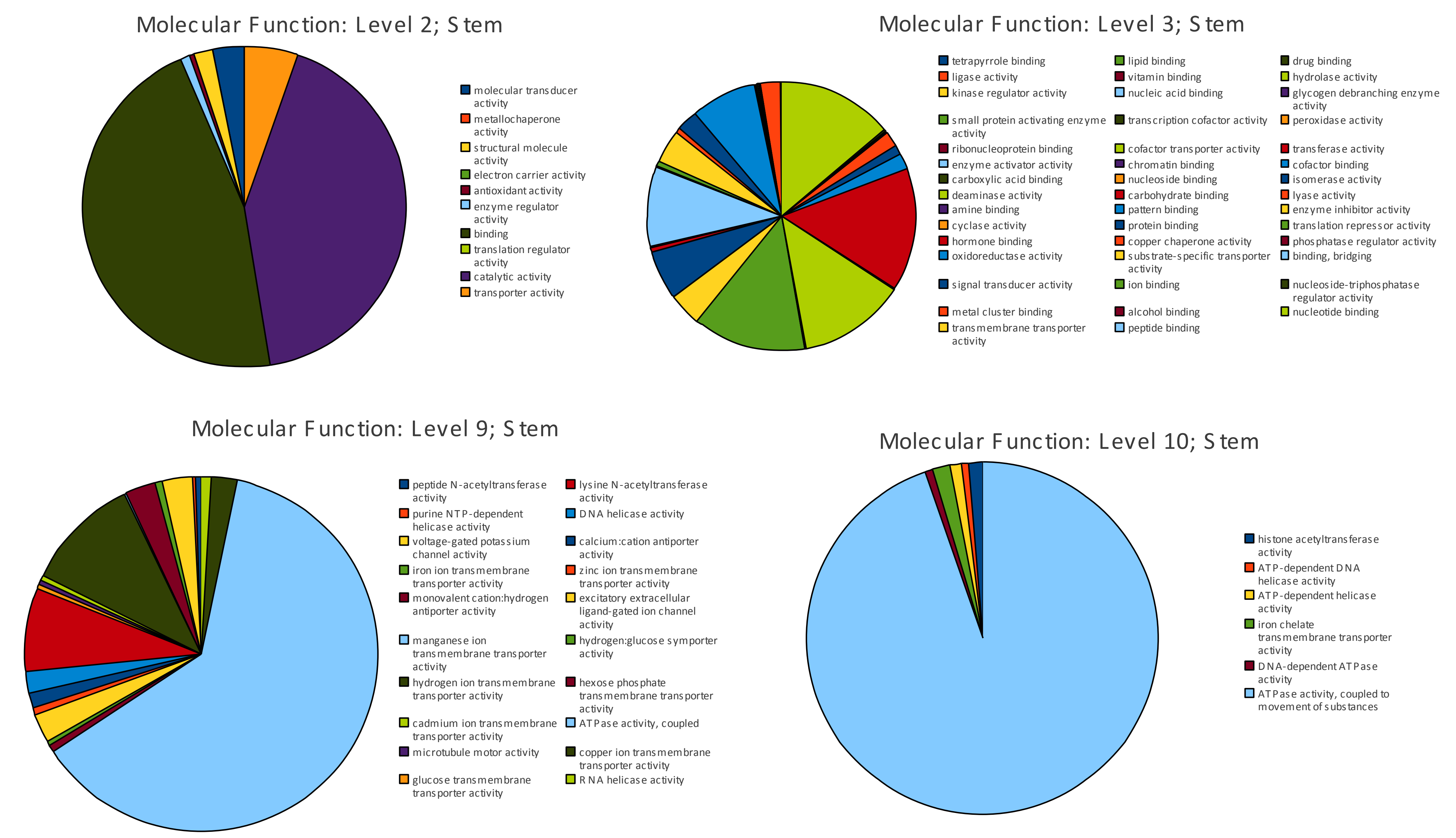

Molecular Function: Level 2; Stem
molecular transducer activity
metallochaperone activity
structural molecule activity
electron carrier activity
antioxidant activity
enzyme regulator activity
binding
translation regulator activity
catalytic activity
transporter activity
Molecular Function: Level 3; Stem
tetrapyrrole binding
lipid binding
drug binding
ligase activity
vitamin binding
hydrolase activity
kinase regulator activity
nucleic acid binding
glycogen debranching enzyme activity
small protein activating enzyme activity
transcription cofactor activity
peroxidase activity
ribonucleoprotein binding
cofactor transporter activity
transferase activity
enzyme activator activity
chromatin binding
cofactor binding
carboxylic acid binding
nucleoside binding
isomerase activity
deaminase activity
carbohydrate binding
lyase activity
amine binding
pattern binding
enzyme inhibitor activity
cyclase activity
protein binding
translation repressor activity
hormone binding
copper chaperone activity
phosphatase regulator activity
oxidoreductase activity
substrate-specific transporter activity
binding, bridging
signal transducer activity
ion binding
nucleoside-triphosphatase regulator activity
metal cluster binding
alcohol binding
nucleotide binding
transmembrane transporter activity
peptide binding
Molecular Function: Level 9; Stem
peptide N-acetyltransferase activity
lysine N-acetyltransferase activity
purine NTP-dependent helicase activity
DNA helicase activity
voltage-gated potassium channel activity
calcium:cation antiporter activity
iron ion transmembrane transporter activity
zinc ion transmembrane transporter activity
monovalent cation:hydrogen antiporter activity
excitatory extracellular ligand-gated ion channel activity
manganese ion transmembrane transporter activity
hydrogen:glucose symporter activity
hydrogen ion transmembrane transporter activity
hexose phosphate transmembrane transporter activity
cadmium ion transmembrane transporter activity
ATPase activity, coupled
microtubule motor activity
copper ion transmembrane transporter activity
glucose transmembrane transporter activity
RNA helicase activity
Molecular Function: Level 10; Stem
histone acetyltransferase activity
ATP-dependent DNA helicase activity
ATP-dependent helicase activity
iron chelate transmembrane transporter activity
DNA-dependent ATPase activity
ATPase activity, coupled to movement of substances

Biological Process: Level 2; Stem

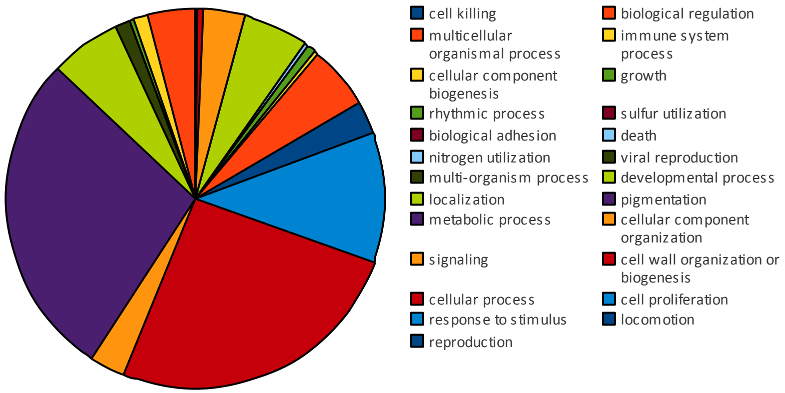

Biological Process: Level 3; Stem

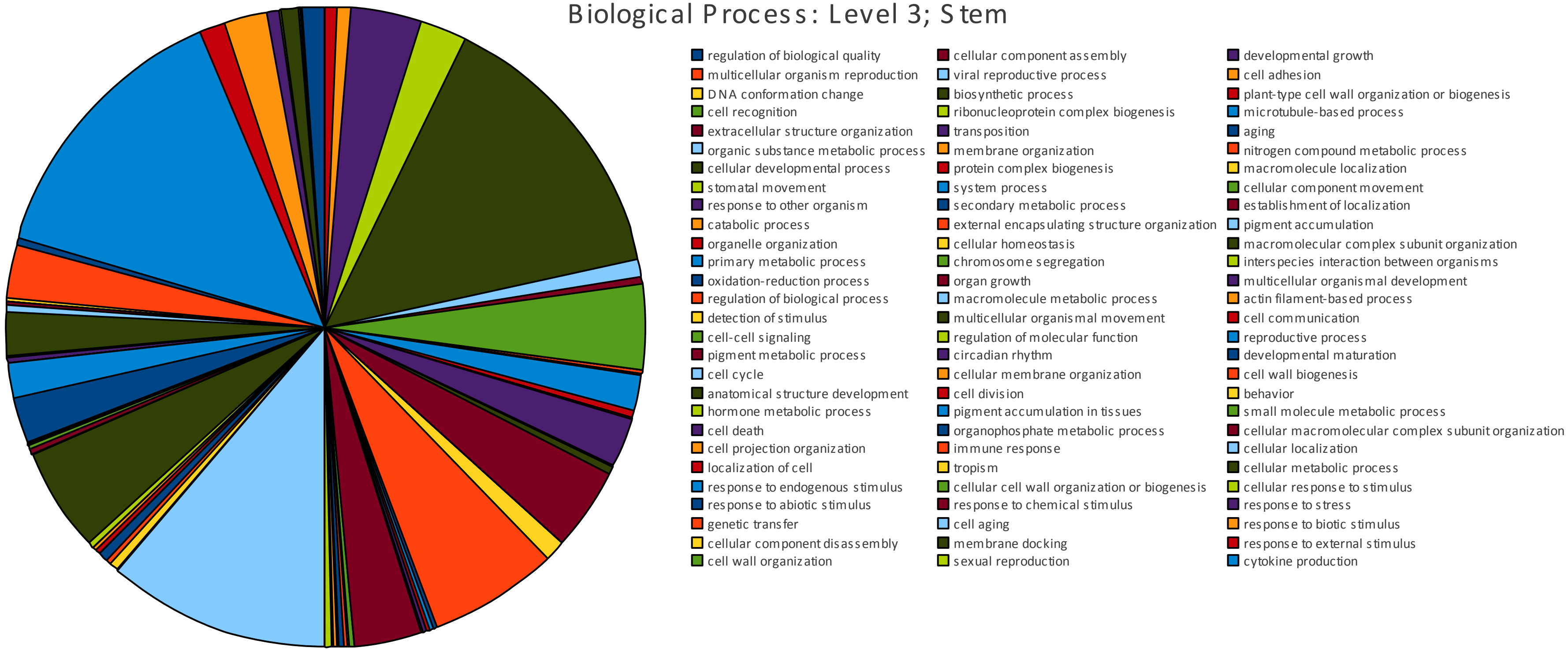

Biological Process: Level 9; Stem

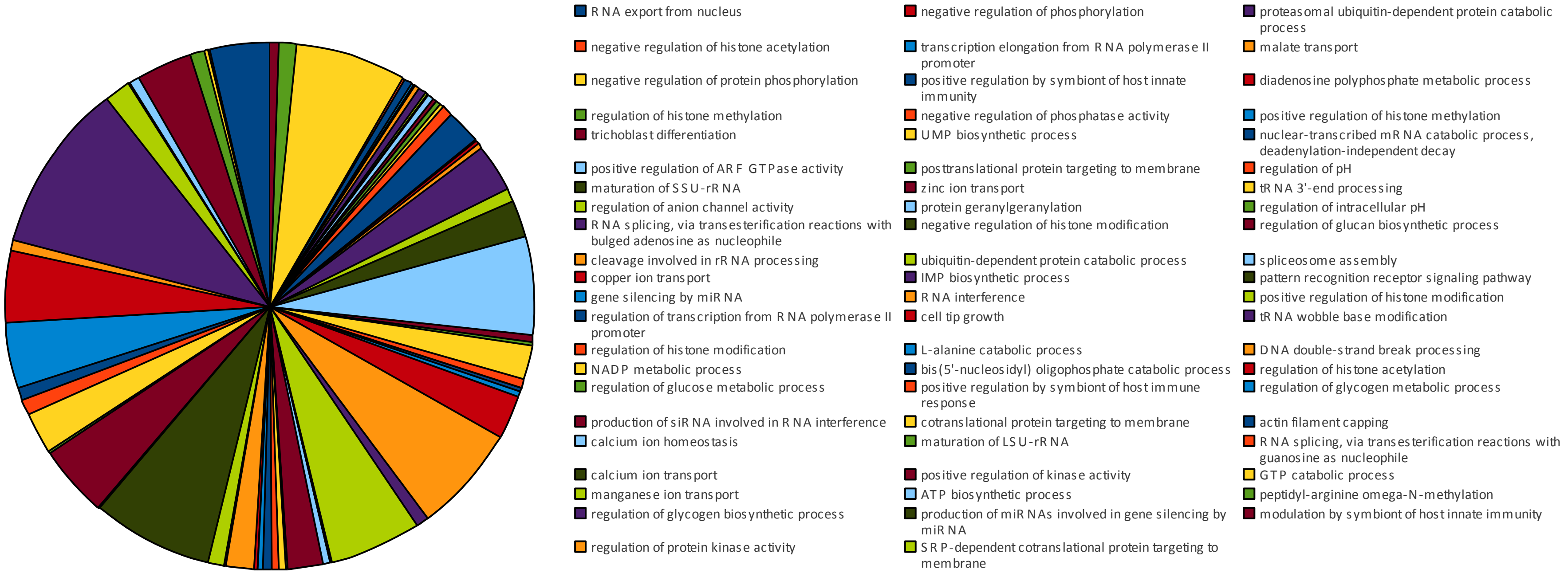

Biological Process: Level 10; Stem

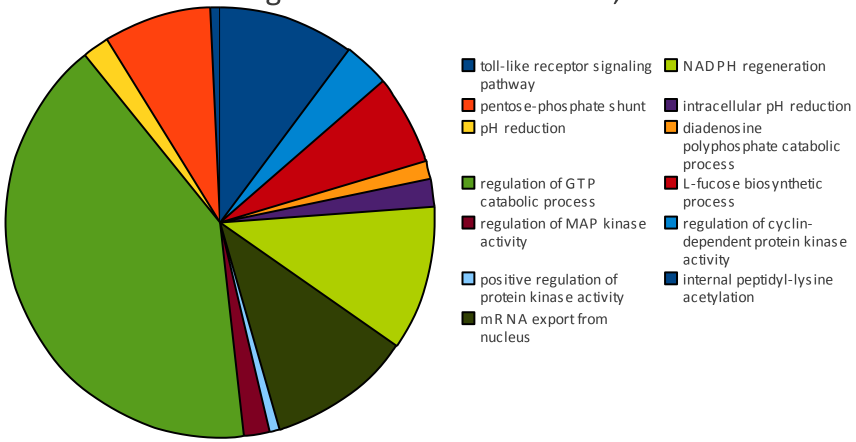

Cellular Components: Level 2; Stem

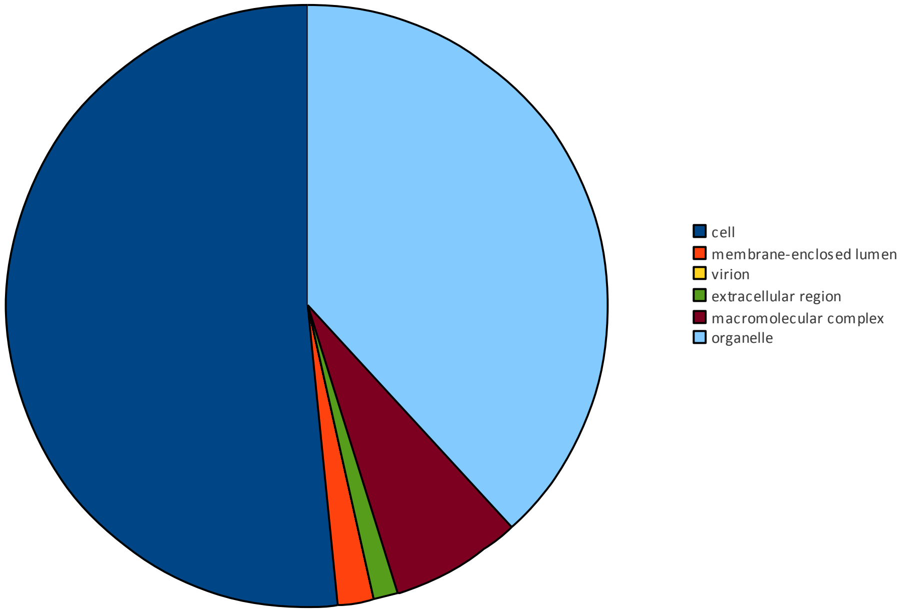

Cellular Components: Level 3; Stem

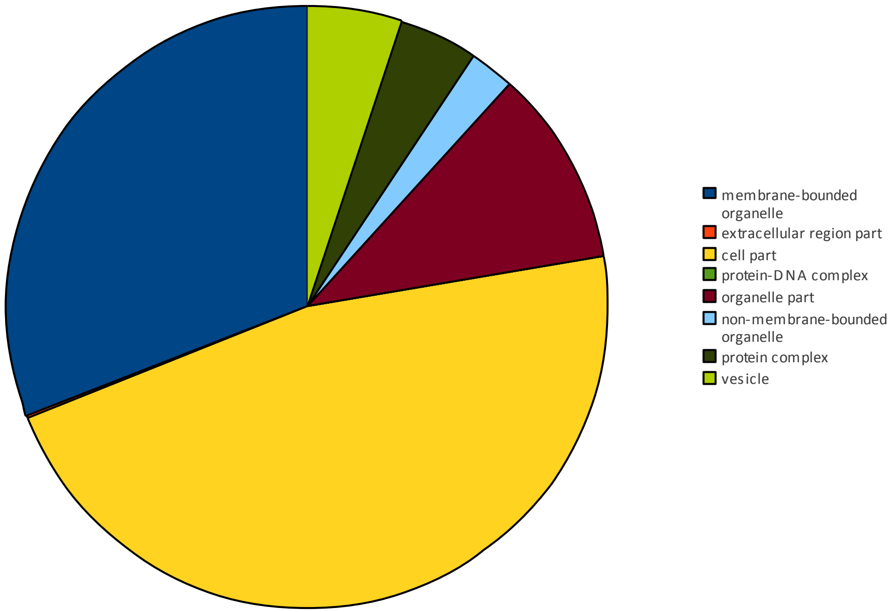

Cellular Components: Level 8; Stem

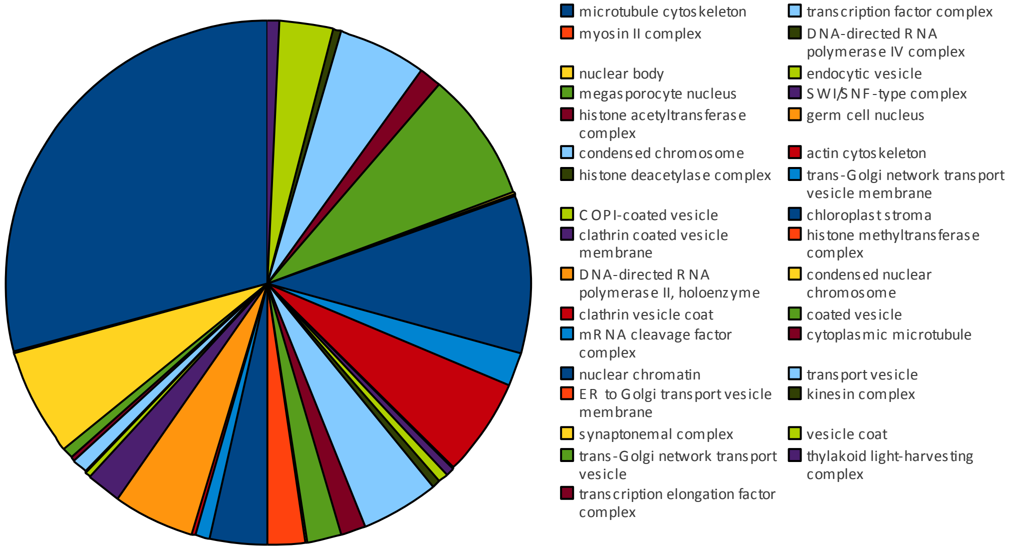

Cellular Components: Level 9; Stem

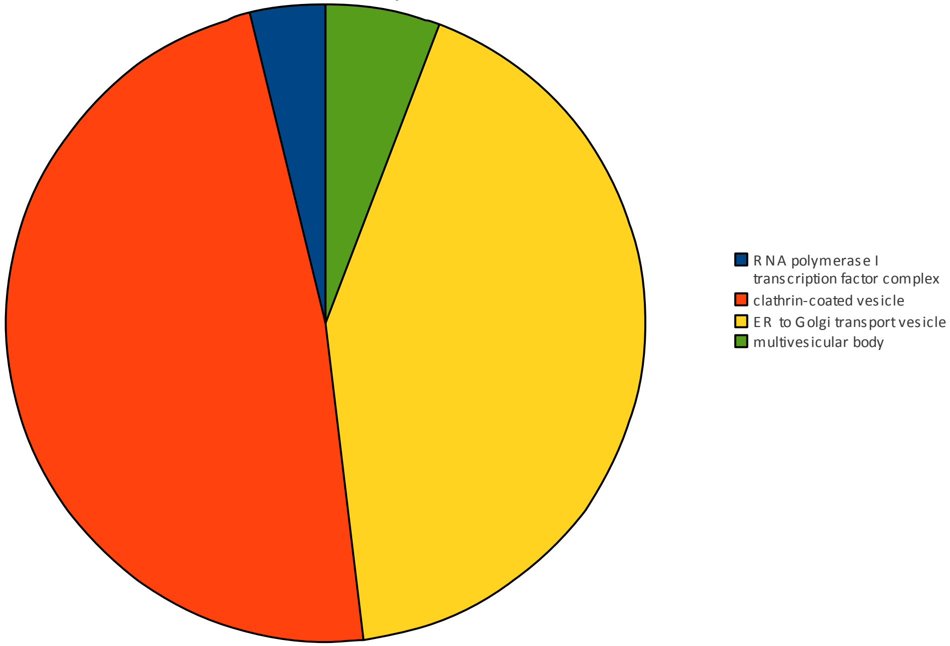

d

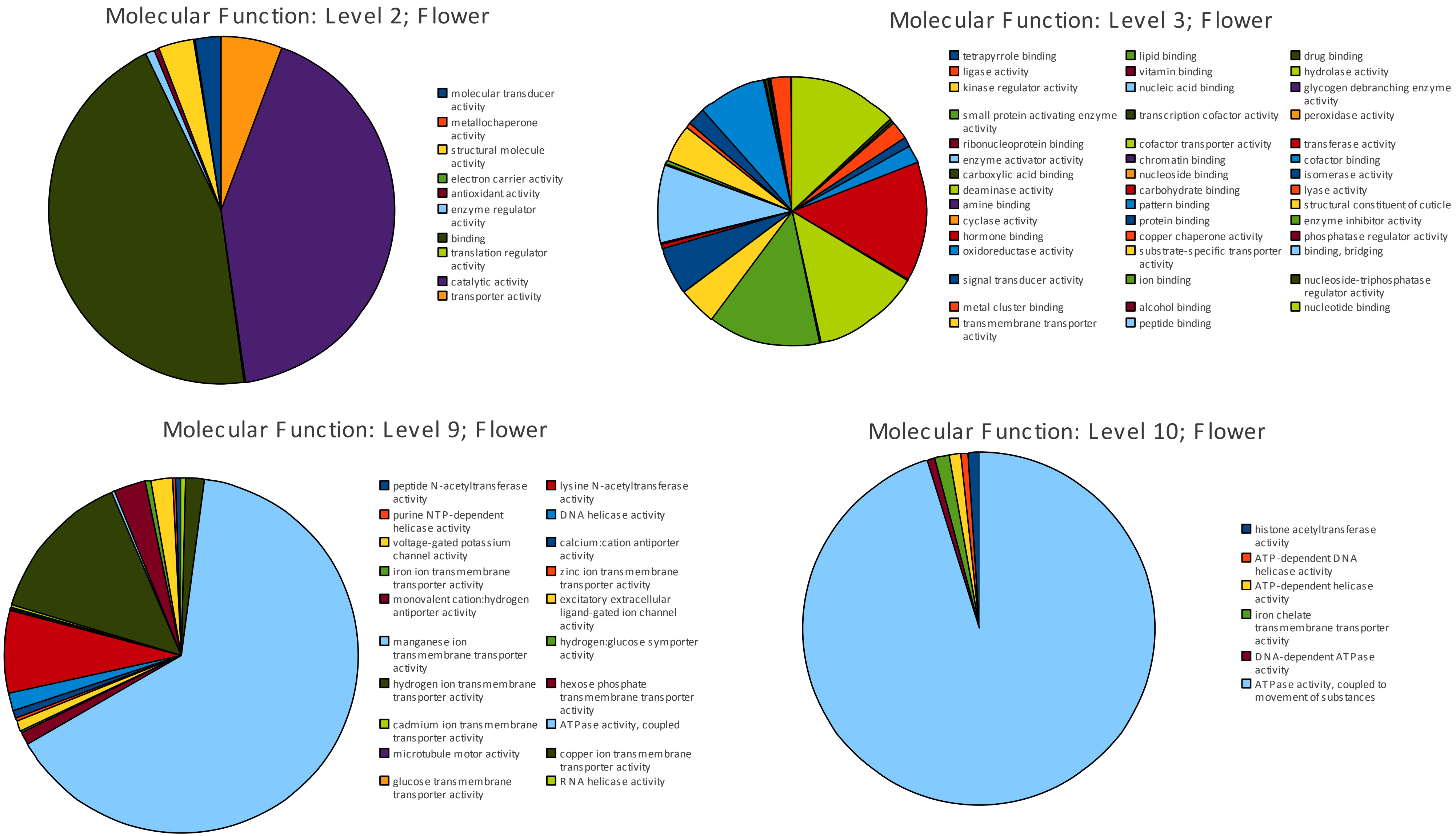
Molecular Function: Level 2; Flower
molecular transducer activity
metallochaperone activity
structural molecule activity
electron carrier activity
antioxidant activity
enzyme regulator activity
binding
translation regulator activity
catalytic activity
transporter activity
Molecular Function: Level 3; Flower
tetrapyrrole binding
lipid binding
drug binding
ligase activity
vitamin binding
hydrolase activity
kinase regulator activity
nucleic acid binding
glycogen debranching enzyme activity
small protein activating enzyme activity
transcription cofactor activity
peroxidase activity
ribonucleoprotein binding
cofactor transporter activity
transferase activity
enzyme activator activity
chromatin binding
cofactor binding
carboxylic acid binding
nucleoside binding
isomerase activity
deaminase activity
carbohydrate binding
lyase activity
amine binding
pattern binding
structural constituent of cuticle
cyclase activity
protein binding
enzyme inhibitor activity
hormone binding
copper chaperone activity
phosphatase regulator activity
oxidoreductase activity
substrate-specific transporter activity
binding, bridging
signal transducer activity
ion binding
nucleoside-triphosphatase regulator activity
metal cluster binding
alcohol binding
nucleotide binding
transmembrane transporter activity
peptide binding
Molecular Function: Level 9; Flower
peptide N-acetyltransferase activity
lysine N-acetyltransferase activity
purine NTP-dependent helicase activity
DNA helicase activity
voltage-gated potassium channel activity
calcium:cation antiporter activity
iron ion transmembrane transporter activity
zinc ion transmembrane transporter activity
monovalent cation:hydrogen antiporter activity
excitatory extracellular ligand-gated ion channel activity
manganese ion transmembrane transporter activity
hydrogen:glucose symporter activity
hydrogen ion transmembrane transporter activity
hexose phosphate transmembrane transporter activity
cadmium ion transmembrane transporter activity
ATPase activity, coupled
microtubule motor activity
copper ion transmembrane transporter activity
glucose transmembrane transporter activity
RNA helicase activity
Molecular Function: Level 10; Flower
histone acetyltransferase activity
ATP-dependent DNA helicase activity
ATP-dependent helicase activity
iron chelate transmembrane transporter activity
DNA-dependent ATPase activity
ATPase activity, coupled to movement of substances

Biological Process: Level 2; Flower

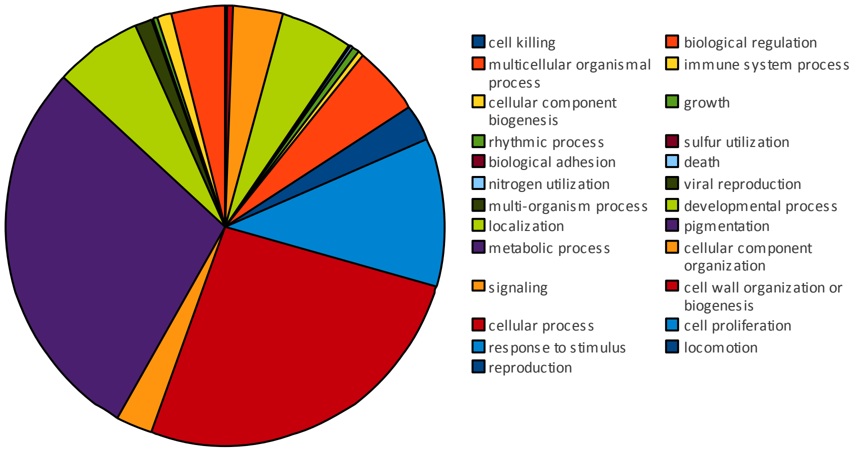

Biological Process: Level 3; Flower

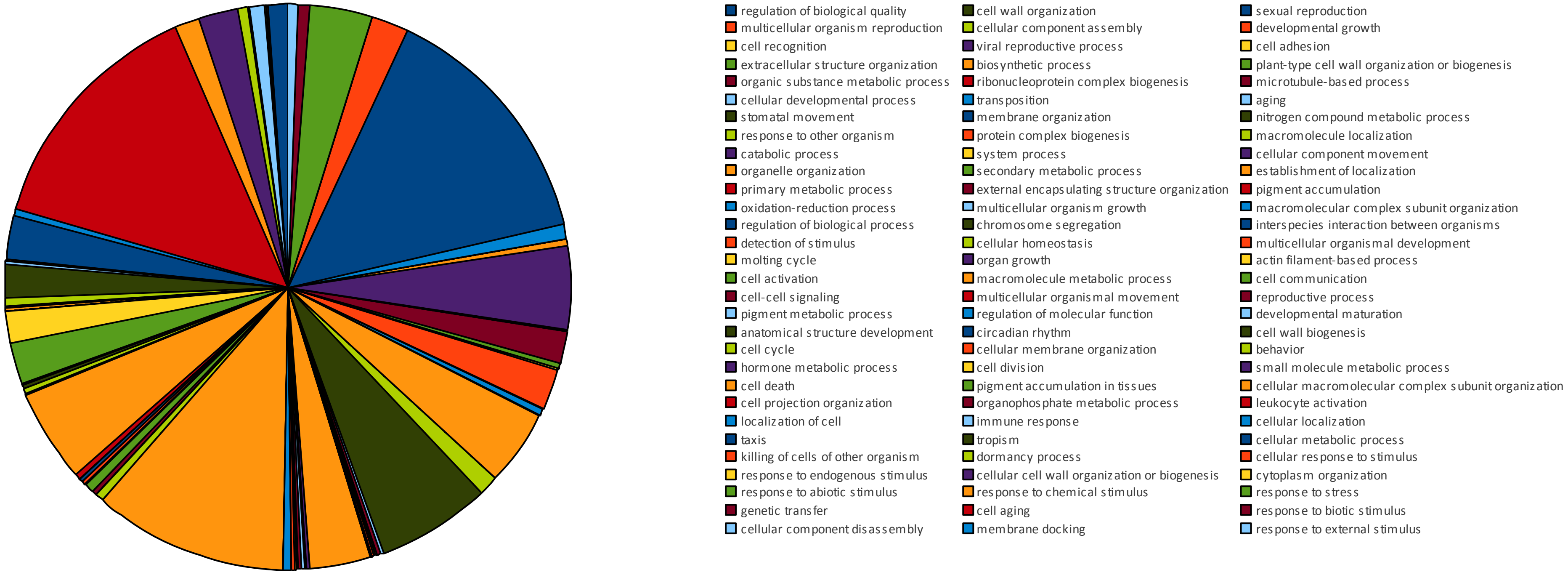

Biological Process: Level9; Flower

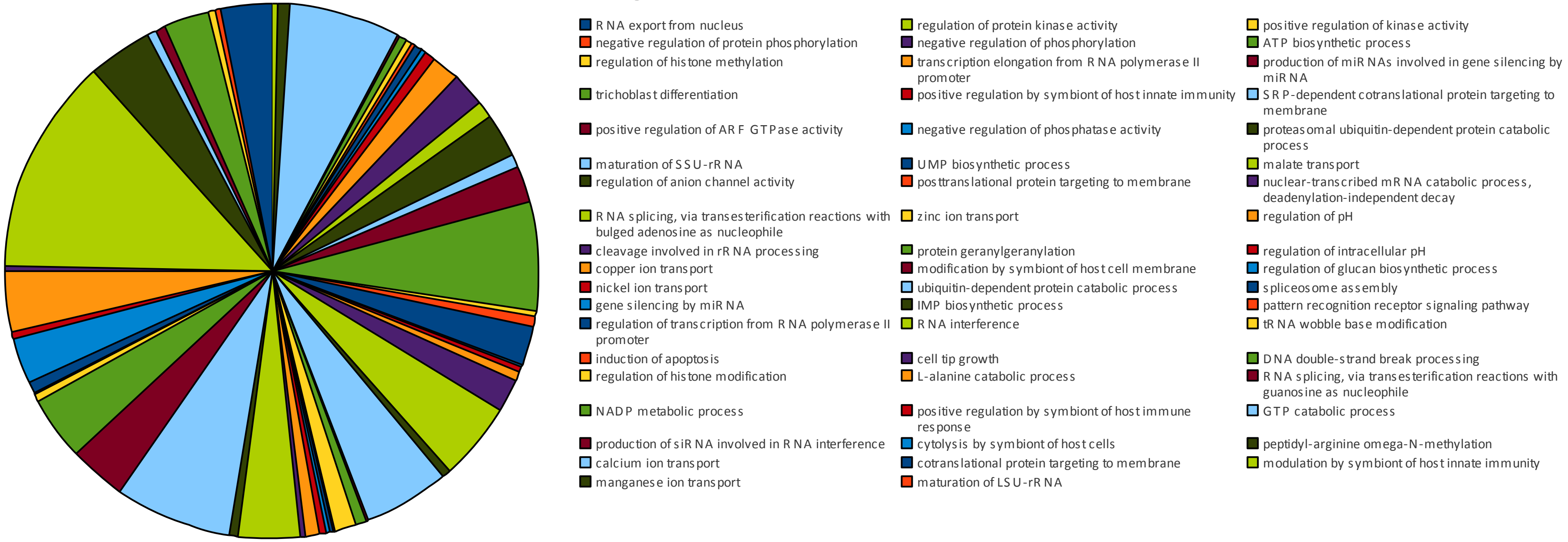

Biological Process: Level 10; Flower

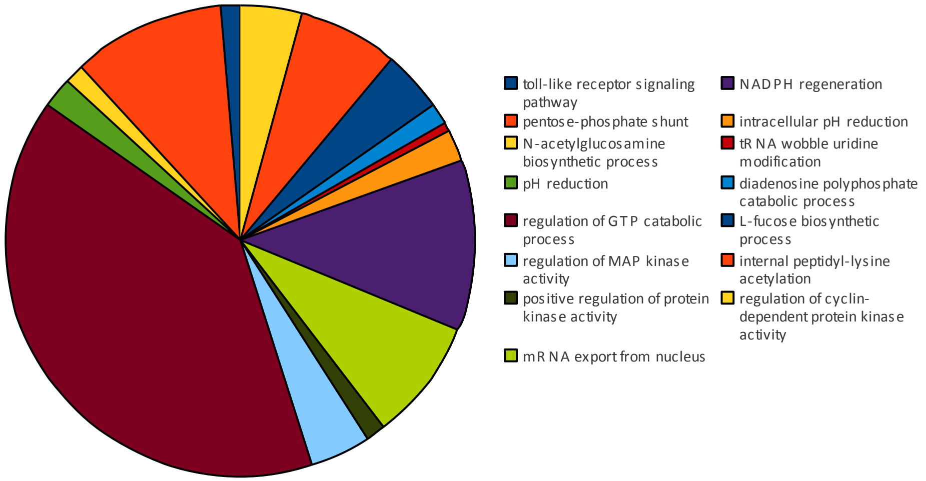

Cellular Components: Level 2; Flower

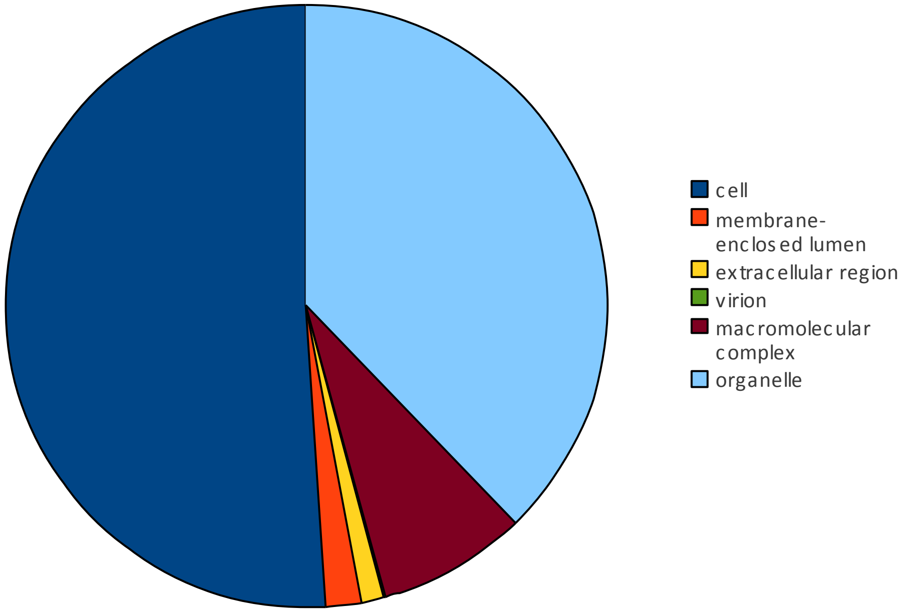

Cellular Components: Level 3; Flower

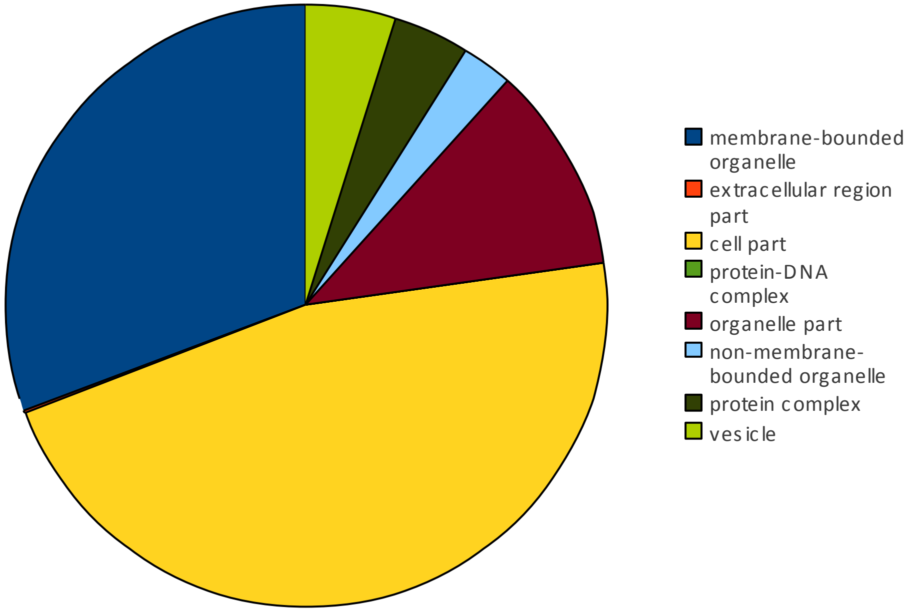

Cellular Components: Level 8; Flower

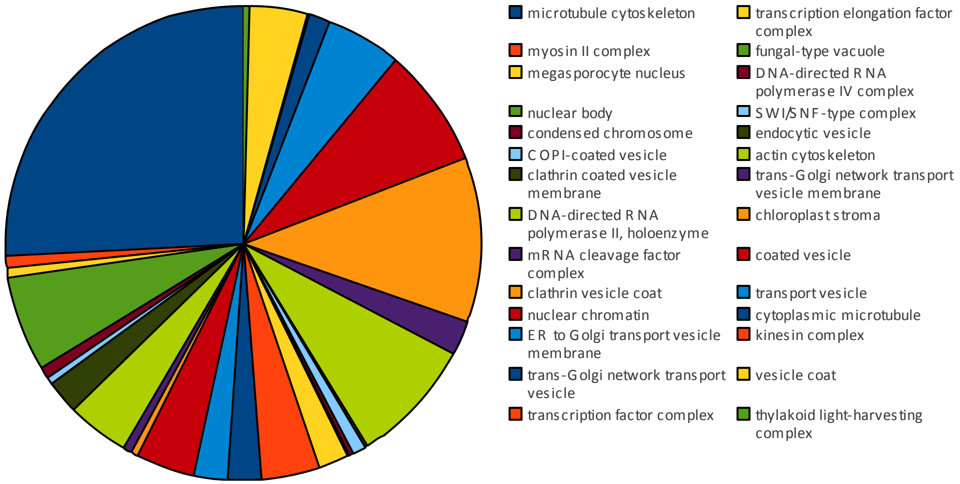

Cellular Components: Level 9; Flower

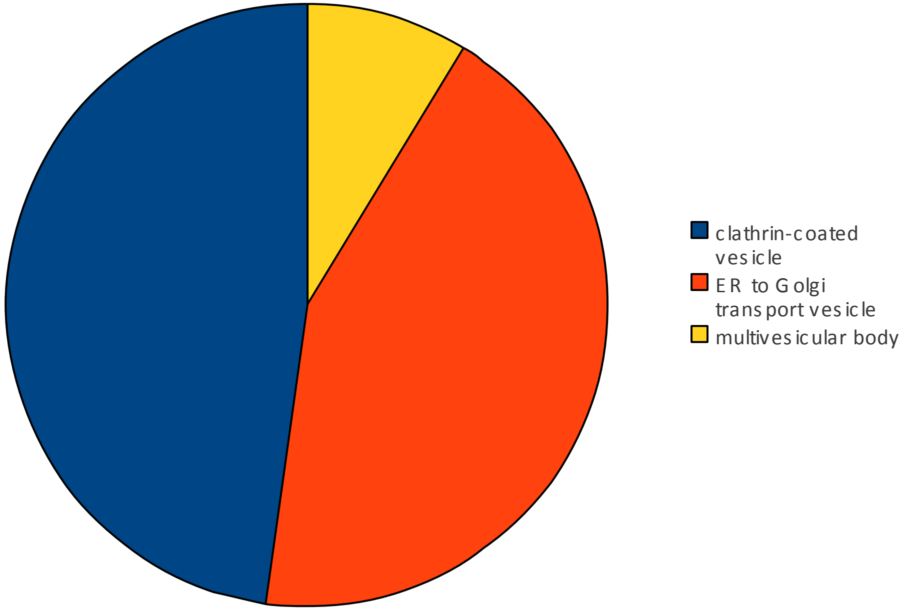

Figure S5
Expression density profiles of neem organ transcriptomes.
The FPKM values of transcripts from each organ (root, leaf, stem and flower) were log2- transformed, and plotted as density curves.

Figure S5

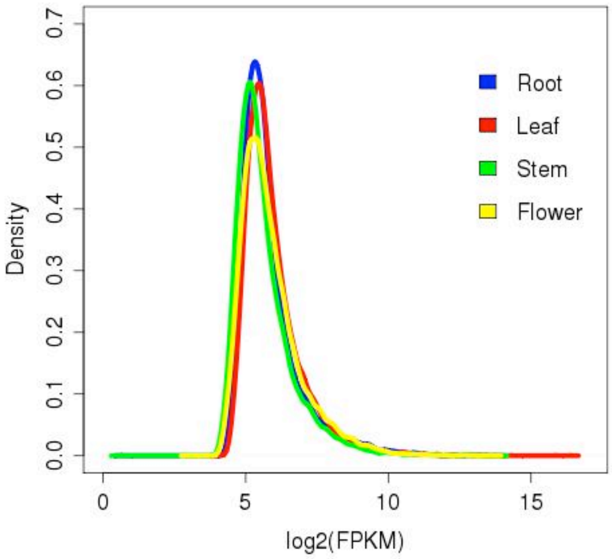
Root
Leaf
Stem
Flower
Density
0.0
0.1
0.2
0.3
0.4
0.5
0.6
0.7
0
5
10
15
log2(FPKM)

Figure S6

GC-correlated expression density profiles of neem organ transcriptomes.
The FPKM values of transcripts from each organ (root, leaf, stem and flower) were log2- transformed, and plotted as density curves after dividing the transcripts into GC-based categories, while ensuring each category contained the same number of transcripts for each given organ. This number is indicated inside parentheses for each organ, along with the average GC (%) in each category.

Figure S6

a Root (3988 transcripts)

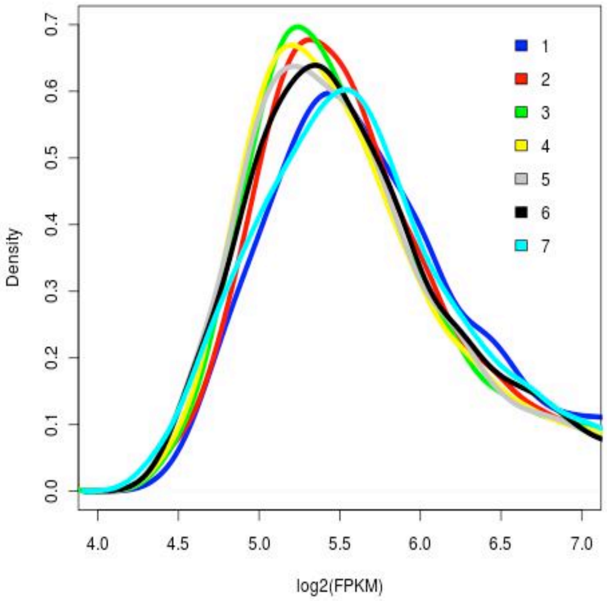

1	2	3	4	5	6	7
21.19	39.46	40.98	42.09	43.16	44.34	46.2

b Leaf (3041 transcripts)

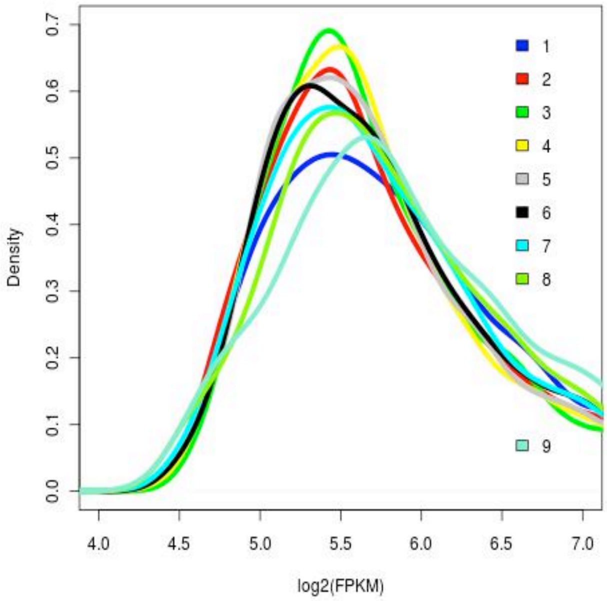

1	2	3	4	5	6	7	8	9
26.1	38.54	40.07	41.07	41.92	42.76	43.67	44.77	46.57

c Stem (3138 transcripts)

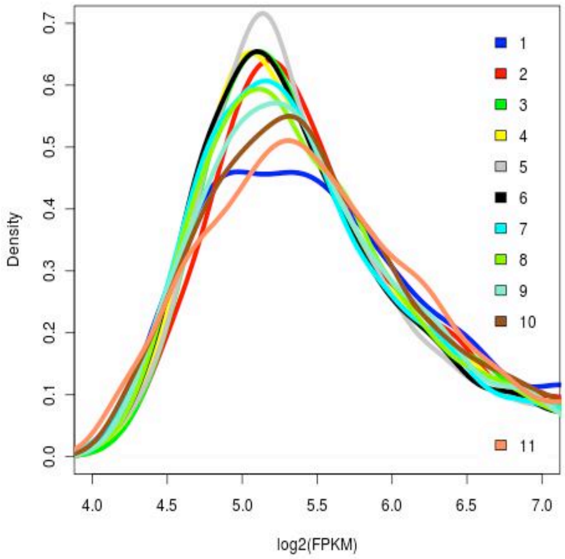

1	2	3	4	5	6	7	8	9	10	11
23.70	38.24	39.68	40.64	41.38	42.07	42.73	43.42	44.25	45.27	46.99

d Flower (3469 transcripts)

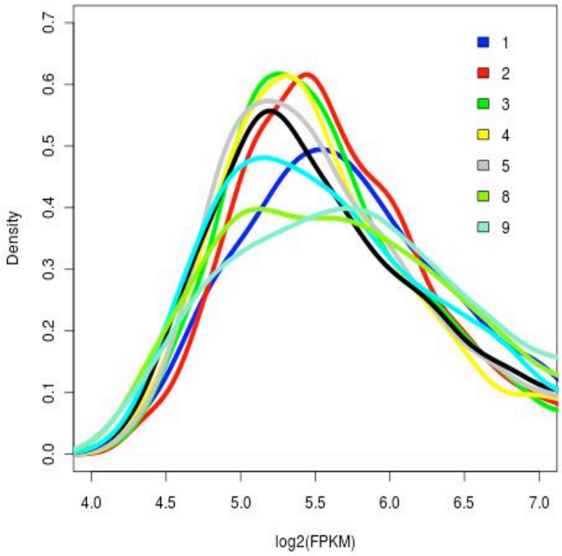

1	2	3	4	5	6	7	8	9
17.52	38.21	39.69	40.68	41.54	42.39	43.34	44.50	46.47

Figure S7
Gene structures of terpenoid biosynthesis genes.
The common set of genes across all five species (neem, *A. thaliana*, *O. sativa*, *C. sinensis* and *V.vinifera*) mapped using KEGG in three pathways, Metabolism of cofactors and vitamins, Metabolism of terpenoids and polyketides, and Biosynthesis of other secondary metabolites, were considered. The transcripts coding for these genes (probe targets in the case of C. sinensis; http://www.affymetrix.com/Auth/analysis/downloads/data/Citrus.target.zip) were mapped to the corresponding genomes, in each species, using PASA The intergenic exon intron structure was obtained from validated PASA genomic scaffold to transcript assemblies and the intron lengths for each gene were compared across species. The gene structures were plotted using a webtool StrDraw (http://www.compgen.unimuenster. de/tools/strdraw/?lang=en&bscl=false), where the boxes represent exons and lines represnt introns.

Figure S7

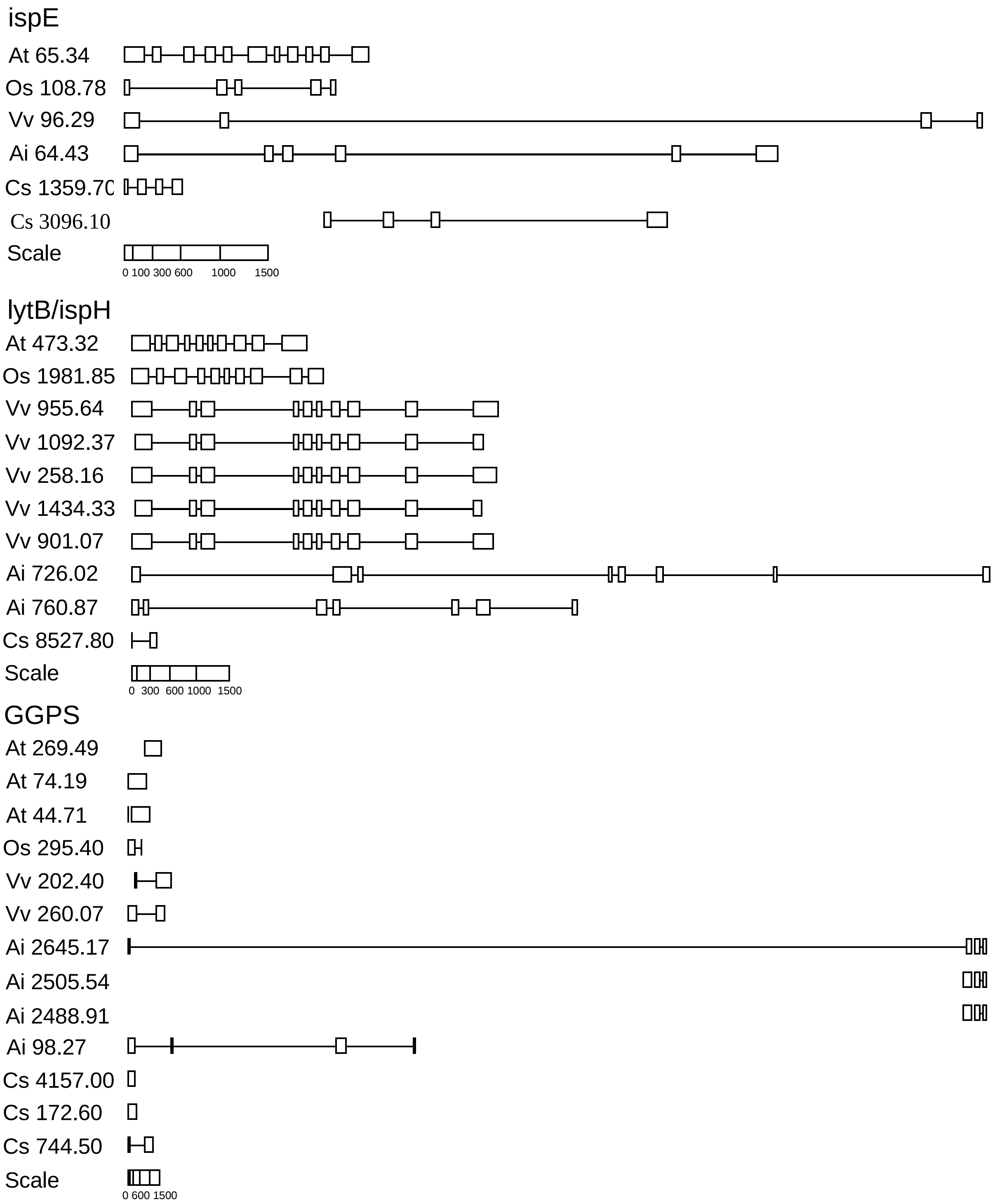
ispE
At 65.34
Os 108.78
Vv 96.29
Ai 64.43
Cs 1359.70
Cs 3096.10
Scale
0 100 300 600 1000 1500
lytB/ispH
At 473.32
Os 1981.85
Vv 955.64
Vv 1092.37
Vv 258.16
Vv 1434.33
Vv 901.07
Ai 726.02
Ai 760.87
Cs 8527.80
Scale
0 300 600 1000 1500
GGPS
At 269.49
At 74.19
At 44.71
Os 295.40
Vv 202.40
Vv 260.07
Ai 2645.17
Ai 2505.54
Ai 2488.91
Ai 98.27
Cs 4157.00
Cs 172.60
Cs 744.50
Scale
0 600 1500

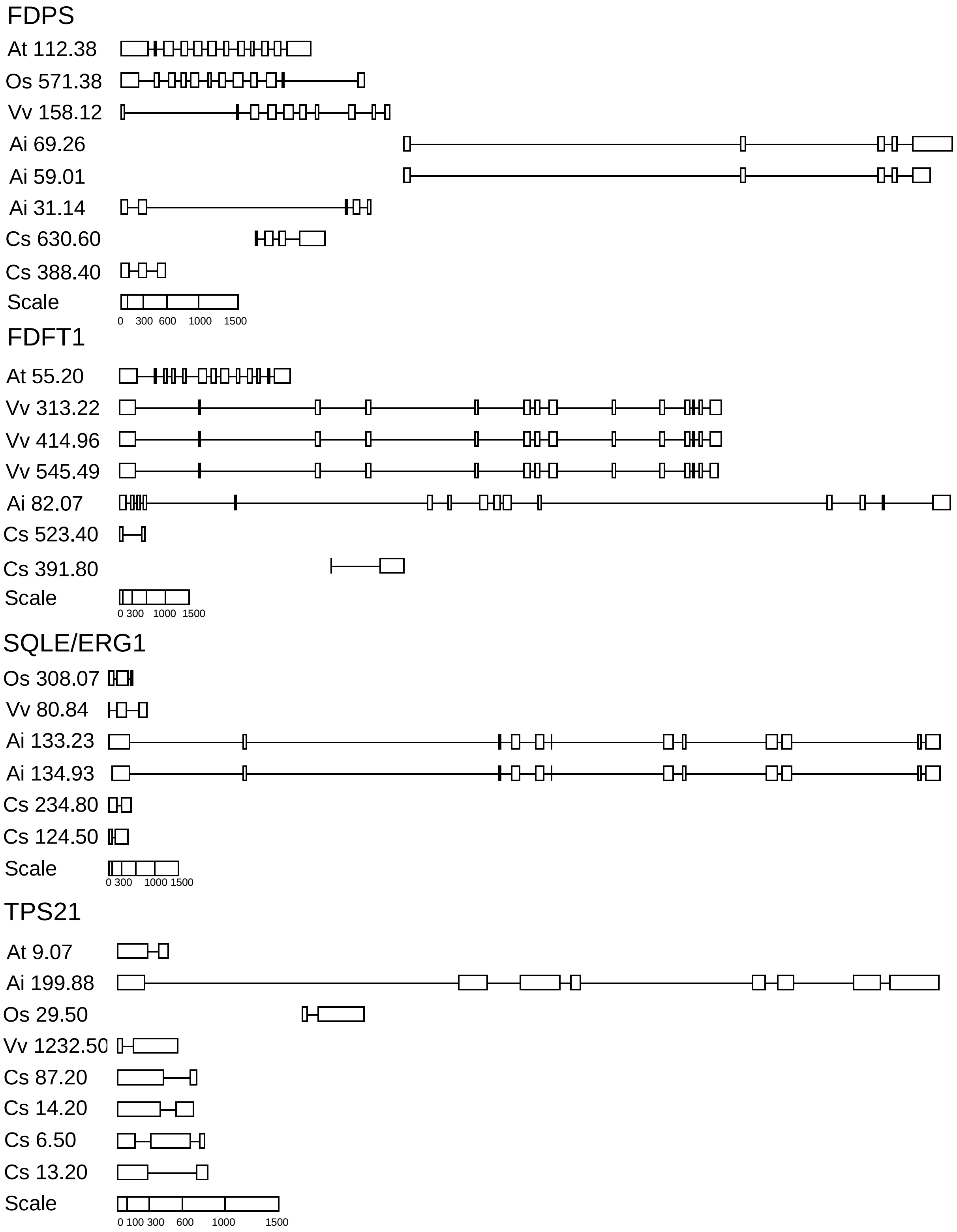
FDPS
At 112.38
Os 571.38
Vv 158.12
Ai 69.26
Ai 59.01
Ai 31.14
Cs 630.60
Cs 388.40
Scale
0 300 600 1000 1500
FDFT1
At 55.20
Vv 313.22
Vv 414.96
Vv 545.49
Ai 82.07
Cs 523.40
Cs 391.80
Scale
0 300 1000 1500
SQLE/ERG1
Os 308.07
Vv 80.84
Ai 133.23
Ai 134.93
Cs 234.80
Cs 124.50
Scale
0 300 1000 1500
TPS21
At 9.07
Ai 199.88
Os 29.50
Vv 1232.50
Cs 87.20
Cs 14.20
Cs 6.50
Cs 13.20
Scale
0 100 300 600 1000 1500

Figure S8
AT and GC density profiles in genomes and transcriptomes of neem and other plant species.
The %AT and %GC in assembled genomes and transcriptomes are plotted as density curves for neem, *O.sativa*, *A.thaliana* and *V.vinifera*.

Figure S8

Transcriptome:

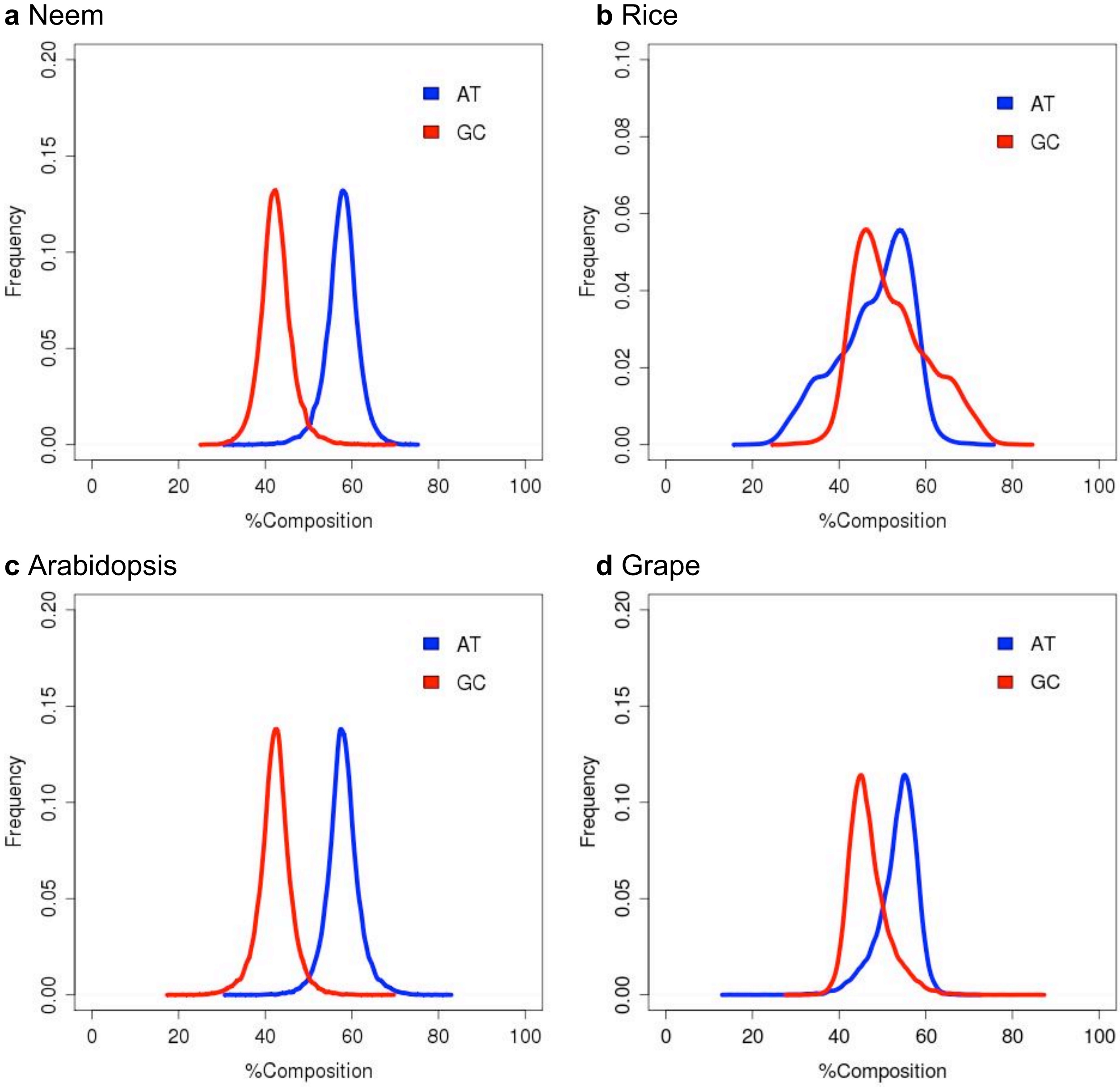

Genome:

e Neem

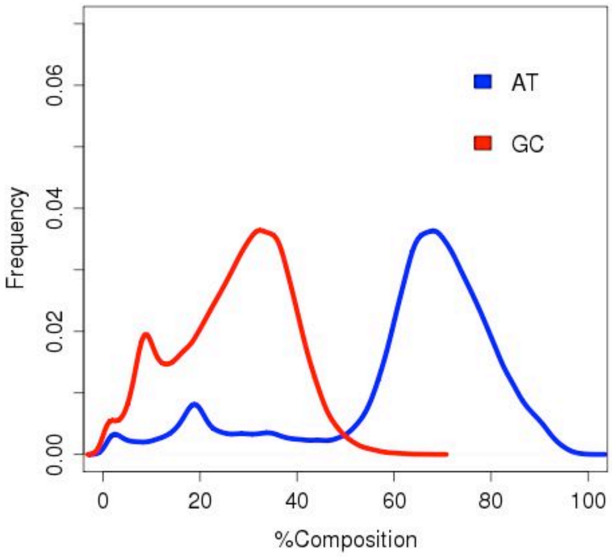

f Rice

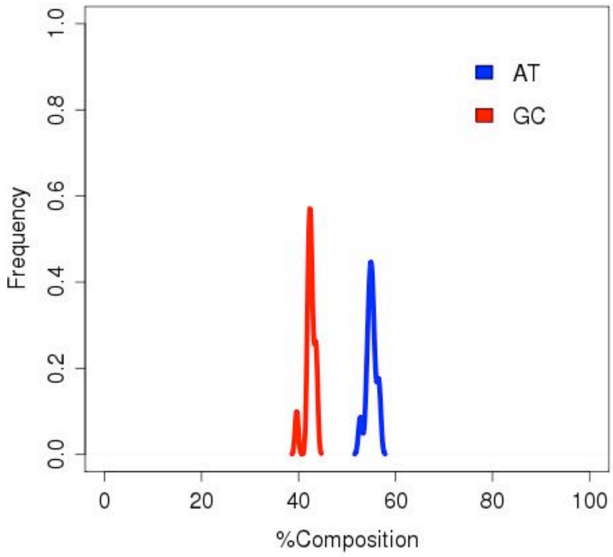

g Arabidopsis

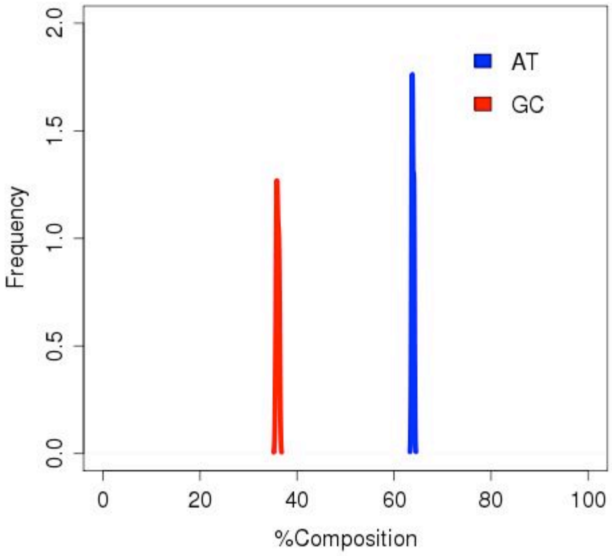

h Grape

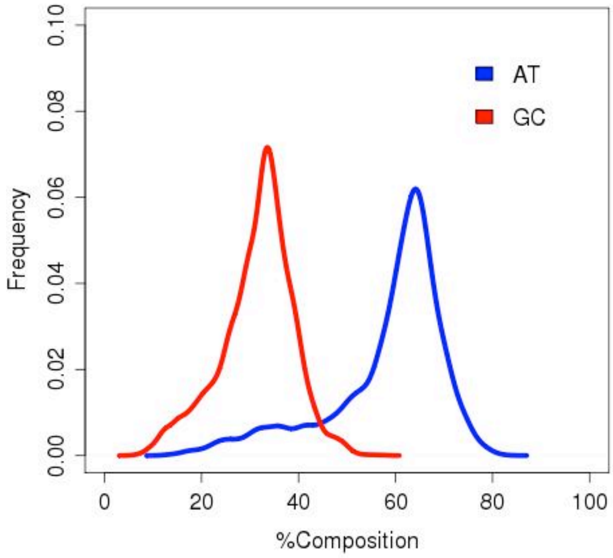

Supplement: Additional file 2 — Figure S1. kMer frequency curve. Figure S2. Frequency histograms for symmetric 4-mers. Figure S3. Genome scaffold mapping between neem and other species. Figure S4.Gene ontology based functional categorization of annotated transcripts. Figure S5. Expression density profiles of neem organ transcriptomes. Figure S6. Gene prediction statistics. Figure S7. GC-correlated expression density profiles of neem organ transcriptomes. Figure S8.AT and GC density profiles in genomes and transcriptomes of neem and other plant species. [file 1471-2164-13-464-S2.pdf]
