# Supplementary material for: A draft of the genome and four transcriptomes of a medicinal and pesticidal angiosperm Azadirachta indica
Source: BMC Genomics. 2012 Sep 9;13:464. doi: 10.1186/1471-2164-13-464 (PMC3507787; doi:10.1186/1471-2164-13-464)
Supplement: Additional file 3 — Text S1. Supplementary methods. [file 1471-2164-13-464-S3.pdf]

Text S1 (Supplementary Methods)

Sample collection, cleaning and storage

Different parts of the *Azadirachta indica* A. Juss plant were collected from a locally grown 10 yr old tree. All parts of the plant (root, leaf, stem, and flower) were collected between 10AM – 4PM of the day and between the months of February – June. Plant tissues were cleaned thoroughlly with tap water first followed by distilled water and then with 80% (v/v) ethanol. After cleaning, the organs were flash frozen in liquid nitrogen and stored at -80^{0}C until further use.

Sequencing library preparation, quality control and generation of raw reads

Short-insert paired-end library preparation

DNA was isolated using Qiagen (Hilden, Germany) kit and run on a 0.8% agarose gel to check integrity (Figure S9).

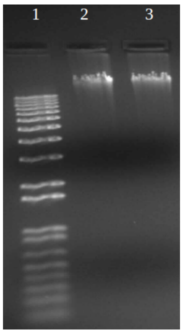

Figure S9. Agarose gel electrophoresis of genomic DNA from neem (lanes 2 & 3) along with Invitrogen 1 Kbp ladder (lane 1).

DNA library for paired end sequencing was prepared using Illumina (San Diego, California, USA) TruSeq^{TM} DNA library prep kit following manufacturers instructions. One microgram of DNA was fragmented to approximately 150 bp and 350 bp using Covaris S2 as per manufacturers instructions. Later end repair was performed to remove the 3' overhangs and fill the 5' overhangs by incubating the DNA in end repair mix containing T4 polynucleotide kinase, T4 DNA polymerase and large (klenow) fragment of DNA Polymerase I for 30 min at 30°C and purified by using Agencourt AMPure XP beads fro Beckman Coulter (Danvers, Massachusetts, USA) as per manufacturers recommendations. A-tailing of DNA was performed at 37 °C for 30 min with klenow fragment followed by ligation of TruSeq adaptors using T4 DNA ligase by incubating at 30°C for 10 min. The reaction was stopped by adding stop ligase mix and purified using

Agencourt AMPure XP beads. Size selection of adapter ligated DNA was performed using Labchip XT from Caliper (Danvers, Massachusetts, USA). The quality and quantity of the library was estimated by Nanodrop and Qubit, while the size distribution was analyzed on Agilent (Santa Clara, California, USA) Bioanalyzer using High Sensitivity DNA chips (Figure S10).

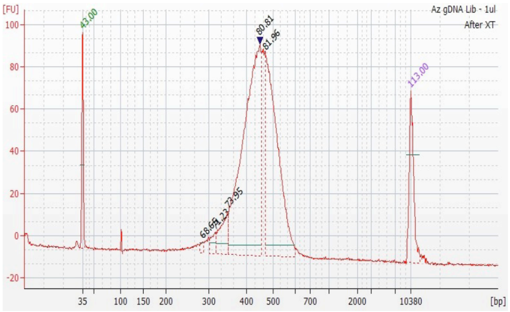

Figure S10. Agilent Bioanalyzer profile of neem 350bp insert gDNA library (short-insert and paired-end) after size selection.

Long-insert mate pair library preparation

DNA library for mate pair sequencing was prepared using Mate Pair Library v2 Sample Preparation Guide (Illumina) following manufacturer's instructions. Fifteen micrograms of DNA was used for fragmentation step. The fragmented size ranges were 1.5 kb, 3 kb and 10 kb. In each case, the DNA was fragmented separately and used for respective library preparations. Fragmentation was performed using Covaris S2 as per manufacturer's instructions and purified using QIAEX II Gel Extraction Kit. End repair was performed to remove the 3' overhangs and fill the 5' overhangs by incubating the DNA in end repair mix containing T4 polynucleotide kinase, T4 DNA polymerase and large (klenow) fragment of DNA Polymerase I for 15 min at 20°C followed by biotinylation reaction, for which 2.5 ul of biotin dNTP mix was added to the reaction and incubated at 20°C for 15 min, then placed immediately on ice, and purified using QIAEX II Gel Extraction Kit. Size selection of appropriate fragments was performed on a 0.6% agarose gel, and the DNA was extracted and purified using QIAEX II Gel Extraction Kit. The purified size selected DNA was quantified and 600 nanograms of DNA was used for circularization reaction by incubating the DNA in circularization buffer containing circularization ligase for 16 hours at 30°C. Following circularization, the remaining linear DNA fragments in the sample was removed using DNA exonuclease treatment for 37°C for 20 minutes followed by 70°C for 30 minutes. The circularized DNA was fragmented using Covaris as recommended in the protocol and purified by QIAquick PCR Purification kit. Next Dynal magnetic M-280 streptavidin beads were used to purify the biotinylated DNA fragments. The biotin label

marks the site of circularization and so, the biotinylated DNA contains the two ends of the original size selected DNA. End repair was then performed to remove the 3' overhangs and fill the 5' overhangs by incubating the DNA in end repair mix containing T4 polynucleotide kinase, T4 DNA polymerase and large (klenow) fragment of DNA Polymerase I for 30 min at 30°C. A-tailing of DNA was performed at 37°C for 30 min with klenow fragment followed by ligation of TruSeq adaptors using T4 DNA ligase by incubating at 30°C for 10 min and the reaction was stopped by adding stop ligase mix. The end repair, A-tailing and ligation reaction was carried out on the biotinylated DNA immobilized to the streptavidin beads. The adaptor ligated DNA was subjected to PCR enrichment with adaptor complementary primers for 18 cycles and size selection of adapter ligated DNA was performed using Labchip XT. The quality and quantity of the library was estimated by Nanodrop and Qubit, while the size distribution was analyzed on Agilent Bioanalyzer using High Sensitivity DNA chips (Figure S11).

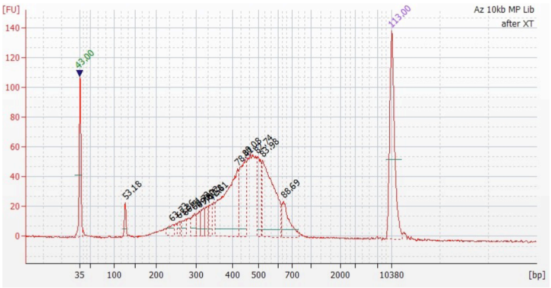

Figure S11. Agilent Bioanalyzer profile of neem gDNA 10kb Mate-pair library after size selection.

RNA-seq paired-end library preparation

Total RNA was isolated from individual organs and ran on Agilent BioAnalyzer to check quality (Figure S12).

A B C D

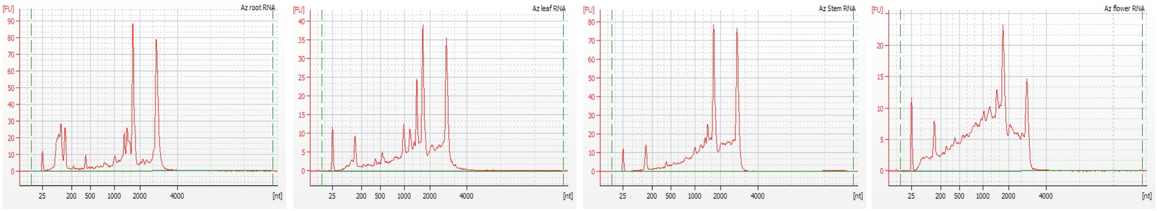

Figure S12: Agilent BioAnalyzer profiles of total RNA isolated from neem root (A), leaf (B), stem (C) and flower (D).

Four micrograms of RNA was subjected to mRNA selection by using poly-T oligo-attached magnetic beads followed by mRNA fragmentation to sizes between 100-300 nt by incubating in fragmentation mix for 8 min at 94^{0}C. First-strand cDNA was synthesized by

adding 1 ml of SuperScript II reverse transcriptase (Invitrogen) to the solution containing primed RNA and first strand master mix followed by incubation at 25^{0}C for 10 min, 42^{0}C for 50 min and 70^{0}C for 15 min. The complementary second strand cDNA was synthesized by incubating first strand cDNA in second strand master mix containing RNase H and DNA polymerase I at 16^{0}C for 1 hr. End repair was performed to remove the 3' overhangs and fill the 5' overhangs by incubating the DNA in end repair mix containing T4 polynucleotide kinase, T4 DNA polymerase and large (klenow) fragment of DNA Polymerase I for 30 min at 30^{0}C and purified by using Agencourt AMPure XP beads (Beckman Coulter) as per manufacturers recommendations. A-tailing of DNA was performed at 37^{0}C for 30 min with klenow fragment followed by ligation of TruSeq adaptors using T4 DNA ligase by incubating at 30^{0}C for 10 min. The reaction was stopped by adding stop ligase mix and purified using Agencourt AMPure XP beads. The adaptor ligated DNA was subjected to PCR enrichment with adaptor complementary primers for 15 cycles followed by cleanup using Agencourt AMPure XP beads. The quality and quantity of the library was estimated by Nanodrop and Picogreen method (Qubit, Invitrogen), while the size distribution was analyzed on Agilent Bioanalyzer using High Sensitivity DNA chips (Figure S13).

A B C D

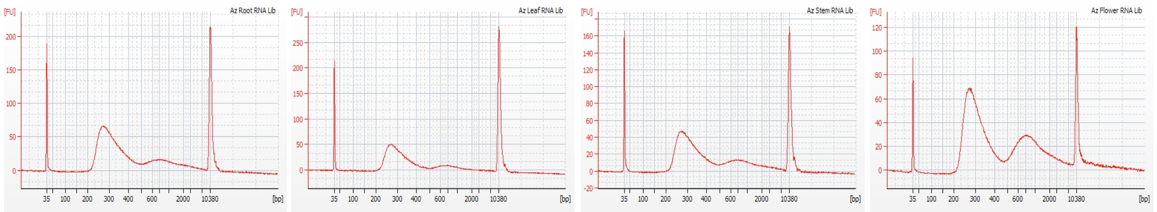

Figure S13. Agilent Bioanalyzer profiles of RNA-seq libraries for neem root (A), leaf (B), stem (C) and flower (D).

Quantification of prepared library and sequencing

Accurate quantification of prepared libraries were performed using SYBR-Green based qPCR reagents from Kapa Biosystems (Woburn, Massachutessts, USA). The qPCR results were compared to the pre-determined concentration of phiX library. Library amounts yeilding equal number of CT cycles with 6-8 picomole of commercial phiX library were seeded for cluster generation in cBot. A 72-76 bp paired-end sequencing was performed on Illumina’s Genome Analyzer IIx platform following manufacturers recommendations for short-insert genome and transcriptomes and 36 bp paired-end sequencing was performed for long-insert genome mate pair libraries.

Sequence quality control and pre-processing

Raw sequence reads (fastq) were generated using Illumina CASAVA 1.9 pipeline and

analyzed by *in-house* written scripts and checked for good quality (^{3}20 Phred score) bases in the forward and reverse reads for the entire run.

Pyrosequencing with IonTorrent Personal Genome Machine

One microgram of neem genomic DNA was sheared using Covaris S2 with 200 bp peak settings (Duty cycle 10%, Intensity 5, 200 cycles per burst) for 180 seconds. Library preparation was performed with the Ion Fragment (San Francisco, California, USA) Library Kit. DNA ends were repaired, adapters were ligated and ligated DNA was purified by using Agencount AMPure XP. Size selection was done with Invitrogen E-Gel Size Select using 2% gel. All the quality control analysis were performed using Agilent Bioanalyzer High Sensitivity DNA chip (Figure S14).

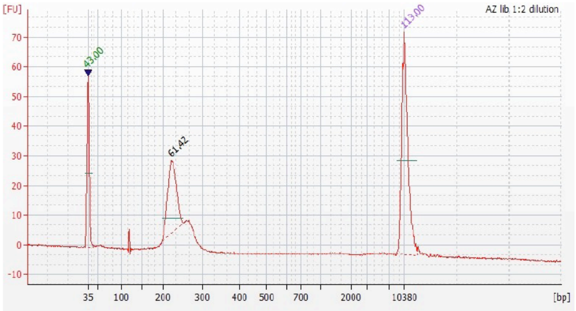

Figure S14. Agilent Bioanalyzer profile of the neem genomic DNA library.

Neem genomic DNA cloning

Fifteen micrograms of neem genomic DNA was subjected to mechanical shearing using Covaris S2. The conditions used for shearing were: bath temperature-19°C, duty cycle-20 %, intensity-0.1, cycles/burst-1,000, time-600 seconds, volume-200 ml. The sheared DNA was run in 0.8% Agarose gel for size selection of the sheared fragments (Supplementary Figure S15).

A B

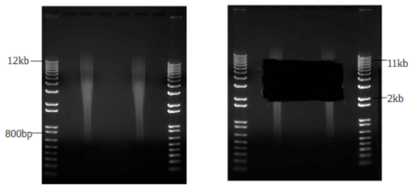

Figure S15. Agarose gel electrophoresis of neem genomic DNA after shearing (A) and after cutting the band of interest (B).

The size range of the fragment selected was from 2-11 kb (Figure S15B). The size selected fragments were gel purified using QiaExII Gel Extraction kit according to the manufacturer's instructions. The total amount of genomic DNA after gel purification was

2.1 µg. The purified fragments were subjected to end repair using the DNA terminator End repair kit provided with the Lucigen (Middleton, Wisconsin, USA) Big Easy v2.0 Linear Cloning Kit. Two micrograms of the purified fragment was mixed with 10 µl of 5X DNA terminator End Repair buffer and 2 µl of DNA terminator End Repair Enzyme and incubated at room temperature (25°C) for 30 minutes. The reaction was stopped by heat denaturation at 70°C for 15 minutes. The end-repaired fragments were purified using QiaExII Purification kit (protocol for desalting and concentrating DNA from solutions). The total amount of the end-repaired DNA after purification was 1.6 µg (33 ng/µl). Before proceeding for ligation, the integrity of the end-repaired fragment was analyzed on a 0.8% agarose gel (Supplementary Fig. S8), according to the manufacturers instructions (Big Easy v2.0 Linear Cloning Kit). The end-repaired fragments were cloned into the pJAZZ-OC vector (Blunt end) provided in the Lucigen Big Easy v2.0 Linear Cloning kit. According to the manufacturer's instruction, 396 ng of the insert was used for ligation with 100 ng of the vector. 1 ml of the 10X clone Direct Ligation Buffer and 1 µl of the Clone Smart Dna ligase (2U/ml) was added to the ligation reaction. The ligation was carried out at 23°C for 2 hours. The reaction was inactivated by heat denaturation at 70°C for 15 minutes.

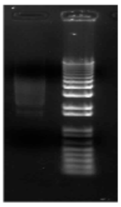

Figure S16. Neem genomic DNA post end-repair before transformation in pJAZZ-OC cloning vector.

Transformation was carried out in the Bigeasy TSA electrocompetent cells. The cells were completely thawed on ice. 1ul of the ligation reaction was added to 25 µl of the TSA Electrocompetent cells. The reaction mix was then transferred to a chilled electroporation cuvette from Eppendorf (Hamburg, Germany) (1 mm gap width, 100 ml volume, Cat# 4307-000-569). The conditions used for electroporation were:

Optimal settings
1.0mm cuvette
10µF
600 Ohms
1800 Volts
Electroporator system - Eporator, Eppendorf

Within 10 seconds of the pulse, 1ml of the Recovery media (provided in the big easy kit) was added to the cuvette and mixed thoroughly. The mixture was then transferred from the cuvette into a sterile 2 ul microfuge tube and kept in a shaker incubator at 250g for 2 hours at 37°C. After that, 100 ml of the transformed cells were plated on YT agar plates containing X-gal (20 mg/ml), IPTG (1mM) and Chloramphenicol (12.5 mg/ml). The plates were incubated at 37°C for 16 hours. The colonies obtained were screened on the basis of blue white selection. The transformed colonies were grown in 10 ml LB media containing Chloroamphenicol (12.5 mg/ml), at 37°C for 16 hours, 200g, and were later used for plasmid isolation. Plasmid DNA was restricted with HindIII to find out the positive clones for neem genomic DNA inserts (Figure S17) and were taken for capillary Sanger sequencing.

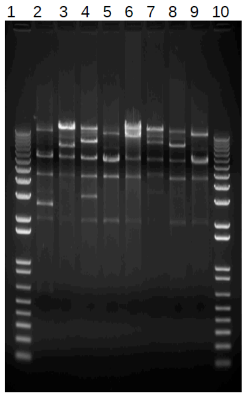

Figure S17. Agarose gel (0.8%) electrophoresis of neem gDNA cloned into pJAZZ-OC restricted with HindIII (lanes 2-9, Invitrogen 1kbp ladder lanes 1&10). Digestion of positive pJAZZ-OC (12886 bps) clones with HindIII should give a 2 Kb, 2.3Kb, 4 Kb and 4.5Kb fragments. Additional bands indicate presence of internal HindIII sites in neem gDNA clones.

Capillary Sanger sequencing

**Temperature**	**Time**	**Cycles**
96^0C	1 min	1
96^0C	10 sec	25
50^0C	5 sec	
60^0C	4 min	

Following amplification, the PCR product was purified using EDTA, Sodium acetate and ethanol precipitation. The PCR product (10 ml) was transfered to a 1.5 ml tube, and 12 ml

of mix I (milli Q + 125 mM EDTA) and 52 ml of mix II (3M Sodium acetate + ethanol) were added to it. After incubation for 15 minutes at room temperature, tubes were centrifuged at 12000 rcf for 20 min at room temperature and the supernatent was discarded. The pellet was washed with 70% ethanol, centrifuged at 12000 rcf for 15 min at room tempertaure and the supernantent was discarded. The pellet was resuspended in HiDi formamide (12-14 ml) and transfered into the 96 well plate for loading the sample. After denaturation (93°C for 3min), the plate was snap chilled, gently spun and loaded on to the 3500DX Genetic Analyzer (Applied Biosystems) for sequencing.

Genome assembly

We assembled the neem genome using SOAPdenovo[1] with a kmer size of 31 (-K 31), kmer frequency cut off of 9 (-d 9), and the asm flags set to scaffolding only (asm_flags=3) for the long insert mate-pair libraries. The libraries were ranked in an ascending order of the insert sizes, where the capillary Sanger sequencing read library was ranked last but one and the PyroSequencing read library was ranked the last. These parameter options were systematically standardized such as to yield the best scaffold N50. The frequency of kmers were used to estimate the neem genome size, after excluding the kmers with frequency=1 and after turning off the kmer frequency cutoff (-d) option. The products of the numbers of kmers and their respective frequencies were summed up in order to estimate the genome size.

The following command was used to run SOAPdenovo

SOAPdenovo all -s <file_name.config> -K 31 -d 9 -R -o <file_name.out>

Quality control by assessing Chargaff's symmetry in assembled scaffolds

The extended Chargaff's second parity rule states that an n-mer occurs as frequently as its reverse complementary counterpart[2, 3]. We compared the symmetry of 4-mers between the neem scaffolds and the *Arabidopsis thaliana* genome, and observe a tighter distribution of symmetry around 0.5 in neem (Figure S1).

Genome scaffold mapping

The neem scaffolds (with length >N50) were serially blasted using TBLASTX[4] against the genome scaffolds (with length > N50) of *C. sinensis* (scaffold N50: 251 kb), *C. clementina* (scaffold N50: 3.3 mb), *T. cacao* (scaffold N50: 473.8 kb) and *R. communis* (scaffold N50: 560 kb), and chromosomes of *A. thaliana*, *O. sativa japonica* cultivar Nipponbare, *V. vinifera* and *S. bicolor.* BLAST hits with an Expect value of 0 were plotted using Circos.

Phylogenetic analyses

The evolutionarily conserved plastid-encoded ribulose-1,5-bisphosphate

carboxylase/oxygenase large subunit (*rbcL*) and small subunit (*rbcS*) gene sequences for 23 non-*Meliaceae* plants (*Fragaria vesca*, *Prunus persica*, *Populus trichocarpa*, *Manihot esculenta*, *Ricinus communis*, *Citrus sinensis*, *Theobroma cacao*, *Arabidopsis thaliana*, *Brassica rapa*, *Vitis vinifera*, *Cucumis sativus*, *Medicago truncatula*, *Lotus japonicus*, *Glycine max*, *Solanum lycopersicum*, *Solanum tuberosum*, *Brachypodium distachyon*, *Oryza sativa*, *Sorghum bicolor*, *Zea mays*, *Selaginella moelendorffii*, *Physcomitrella patens*, *Chlamydomonas reinhardtii*) and the rbcL gene sequence for 68 *Meliaceae* plants (excluding neem) were downloaded from the NCBI Nucleotide database. The corresponding sequences in neem were extracted from Trinity-reconstructed transcripts based on their BLASTannotations. The rbcL and rbcS gene sequences from these 24 non-*Meliaceae* plant species/rbcL gene sequence from the 68 *Meliaceae* plant species were aligned using ClustalW (http://www.genome.jp/tools/clustalw) with options set to 'Slow/Accurate' and 'DNA' and -outorder=input. The individual rbcL and rbcS alignments (*.phy) were concatenated and used as input for the bootstrapping pipeline. A dataset of 100 replicates was generated for the rbcL-rbcS combined alignment using the 'Seqboot' program from Phylip version 3.69[5-7]. Corresponding Maximum Likelihood phylogenies were generated using the 'dnaml' program from Phylip package, after rooting the trees using *Chlamydomonas reinhardtii* as the outgroup species. A consensus bootstrapped phylogeny was then plotted using the 100 phylogenies using the 'consense' and 'drawgram' tools from Phylip package.

For reporting the phylogenetic trees, we followed the MIAPA standard[8]. Below are the charachteristics that is included in the information:

I. The raw sequences or character descriptions;

Excel sheet (accesion numbers) - Nucleotide db (NCBI, 28May2012) and Plant Transcript Assemblies Database (TIGR): Please see Additional File 5.

II. Sample voucher information;

Provided in the Excel sheet (wherever available) in Additional File 5.

III. Description of procedures for establishing character homology (e.g., sequence alignment);

Multiple sequence alignment using ClustalW webserver (http://www.genome.jp/tools/clustalw/) with default options. Output format: Phylip, Pairwise Alignment: Slow/Accurate, Sequence type: DNA.

IV. The sequence alignment or some other character matrix;

Attached *.phy files (Additional File 6 & 7).

V. Detailed description of the phylogenetic analysis, including search strategies and

parameter values (specific commands for the analysis program would be optimal);

Seqboot (Phylip version 3.69) for creating replicates.

Infile:clustalw.phy

No. of replicates: 100

Random number seed (must be odd): 5

Outfile:seqboot_outfile

Dnaml (Phylip version 3.69; for maximum likelihood phylogenetic tree estimation on the above 100 replicates)

Infile:seqboot_outfile

Outfile:outfile

Randomize input order of sequences? Yes (seed = 5, 1 times)

Outgroup root: Chlamydomonas reinhardtii (2a), Citrus sinensis (2b)

Analyze multiple data sets? Yes, 100 data sets

Outtree: dnaml_outtree

Consense (Phylip version 3.69) for final consensus tree.

Infile: dnaml_outtree

Treating the dnaml tree replicates as rooted, consense gave a final consensus tree with the bootstrap values.

VI. The phylogenies including branch lengths and support values (e.g., bootstrap). As provided in Figure 2a & 2b.

Repeat identification and analyses

Repeats in neem genome were identified by following the repeat pipeline as mentioned below. Repeat Modeler[9] was used to construct a novel library of repeats based entirely on the neem genome. It employs Repeat Scout[10], Tandem Repeat Finder[11] and Recon[12]. This library of repeats was used along with other known libraries from *Ricinus communis*, *Glycine max* and *Sorghum bicolor* by Repeat Masker[13] to detect and mask repeats in the neem genome based on homology to the library sequences. In addition, LTR_finder[14] TransposonPSI[15] and MITE-hunter[16] each possessing internal repeat libraries, were used to identify Long Terminal Repeats (LTRs), (retro-)transposons, and Miniature Inverted-repeat Transposable Elements (MITEs), respectively. Redundancies among the repeats detected by Repeat Masker, LTR_finder, TransposonPSI and MITE-hunter, were resolved and the final set of repeats in neem genome were used to calculate the genomic repeat content. We also used Vmatch[15] and clustered the interspersed

repeat sequences detected from all tools with a similarity of greater than 80% in repeats which are at least 200 nucleotides in length, retaining only the longest sequence from each cluster. The consensus neem repeat library was found to be 6.59% of the assembled neem genome. We further checked for presence of any protein-coding genes in our consensus neem repeat library as a quality control step, by performing BLAST analyses against the SwissProt database (www.ebi.ac.uk/swissprot/), and did not find any. Commands used to execute the above programs:

1. LTR-Finder: ltr_finder <file_name>
2. RepeatModeler: repeat_modeler <file_name>
3. RepeatMasker is run with the following three options and a consensus non-redundant set of repeats is arrived at:
    1. RepeatMasker -s -nolow -gff -norna -pa 12 <-lib library_name> <file_name>
    2. RepeatMasker -s -nolow -gff -norna -pa 12 <-lib RepeatModeler_library_name> <file_name>
    3. RepeatMasker -s -nolow -gff -norna -pa 12 <-plant_species inbuilt_library_name> <file_name>
    4. RepeatMasker -s -gff -pa 12 <file_name> [Runs with the default *RepBase* library]
4. transposonPSI: perl transposonPSI.pl <file_name>
5. MITE-hunter: mite_hunter <file_name>
6. mkvtree -db <merged_repeat_lib.fa> -indexname repeat_index -allout -v -dna -pl vmatch -supermax -l 160 -dbcluster 80 20 -v -nonredundant <clustered_repeat_lib.fa> repeat_index

Calculation of LTR insertion age

5' and 3' LTR sequences of each LTR-retrotransposon, identified by LTR finder[14], were aligned using ClustalW MPI[17]. The distances between 5' and 3' LTR sequences for each alignment were calculated using the Kimura 2-parameter distmat tool from EMBOSS package[18] The insertion ages were further calculated from these distance values according to the formula[19] $T = K / (2r)$, where T is the insertion age in years, K = Kimura distance value and r is the substitution rate per site per year (taken to be 1.3×10^{-8} as found in *O. sativa*[20].

Transcriptome reconstruction

Illumina RNA-Seq read library (a short insert (150 bp) paired-end 72 bp), for each organ was processed using Trinity[21] to reconstruct transcripts. The following command was used to run Trinity -

perl Trinity.pl --seqType fq --output Trinity --left left.fastq --right right.fastq --CPU 4 –SS_lib_type RF –paired_fragment_length 150 --run_butterfly --bflyHeapSpace 10000M

Transcript composition analyses

The A, T, G and C composition of the transcripts from the neem organs were plotted as frequency histograms. The A+T and G+C compositions of the transcripts and genome scaffolds/chromosomes were plotted as density curves for the neem, *A. thaliana*, *O. sativa* and *V. vinifera*.

The following R commands were used for plotting the frequency histograms and density curves:

1. Genome/Transcriptome GC Plot:

```
g=read.table("genome.gc")
t=read.table("transcriptome.gc")
jpeg("Neem_Genome_Transcriptome_GC.jpeg")
plot(density(g$V1,width=3),col="blue",xlab="GC%",ylab="Density",main="",ylim=c(0,0.07),
xlim=c(10,70),cex.axis=1.5,cex.lab=1.5,lwd=5)
lines(density(t$V1,width=3),col="red",xlab="GC%",ylab="Density",main="",ylim=c(0,0.07),x
lim=c(10,70),cex.axis=1.5,cex.lab=1.5,lwd=5)
legend (45,0.065"Genome",col="blue",box.col="transparent",text.col="black")
legend (45,0.055,"Transcriptome",col="red",box.col="transparent",text.col="black")
dev.off()
```

2. Exon-Intron and First Exon-First Intron GC Plot:

```
e=read.table("exon.gc")
i=read.table("intron.gc")
fe=read.table("first.exon.gc")
fi=read.table("first.intron.gc")
jpeg("Exons_Introns_All_First.jpeg")
plot(density(e$V3),col="blue",cex=30,lwd=5,xlim=c(10,70),ylim=c(0,0.10),ylab="Density",xl
ab="GC%",main="",cex.lab=1.5,cex.axis=1.5)
lines(density(i$V3),col="red",cex=30,lwd=5,xlim=c(10,70),ylim=c(0,0.10),ylab="Density",xla
b="GC%",main="",cex.lab=1.5,cex.axis=1.5)
lines(density(fe$V3),col="green",cex=30,lwd=5,xlim=c(10,70),ylim=c(0,0.10),ylab="Density
```

```
",xlab="GC%",main="",cex.lab=1.5,cex.axis=1.5)
lines(density(fi$V3),col="yellow",cex=30,lwd=5,xlim=c(10,70),ylim=c(0,0.10),ylab="Density
",xlab="GC%",main="",cex.lab=1.5,cex.axis=1.5)
legend (60,0.099,"All
Exons",col="blue",box.col="transparent",text.col="black",fill="blue",cex=1.5)
legend (47,0.089,"All
Introns",col="red",box.col="transparent",text.col="black",fill="red",cex=1.5)
legend (12.5,0.079,"First
Exon",col="green",box.col="transparent",text.col="black",fill="green",cex=1.5)
legend (12.5,0.069,"First
Intron",col="yellow",box.col="transparent",text.col="black",fill="yellow",cex=1.5)
dev.off()
```

Transcript annotation using similarity-based analyses

The transcripts identified using Trinity for each organ in Neem were serially annotated using MegaBLAST against the non-redundant nucleotide database, BLASTX against the non-redundant protein database, and MegaBLAST against the RefSeqRNA, EST (Expressed Sequence Tag) databases[4]. The unannotated transcripts were processed through the AutoFACT pipeline[22] which performed BLAST against uniref90, uniref100[23], KEGG[24-27] and cog[28] databases. The intersection analyses of annotations from various neem organs was summarized as a Venn diagram tha were drawn by using an online tool (http://bioinformatics.psb.ugent.be/webtools/Venn/).

Alignment of neem RNA-Seq reads to other plant genomes

The Neem RNA-Seq reads were aligned with assembled genomes from various other plant species (*Citrus clementina*, *Citrus seninsis*, *Glycine max*, *Sorghum bicolor*, *Ricinus cummunis*, *Zea mays, Carica papaya*, *Theobroma cacao*, *Manihot esculenta*, *Cucumis sativus*, *Prunus persica*, *Solanum lycopersicum*), using TopHat[29]. The resulting alignment (bam file) was assembled into a parsimonius set of transcripts using CuffLinks[30]. The numbers of transcripts thus obtained from CuffLinks for alignments with various plant assemblies used in TopHat are reported.

Gene prediction using GlimmerHMM

The training set for GlimmerHMM[31] was created using the transcripts from *Citrus sinensis* (39203 sequences) and *Citrus clementina* (35976 sequences). These sequences were blasted against nr database with a stringent E value cutoff of 0 to narrow down the training set to contain plant-specific gene sequences, resulting in 16385 and 11594 sequences respectively, in the *C. sinensis* and *C. clementina* training sets. Redundant

gene structures were filtered out based on homology, trimming down the two training sets further to 1719 (*C. sinensis*) and 1390 (*C. clementina*). GlimmerHMM was trained using these filtered sequences with the training modules. These resulting HMM models were used to predict gene models in the Neem genome, by GlimmerHMM. In order to identify the best HMM model for gene prediction in the Neem, we also used the most prevalent *A. thaliana* HMM model and observed a 33% reduction in the predicted gene models thus proving the sensitivity and accuracy of Citrus species based HMM models.

The commands used in the steps listed above are as follows:

1. blastn -db nr -query <transcript_file_name1> -outfmt “6 std” -out <file_name2>
2. GlimmerHMM/train/trainGlimmerHMM <genomeScaffold_file_name1> <corresponding exon_file having gene coordinates>
3. GlimmerHMM/bin/glimmerhmm_linux <Individual Neem scaffolds> <training set directory>

Gene prediction using PASA (Program to Assemble Spliced Alignments)

The Neem genome assembly and transcripts from individual organs were processed through PASA[32]. The spliced alignments of genomic scaffolds to transcripts performed using GMAP, inside PASA, are used towards gene prediction. The PASA command-line pipeline is as follows:

1. Clean the transcripts of poly-A and low-quality sequences

$PASAHOME/seqclean/seqclean transcripts.fasta

2. Map transcripts (Trinity) to genome (SOAPdenovo) using GMAP; validate alignments based on 95% alignment identity over 90% of transcript length, with consensus splice sites at intron boundaries; assemble validated spliced alignments based on genome-mapping location; group alternatively spliced isoforms into assembly-clusters.

$PASAHOME/scripts/Launch_PASA_pipeline.pl -c alignAssembly.config -C -R -g genome.fasta -t transcripts.fasta.clean -T -u transcripts.fasta

3. Generate training sets composed of gene structures of protein-coding genes with a minimum of 100 amino acids length fom PASA assemblies:

$PASAHOME/scripts/pasa_asmbls_to_training_set.dbi -M PASA_db:localhost -p user:password -g genome.fasta

The file trainingSetCandidates.pep (corresponding to all peptides translated from longest ORFs extracted from PASA assemblies) was used for comparison with GlimmerHMM.

Intersection analyses of predicted genes using GlimmerHMM and PASA

The PASA-predicted genes for each organ (root, leaf, stem and flower) were compared

with GlimmerHMM predicted genes (using *A. thaliana* and Citrus species HMM models) using BLASTX with default options and an Expect value cutoff of 10^{-10} between the GlimmerHMM predicted genes (nucleotide query) against the PASA redicted proteins for each organ (formatted as the protein database). The protein databases were created for each organ using formatdb executable in the BLAST package as follows:

1. formatdb -i <PASA_organ_predicted_protein_fasta_file> -l <log_file_name>
2. blastx -db <PASA_organ_predicted_protein_fasta_file> -outfmt “6 std” -query <GlimmerHMM_predicted_genes> -out <PASA_organ_predicted_GlimmerHMM_overlap>

The overlapping genes from each organ were pooled, filtered for duplicated and redundancies, and reported as the pooled predicted overlap between GlimmerHMM and PASA.

The sequence overlap of GlimmerHMM predicted genes using the Citrus species gene models with PASA-predicted genes was higher at an Expect value cut off of 10^{-3} and 10^{-10}. However, at more stringent Expect value cutoffs of 10^{-100} and 0, the overlap was marginally higher for genes predicted using *A. thaliana* model. This observation suggested that the prediction using *A. thaliana* inclined towards specifictity, while that using Citrus spp were more sensitive. More than 95% PASA predicted genes overlapped with GlimmerHMM predicted gene models for Neem while using the *C. sinensis* training set (with an Expect value cutoff of 10^{-10}), thus highlighting the reliability of the genes predicted by GlimmerHMM. Although *A. thaliana* is the recommended model organism for eudicots, it yields fewer predicted genes (23,397) as compared to ~34,000 yielded by Citrus species (34,624 and 34,737 in *C. sinensis* and *C. clementina*, respectively), independently hinting at potential taxonomic proximity of Citrus species to Neem.

Functional classification

The transcripts for individual organs (root, leaf, stem and flower) were processed using BLASTX[4] against the non-redundant protein database with an Expect value cutoff of 10^{-3}. The BLASTX hits were subsequently mapped using BLAST2GO[33] to their corresponding gene ontology (GO) accession and GO terms. We used the command line version of the tool, after allocating atleast 32 GB RAM for the process to run smoothly and generate the GO annotations (.annot files). The annotation files were imported into the BLAST2GO JAVA interface and processed further to yield functional annotation based on biological process, cellular components and molecular function.

The transcripts for individual organs (root, leaf, stem and flower) were mapped to KEGG pathways using KAAS (KEGG Automatic Annotation Server)[34]

using *Arabidopsis thaliana* and *Oryza sativa* as the reference sets and BBH (bi-directional best hit) method for Complete or Draft Genome.

In order to identify common gene structures among species, CDS multifasta files for *A. thaliana*, *V. vinifera* and *O. sativa* were downloaded from Phytozome (ftp://ftp.jgi-psf.org/pub/JGI_data/phytozome/v8.0/Athaliana/annotation/Athaliana_167_cds.fa.gz, ftp://ftp.jgi-psf.org/pub/JGI_data/phytozome/v8.0/Vvinifera/annotation/Vvinifera_145_cds.fa.gz, ftp://ftp.jgi-psf.org/pub/JGI_data/phytozome/v8.0/Osativa/annotation/Osativa_193_cds.fa.gz). BLASTX was performed using the fasta sequences for neem (derived from the overlapping GlimmerHMM/PASA gene structures) against the non-redundant database with enforcing the search for the green plant gene identifier (gi) lists only. The resultant gis were compared for the four species and plotted. The same fasta files were used as input to KAAS for Complete/Draft genome, using BBH method, organism list (ath, aly, osa, olu, ota, cme). The obtained KO assignments were sorted to get unique KEGG annotations. Similarly, neem PASA assemblies (for transcripts pooled from 4 organs) were input to KAAS, and unique KEGG annotations obtained.

The list of all unique KO numbers per species were used as 4 inputs to the tool at http://bioinformatics.psb.ugent.be/webtools/Venn/ to get the numbers in the parentheses.

Heatmap analyses

We chose 5 top and bottom expressed transcripts with unique KO (Kegg Orthology) assignments for each organ, and produced expression level heatmaps for chosen transcripts across all organs, individually for the top and bottom expression level transcripts. The hierarchical clustering and heatmap analyses were done using R[35]. The following R commands were used to produce the heatmaps:

1. h=read.table("<file_name>")
2. x<-as.matrix(h)
3. svg("<file_name_out.svg>")
4. heatmap(x,cexRow=0.350,scale="column")
5. dev.off()

The white to red color gradient in the heatmap indicates very low to very high expression levels. The top 5 expressed genes in various organs (Figure 5A) are involved in the following pathways/cellular compartments:

TUA6: phagosome

RPS28-1; RPL8-1: ribosome
GAPC2: glycolysis/gluconeogenesis
CcoAOMT: phenylalanine metabolism; phenylpropanoid biosynthesis; stilbeoid, diarylheptanoid and gingerol biosynthesis; flavanoid biosynthesis
ENO1: glycolysis/gluconeogenesis; RNA degradation
ADH: glycolysis/gluconeogenesis; fatty acid metabolism; tyrosine metabolism
UBC13: protein processing in ER; ubiquitin mediated proteolysis
RCI3: phenylalanine metabolism; phenylpropanoid biosynthesis
AGT3: alanine, aspartate and glutamate metabolism; glycine, serine and threonine metabolism
SNU13: ribosome biogenesis in eukaryotes
CAT3: tryptophan metabolism; peroxisome
FBA: glycolysis/gluconeogenesis; pentose phosphate pathway; mannose and fructose metabolism; carbon fixation in photosynthesis
FD1: photosynthesis
VTC2: ascorbate and aldarate metabolism
EFE: cysteine and methionine metabolism
RBCS1A: glyoxalate and dicarboxylate metabolism; carbon fixation in photosynthesis
CAB3: photosynthesis – antenna proteins
GGPS6: terpenoid backbone synthesis
TSB2: glycine, serine and threonine metabolism; phenylalanine, tyrosine and tryptophan metabolism
The bottom expressed genes (Figure 5B) are involved in the following pathways:
VPS20.2; VPS36: endocytosis
PSP: glycine, serine and threonine metabolism
CHK: glycerophospholipid metabolism
HYD1: steroid biosynthesis
SYF1; SF3B4; SR140: spliceosome
RR19; JAR1: plant hormone signal transduction
UPF3: mRNA surveillance pathway
CKX5: zeatin biosynthesis
COQ6: ubiquinone and other terpenoid-quinone biosynthesis
MEMB11: SNARE interactions in vesicular transport
LSM1: RNA degradation
PEX3-1: peroxisome

RIOK2: ribosome biogenesis in eukaryotes
ALG12: N-glycan biosynthesis
AAH: purine metabolism
CLH1; G4: porphyrin and chlorophyll metabolism
CLA1: terpenoid backbone synthesis
CAB3: photosynthesis – antenna proteins

Differential expression level analyses of genes involved in azadirachtin-A biosynthesis pathway

Raw Illumina RNA-Seq reads were downloaded for *V. vinifera* (SRP001320, Sample type: berry), *O. sativa* (SRR305463, Sample type: leaf) and *A. thaliana* (SRR314813, Sample type: leaf) species from the NCBI ftp site (http://www.ncbi.nlm.nih.gov/sra) and assembled into transcripts using Trinity (Grabherr et al. 2011; Nature Biotechnology). The corresponding FPKM values calculated by Trinity served as expression level indices for the transcripts. *C. sinensis* microarray expression data was downloaded for Sample GSM827641 (Platform ID GPL5731, Series GSE33459-Gene expression in *Citrus sinensis* (L.) Osbeck from http://www.ncbi.nlm.nih.gov/geo/query/acc.cgi?acc=GSM827641. (Sample type: RNA, leaves, healthy, 13-17 wai, biological rep 2). Probe IDs with Abs_Call 'P' were selected, and further shortlisted to ones with detection P-value <= 0.001, resulting in 14,480 unique probe IDs with the corresponding expression values. The target sequences for all the probes were downloaded from http://www.affymetrix.com/Auth/analysis/downloads/data/Citrus.target.zip. The transcripts from *A. indica* leaf were chosen for these pathway comparisons across species. The transcripts from *V. vinifera, O. sativa*, *A. thaliana* and *A. indica*, and the probe target sequences from *C. sinensis* were processed through KEGG's KAAS automatic annotation pipeline (EST job request, SBH [single-directional best hit] method for *C. sinensis* and Complete genome job request, BBH [bi-directional best hit] method for all other species), with *Arabidopsis thaliana* and *Oryza sativa* as the reference set species. The resulting pathway hierarchies were parsed to map reactions and enzymes on a composite pathway map leading to the production of neem-specific azadirachtin A. These pathways primarily involved the terpenoid backbone synthesis (ko00900), steroid biosynthesis (ko00100), and the tri-terpenoid biosynthesis (map01062). Redundancies in functional assignments to the same probe/gene with multiple expression values were resolved by chosing the assignment bearing the maximum expression value. Non-plant pathway assignments were also discarded. The enzymes are depicted as cumulative histograms of the differential expression indices, with the scale indicating the extent of differential expression plotted as

log_2ratios. The expression indices of all genes were internally normalized for each species using the expression index of elongation factor 1-alpha (EF1A), prior to calculating log_2ratio of the normalized expression index of a neem gene to that in other species.

Comparison of gene structures in neem, *V. vinifera*, *C. sinensis*, *O. sativa* and *A. thaliana*

We considered the common set of genes across all five species mapped using KEGG[25-27] in three pathways, Metabolism of cofactors and vitamins, Metabolism of terpenoids and polyketides, and Biosynthesis of other secondary metabolites. These pathways were zeroed in, in order to focus the comparison on the relative expression profiles of terpene-related genes. The transcripts coding for these genes (probe targets in the case of *C. sinensis*; http://www.affymetrix.com/Auth/analysis/downloads/data/Citrus.target.zip) were mapped to the corresponding genomes, in each species, using PASA. The intergenic exon intron structure was obtained from validated PASA genomic scaffold to transcript assemblies and the intron lengths for each gene were compared across species. The gene structures were plotted using a webtool StrDraw (http://www.compgen.uni-muenster.de/tools/strdraw/?lang=en&bscl=false).

References

1. Li R, Zhu H, Ruan J, Qian W, Fang X, Shi Z, Li Y, Li S, Shan G, Kristiansen K *et al*: **De novo assembly of human genomes with massively parallel short read sequencing**. *Genome research* 2010, **20**(2):265-272.
2. Forsdyke DR: **Symmetry observations in long nucleotide sequences: a commentary on the Discovery Note of Qi and Cuticchia**. *Bioinformatics* 2002, **18**(1):215-217.
3. Yamagishi MEB, Hirai RH: **Chargaff's "Grammar of Biology": New Fractal-like Rules**. In: *arXiv.* 2011.
4. Altschul SF, Gish W, Miller W, Myers EW, Lipman DJ: **Basic local alignment search tool**. *Journal of molecular biology* 1990, **215**(3):403-410.
5. Felsenstein j: **PHYLIP - Phylogeny Inference Package (Version 3.2)**. *Cladistics* 1989, **5**:164-166.
6. Felsenstein J: **Counting phylogenetic invariants in some simple cases**. *Journal of theoretical biology* 1991, **152**(3):357-376.
7. Felsenstein J: **Estimating effective population size from samples of sequences: inefficiency of pairwise and segregating sites as compared to phylogenetic estimates**. *Genetical research* 1992, **59**(2):139-147.
8. Leebens-Mack J, Vision T, Brenner E, Bowers JE, Cannon S, Clement MJ, Cunningham CW, dePamphilis C, deSalle R, Doyle JJ *et al*: **Taking the first steps towards a standard for reporting on phylogenies: Minimum Information About a Phylogenetic Analysis (MIAPA)**. *Omics : a journal of integrative biology* 2006, **10**(2):231-237.
9. **Repeat Modeller** [http://www.repeatmasker.org]
10. Price AL, Jones NC, Pevzner PA: **De novo identification of repeat families in large genomes**. *Bioinformatics* 2005, **21 Suppl 1**:i351-358.
11. Benson G: **Tandem repeats finder: a program to analyze DNA sequences**. *Nucleic acids research* 1999, **27**(2):573-580.

12. Bao Z, Eddy SR: **Automated de novo identification of repeat sequence families in sequenced genomes**. *Genome research* 2002, **12**(8):1269-1276.
13. **Repeat Masker** [http://www.repeatmasker.org]
14. Xu Z, Wang H: **LTR_FINDER: an efficient tool for the prediction of full-length LTR retrotransposons**. *Nucleic acids research* 2007, **35**(Web Server issue):W265-268.
15. **TransposonPSI** [http://transposonpsi.sourceforge.net]
16. Han Y, Wessler SR: **MITE-Hunter: a program for discovering miniature inverted-repeat transposable elements from genomic sequences**. *Nucleic acids research* 2010, **38**(22):e199.
17. Thompson JD, Gibson TJ, Higgins DG: **Multiple sequence alignment using ClustalW and ClustalX**. *Current protocols in bioinformatics / editoral board, Andreas D Baxevanis [et al]* 2002, **Chapter 2**:Unit 2 3.
18. **helixweb.nih.gov/emboss/html/distmat.html**
19. Paterson AH, Bowers JE, Bruggmann R, Dubchak I, Grimwood J, Gundlach H, Haberer G, Hellsten U, Mitros T, Poliakov A *et al*: **The Sorghum bicolor genome and the diversification of grasses**. *Nature* 2009, **457**(7229):551-556.
20. Ma J, Bennetzen JL: **Rapid recent growth and divergence of rice nuclear genomes**. *Proceedings of the National Academy of Sciences of the United States of America* 2004, **101**(34):12404-12410.
21. Grabherr MG, Haas BJ, Yassour M, Levin JZ, Thompson DA, Amit I, Adiconis X, Fan L, Raychowdhury R, Zeng Q *et al*: **Full-length transcriptome assembly from RNA-Seq data without a reference genome**. *Nature biotechnology* 2011, **29**(7):644-652.
22. Koski LB, Gray MW, Lang BF, Burger G: **AutoFACT: an automatic functional annotation and classification tool**. *BMC bioinformatics* 2005, **6**:151.
23. Suzek BE, Huang H, McGarvey P, Mazumder R, Wu CH: **UniRef: comprehensive and non-redundant UniProt reference clusters**. *Bioinformatics* 2007, **23**(10):1282-1288.
24. Kanehisa M, Araki M, Goto S, Hattori M, Hirakawa M, Itoh M, Katayama T, Kawashima S, Okuda S, Tokimatsu T *et al*: **KEGG for linking genomes to life and the environment**. *Nucleic acids research* 2008, **36**(Database issue):D480-484.
25. Kanehisa M, Goto S: **KEGG: kyoto encyclopedia of genes and genomes**. *Nucleic acids research* 2000, **28**(1):27-30.
26. Kanehisa M, Goto S, Kawashima S, Okuno Y, Hattori M: **The KEGG resource for deciphering the genome**. *Nucleic acids research* 2004, **32**(Database issue):D277-280.
27. Kanehisa M, Goto S, Sato Y, Furumichi M, Tanabe M: **KEGG for integration and interpretation of large-scale molecular data sets**. *Nucleic acids research* 2012, **40**(1):D109-114.
28. Tatusov RL, Galperin MY, Natale DA, Koonin EV: **The COG database: a tool for genome-scale analysis of protein functions and evolution**. *Nucleic acids research* 2000, **28**(1):33-36.
29. Trapnell C, Pachter L, Salzberg SL: **TopHat: discovering splice junctions with RNA-Seq**. *Bioinformatics* 2009, **25**(9):1105-1111.
30. Roberts A, Pimentel H, Trapnell C, Pachter L: **Identification of novel transcripts in annotated genomes using RNA-Seq**. *Bioinformatics* 2011, **27**(17):2325-2329.
31. Majoros WH, Pertea M, Salzberg SL: **TigrScan and GlimmerHMM: two open source ab initio eukaryotic gene-finders**. *Bioinformatics* 2004, **20**(16):2878-2879.
32. Haas BJ, Delcher AL, Mount SM, Wortman JR, Smith RK, Jr., Hannick LI, Maiti R, Ronning CM, Rusch DB, Town CD *et al*: **Improving the Arabidopsis genome annotation using maximal transcript alignment assemblies**. *Nucleic acids research* 2003, **31**(19):5654-5666.
33. Conesa A, Gotz S, Garcia-Gomez JM, Terol J, Talon M, Robles M: **Blast2GO: a universal tool for annotation, visualization and analysis in functional genomics research**. *Bioinformatics* 2005, **21**(18):3674-3676.

34. Moriya Y, Itoh M, Okuda S, Yoshizawa AC, Kanehisa M: **KAAS: an automatic genome annotation and pathway reconstruction server**. *Nucleic acids research* 2007, **35**(Web Server issue):W182-185.
35. R: **A Language and Environment for Statistical Computing,** http://www.R-project.org. 2011.
